# Tracing settlement dynamics in *Latium vetus*: Explainable machine learning perspectives from the Bronze to the Early iron age

**DOI:** 10.1371/journal.pone.0357054

**Published:** 2026-09-16

**Authors:** Luca Alessandri

**Affiliations:** 1 Groningen Institute of Archaeology, University of Groningen, Groningen, Netherlands; 2 Department of Scienze dell’Antichità, Sapienza University of Rome, Rome, Italy; University of Florida, UNITED STATES OF AMERICA

## Abstract

Understanding the long-term drivers of settlement location requires disentangling ecological constraints from historically contingent choices. Here I apply a Random Forest machine-learning framework, coupled with explainable AI techniques, to investigate settlement dynamics in Latium vetus (central Italy) from the Early Bronze Age to the early Iron Age (c. 2100–725 BCE). Using 108 securely dated sites and a set of environmentally constrained pseudo-absence points, I model phase-specific patterns and evaluate predictor importance through both normalized split frequencies and SHAP values, capturing structural roles as well as the magnitude and direction of effects. The results reveal a coherent diachronic transformation in the environmental logic of settlement. Early phases are primarily structured by hydrological accessibility, whereas from MBA3 onwards topographic configuration and elevation progressively dominate, marking a shift towards morphologically distinctive and defensible locations. This transition culminates in highly canalised settlement signatures during the Final Bronze Age and RMCA phases, before the RMCAIII phase signals a landscape that is increasingly saturated, hierarchically organised and functionally diversified, despite overall political stability. While the reliance on pseudo-absences and uneven sample sizes constrain predictive robustness, the approach demonstrates the value of Random Forest and SHAP as exploratory tools to expose non-linear, threshold-like relationships and to formalise long-standing archaeological interpretations. The study shows how explainable machine learning can provide a quantitative backbone for reconstructing the emergence, consolidation and transformation of territorially structured landscapes in protohistoric central Italy.

## Introduction

The area roughly bounded by the Tiber, the Aniene river, the initial foothills of the Apennines, and Monte Circeo traditionally corresponds to the region defined by ancient authors as *Latium vetus* or *antiquum* (Plin. *nat*. 3.56; Strabo 5.3.4; Serv. *ad Aen.* 1.6). Although the exact boundaries of this area vary according to different ancient sources (and their precise definition is beyond the scope of this paper), it was traditionally considered the homeland of the *Prisci Latini* or *Casci Latini* (Enn. *Ann*. 22 Sk.) and broadly coincides with the territory characterized, at the transition from the Bronze Age to the Iron Age, by the archaeological facies known as Roma-Colli Albani [1,2]. Given that this region essentially constituted the formative ground from which Rome subsequently emerged, it has long attracted considerable scholarly attention within classical and prehistoric research. Emphasis has been placed on understanding its socio-economic dynamics, often starting from the analysis of settlement patterns and associated necropoleis, with different approaches [3–10]. If we consider settlements as agents acting upon a stage defined by the landscape, understanding their behaviour necessarily involves identifying the reasons behind their foundation in a specific location. These motivations must, by definition, be rooted in the characteristics of the chosen site. However, once such features have been identified, one is confronted with the challenge of determining which among them were essential, influential, or negligible in the settlement decision-making process. To address this, and beyond relying on informed intuition (which, although valuable, is not easily quantifiable) the approach has primarily involved the use of statistical methods. For instance, by comparing expected versus observed settlement locations in relation to their distance from specific landscape features, such as rivers, springs, or bodies of water [3]. The aim of this contribution is therefore twofold. First, it seeks to explore the application of a Machine Learning algorithm, the Random Forest [11], in assigning importance values to these variables [for a similar approach, but applied to a different dataset and using partly different scoring metrics, see [12]. Second, it aims to evaluate the results considering previous research, both by comparing them with the thresholds previously obtained through conventional statistical testing and by situating them within the archaeological record of *Latium vetus* as currently understood, so that the patterns identified by the model can be assessed against the settlement, funerary and territorial evidence on which the protohistory of the region rests.

### Geographical and chronological boundary

**Fig 1:**
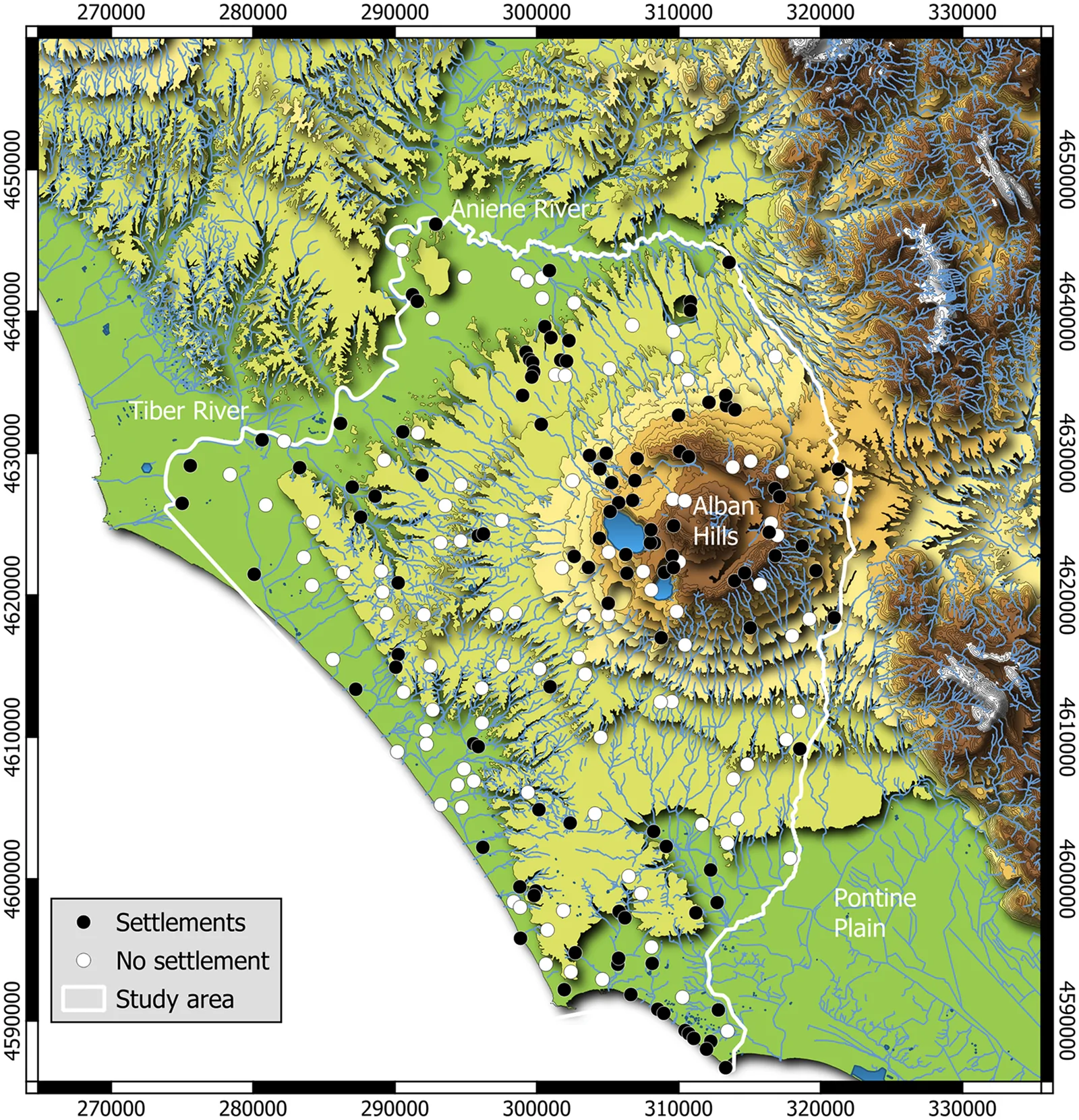
Study area. Coordinates expressed in WGS 84 UTM zone 33N (EPSG:32633). Background, elaborated by the author from TinItaly dataset (Tarquini S., I. Isola, M. Favalli, A. Battistini, G. Dotta, 2023. TINITALY, a digital elevation model of Italy with a 10 meters cell size (Version 1.1). Istituto Nazionale di Geofisica e Vulcanologia (INGV). https://doi.org/10.13127/tinitaly/1.1). The dataset is published under a CC BY 4.0 license (downloaded from https://tinitaly.pi.ingv.it/Download_Area1_1.html). Site locations, reconstructed shoreline and lakes/lagoons as determined by Alessandri (2013). Map created with QGIS 3.40, (www.qgis.org).

The area selected for the application of the method corresponds to only a portion of what was traditionally considered *Latium vetus*. Specifically, it encompasses the region between the Tiber and Aniene rivers, the Astura River, and the sea, that is, an area including the so-called Roman countryside (*Campagna Romana*), the Alban Hills (*Colli Albani*), and the coastal zone to the south of the latter (Fig 1). The exclusion of the southernmost part of *Latium vetus* (the Pontine Plain and the Volscian Mountain range) was a methodological necessity to ensure data quality for Machine Learning. The Pontine Plain is a largely buried alluvial landscape where prehistoric sites are geologically obscured [13], while the Volscian range remains largely unexplored. Including these areas would have introduced a systematic “missing data” bias (false absences correlated with specific environments).

### The dataset

**Fig 2:**
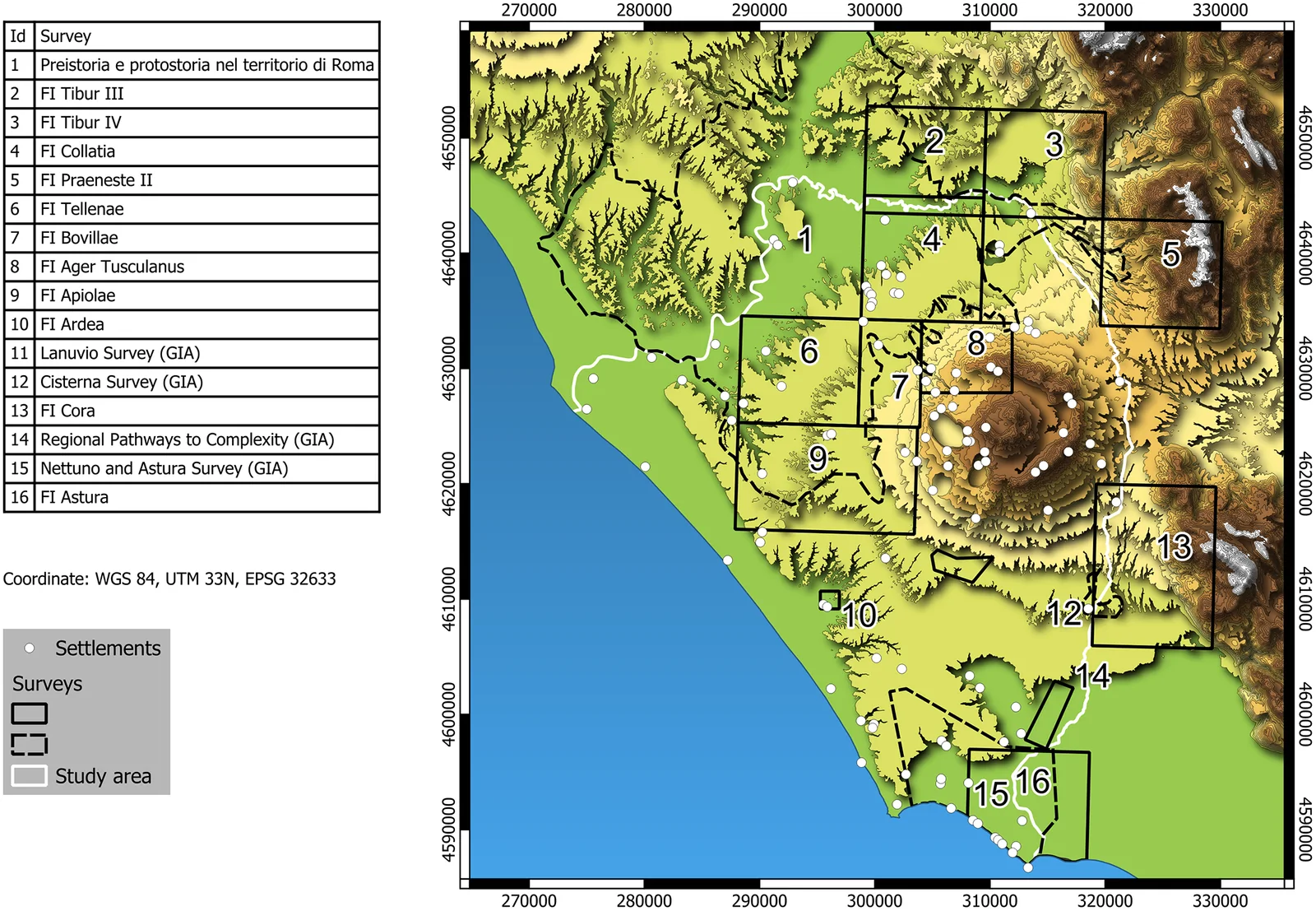
Overview of Archaeological Research Across *Latium vetus.* The distribution of systematic surveys and *Forma Italiae* (FI) volumes covers diverse landscape units (volcanic, coastal, and plain), ensuring environmental representativeness. Reconstructed shoreline for the Bronze and Iron Ages as determined by Alessandri 2013. Background, elaborated by the author from TinItaly dataset (Tarquini S., I. Isola, M. Favalli, A. Battistini, G. Dotta, 2023. TINITALY, a digital elevation model of Italy with a 10 meters cell size (Version 1.1). Istituto Nazionale di Geofisica e Vulcanologia (INGV). https://doi.org/10.13127/tinitaly/1.1). The dataset is published under a CC BY 4.0 license (downloaded from https://tinitaly.pi.ingv.it/Download_Area1_1.html). Map created with QGIS 3.40, (www.qgis.org). 1 [4,64]; 2 [65]; 3 [66]; 4 [67]; 5 [68]; 6 [69]; 7 [70]; 8 [71]; 9 [72]; 10 [73]; 11 [74]; 12 [75]; 13 [76]; 14 [77]; 15 [78]; 16 [79].

The study area, initially only partly, and later entirely under the control of early Rome, has long attracted archaeological investigation (Fig 2, caption with bibliographic references). Research has varied widely in both scope and methodological rigour, and has been conducted over a period of more than a century [3,14,15]. Eleven volumes of the *Forma Italiae* series cover sectors wholly or partially falling within this region (Fig 2, n. 2–10, 13, 16). Although invaluable as topographically organised inventories of documented archaeological evidence, these catalogues do not always represent systematic, landscape-scale surveys.

Systematic fieldwork was undertaken only in the early-1980s within the municipality of Rome, yet most of the resulting dataset remains unpublished (Fig 2, n. 1). More recently, the Groningen Institute of Archaeology has carried out small-scale systematic surveys within restricted sectors as part of successive research programmes (Fig 2, n. 11, 12, 14, 15). Outside these limited areas, available information largely derives from incidental discoveries, such as those recorded in the early twentieth century on the Alban Hills during large-scale vineyard development [3,15].

**Fig 3:**
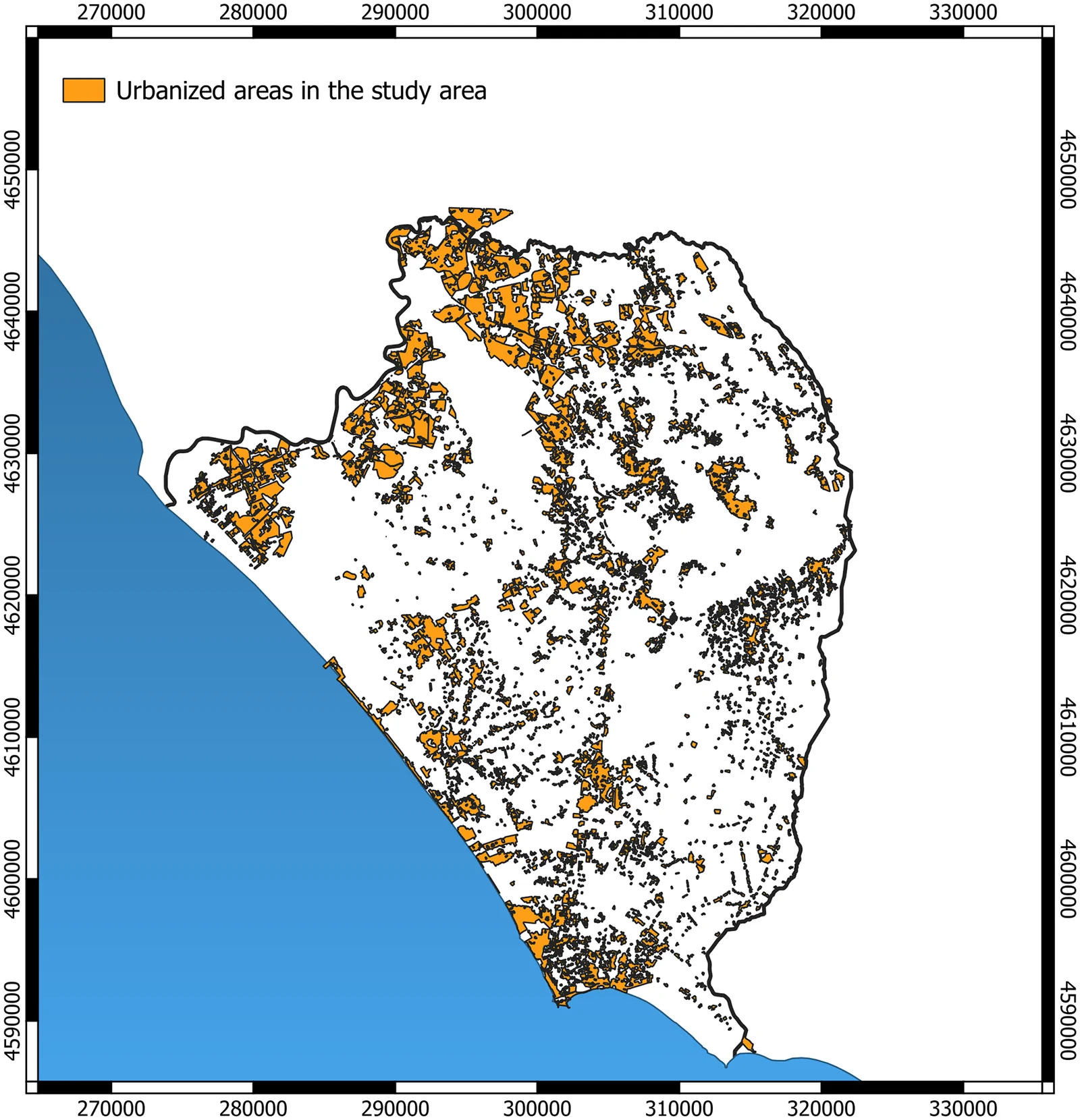
Urbanized areas within the study area. Data derived from Table B of the PTPR (Regional Territorial Landscape Plan), downloaded from Open Data Lazio (https://dati.lazio.it/dataset/ptpr-tav-b-urbanizzato/resource/4804835f-00ab-4671-8770-45d663ffcfd0), and published under a CC-BY-4.0 license (https://dati.lazio.it/licenze-di-utilizzo). Reconstructed shoreline for the Bronze and Iron Ages as determined by Alessandri 2013. Map created with QGIS 3.40, (www.qgis.org).

As a result, the statistical representativeness of this archaeological record remains uncertain. However, unlike the Pontine Plain and the Volscian range, where the paucity of data derives from structural invisibility of the evidence (buried landscapes) or lack of systematic exploration, the *Campagna Romana*–Alban Hills–coastal sector constitutes the only portion of *Latium vetus* in which archaeological visibility is sufficiently homogeneous and the archaeological record is sufficiently documented to allow meaningful comparison between environmental parameters. Furthermore, the massive urban and infrastructural expansion of the Rome metropolitan area has necessitated decades of systematic archaeological monitoring, although conducted with uneven intensity and different methodological standards over time. This development activity cuts across all landscape types indiscriminately, valleys, hillslopes, and plateaus, effectively generating a large, quasi-random sample of the archaeological record (Fig 3).

The aim of this study is therefore not to reconstruct the settlement history of the entire region, but to apply an exploratory model only where the archaeological signal is robust enough to make such comparison interpretable.

Only sites that could be assigned to the category of settlements with a reasonable degree of confidence were included in the analysis.

The chronological span considered here begins with the Early Bronze Age and extends to the end of the Roma-Colli Albani III phase. Thus, it covers a chronological range from approximately 2100–725 BCE.

The chronological phases are abbreviated as follows; all dates are approximate and given in BCE.

- Early Bronze Age – EBA (2100−1700);
- Middle Bronze Age, subphases 1 and 2 – MBA12 (1700−1400);
- Middle Bronze Age, subphase 3 – MBA3 (1400−1300);
- Recent Bronze Age – RBA (1300−1150);
- Final Bronze Age, subphases 1 and 2 – FBA12 (1150−1050);
- Final Bronze Age subphase 3 (also Roma-Colli Albani I) – FBA3 (1050−950);
- Roma-Colli Albani subphase IIA – RMCAIIA (950−880);
- Roma-Colli Albani subphase IIB – RMCAIIB (880−800);
- Roma-Colli Albani subphase III – RMCAIII (800−725).

## Methods

### Settlement parameters

The environmental variables influencing the establishment and long-term viability of a settlement in a given location are multifaceted; for the purposes of this study, the following were assessed: topographic setting, absolute altitude, and distance from the sea, from rivers, from freshwater bodies (such as lakes and lagoons), and from springs. In each case, distance refers to the shortest walking distance to the single nearest instance of the given feature type (i.e., the nearest spring, the nearest river, the nearest freshwater body, and the nearest point of the coastline).

These variables were selected because each represents a distinct environmental opportunity or constraint that may have influenced settlement decisions. Topographic setting and altitude describe the geomorphological context of a site, affecting factors such as drainage conditions, local relief, visibility and defensibility. Distance from the sea was included as a proxy for access to marine resources, navigation, maritime exchange, and coastal activities, including salt exploitation. Distance from rivers reflects access not only to freshwater but also to transport corridors and fishing resources, while at the same time entailing exposure to flooding. Distance from freshwater bodies (lakes and lagoons) represents access to lacustrine and wetland resources, including fishing, hunting of waterfowl, reeds and other exploitable aquatic resources. Springs were treated separately because they constitute reliable sources of potable freshwater, perennial and independent of river discharge and of the often brackish or stagnant nature of lakes and lagoons; unlike rivers, however, they don’t offer transport corridors. Considering these hydrological variables separately allows the analyses to evaluate whether different types of water sources exerted distinct influences on settlement location, rather than assuming a priori that they fulfilled equivalent ecological and economic functions.

With regard to the topographic setting, the categories are defined as follows (within the RF model, each category is represented by a number, shown here in parentheses):

- Isolated peak (1): an isolated elevation whose maximum height, relative to the surrounding landscape or to the nearest higher saddle, exceeds 40 m;
- Plateau (2): a flat or gently undulating surface bounded on at least three sides by steep scarps;
- Ridge (3): an elevation that is isolated on only two sides;
- Slope (4): non-isolated areas with moderate, steep, or very steep inclines (>10%);
- Hill (5): an isolated elevation whose maximum height, relative to the surrounding landscape or to the nearest higher saddle, does not exceed 40 m;
- Plain (6): non-isolated areas that are flat or gently sloping (≤10%).

These six classes constitute an ordinal, categorical variable and were assigned through expert visual inspection of the TINItaly DEM [16], which has a planimetric resolution of 10 m and a vertical RMSE between 5.89 and 5.92 m in the Lazio region, and its derived slope and hillshade layers, rather than through a fixed-radius morphometric operator. Each site was evaluated with reference to the landform unit it occupies as a whole; the quantitative thresholds specified above (relative height of 40 m; slope of 10%) function as diagnostic criteria within this morphological assessment. A fixed analysis radius was intentionally avoided because the relevant landforms operate at markedly different spatial scales. The numerical coding (1–6) follows a gradient of decreasing topographic prominence and increasing accessibility, from isolated peaks and plateaus to hills and plains.

Distance constitutes an absolute (continuous) measure and is expressed in minutes of walking time (expressed in hundredths of a second to obtain only integer values; for example, a value of 6000 corresponds to one minute of walking time), calculated using the Naismith’s rule [17,18], an empirically derived algorithm that models human movement as a function of slope frequently employed in archaeological studies [19–21]. The algorithm was implemented using the r.walk module of GRASS within QGIS 3.40 (Bratislava). The function does not incorporate additional variables that likely influenced mobility, such as vegetation, watercourses, or soil type. It provides a reasonable approximation, appropriate for the scope of this study. Distance calculations are based on a 20 m resolution DEM, derived from a landscape reconstruction of *Latium vetus* during the Bronze and Iron Ages proposed by Alessandri [3]. This reconstruction integrates geological and pedological maps, pollen and radiocarbon data, and, where available, historical cartography. Springs are taken from Ventriglia [22] and represent the modern hydrography. As such, they offer only an approximate indication of those available in protohistoric times. However, major changes are unlikely, as spring locations are governed by hydrogeological dynamics operating on timescales far longer than the protohistoric period.

**Fig 4:**
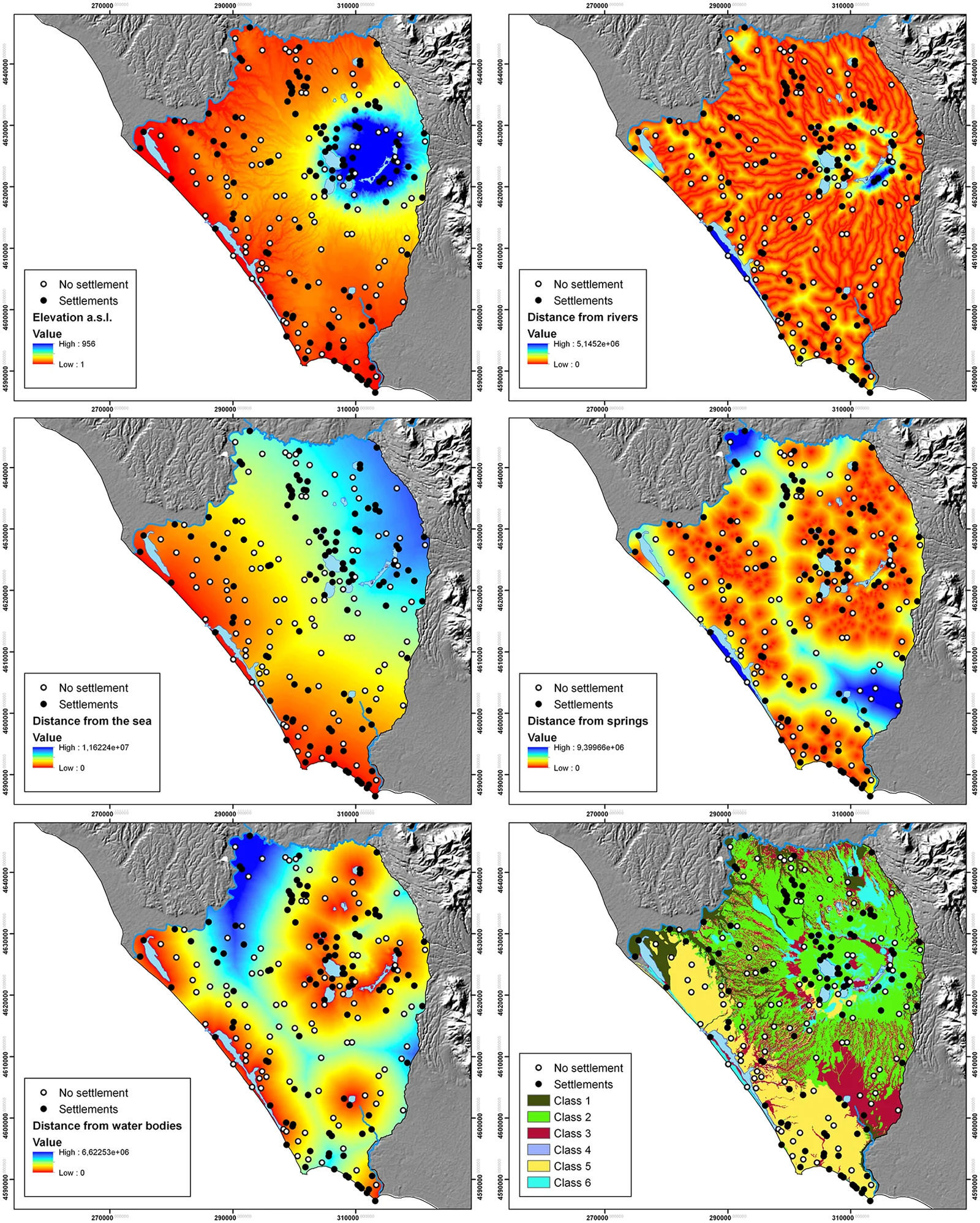
Maps of absolute elevations (above sea level), distances from rivers, the sea, springs, water bodies and classes of landscape units. Background, hillshade elaborated by the author from TinItaly dataset (Tarquini S., I. Isola, M. Favalli, A. Battistini, G. Dotta, 2023. TINITALY, a digital elevation model of Italy with a 10 meters cell size (Version 1.1). Istituto Nazionale di Geofisica e Vulcanologia (INGV). https://doi.org/10.13127/tinitaly/1.1). The dataset is published under a CC BY 4.0 license (downloaded from https://tinitaly.pi.ingv.it/Download_Area1_1.html). Map created with QGIS 3.40, (www.qgis.org).

The amount of land more or less suitable for agriculture, arboriculture, and pastoralism has also been considered, within a one-hour walking radius from the settlement’s center (Fig 4). The extent of each land class within the catchment was included because the availability of cultivable, arboricultural and grazing land defines the subsistence base a settlement could sustain, and therefore constitutes a primary constraint on where a community could establish itself durably. The model evaluates locations as if they were independent and does not account for inter-site competition or territorial exclusion, which may have influenced the realised settlement pattern in densely occupied phases.

To adequately classify soils based on the reconstructed environment, the most suitable tool would be a pedological map. However, such maps are unavailable for much of the study area. To ensure consistent comparison across regions, I therefore relied on geological maps. These were analysed in conjunction with slope classes to identify landscape units, whose agricultural potential was grouped into six categories, ranging from Class 1 (most suitable) to Class 6 (unsuitable) (Fig 4, S1 Text, S7 Table). Landscape units and their classification are detailed in the Supplementary Information. The characteristics of each class are as follows:

- Class 1: Soils suitable for all agricultural uses; deep, fertile, easy to work, with good water availability. Typically suitable for cereals, legumes, or vineyards.
- Class 2: Soils with minor limitations, such as slightly reduced depth, mild stoniness, or moderate workability; only slightly less suitable than Class 1.
- Class 3: Soils with moderate limitations, including moderate depth, stoniness, slope, or groundwater influence. Generally suitable for tree crops (excluding vineyards), unless groundwater is the limiting factor; other crops are less viable.
- Class 4: Soils with significant limitations, such as shallow depth, high stoniness, or steep slopes. Best suited for grazing; tree crops may still be viable.
- Class 5: Soils with severe limitations, including very shallow depth, very high stoniness, or very steep slopes.
- Class 6: Soils unsuitable for any form of agriculture; may support woodland.

The area used to calculate the quantity and quality of available land corresponds to the zone within one hour of walking from the settlement centre, a threshold beyond which crop agriculture becomes less feasible [23,24]. This distance (often expressed as a radius of 5 km) is commonly adopted in archaeology as the outer maximum limit of a settlement’s agricultural catchment area [e.g., 21,25–28]. The amount of each soil class constitutes an absolute measure and is expressed in square metres.

Absolute altitude constitutes a continuous, absolute measure, expressed in metres above sea level and extracted for each site directly from the TINItaly DEM [16].

The complete dataset is in the supplementary S1 Table.

### The Random Forest approach

To assess the influence of environmental variables on settlement location, a Random Forest classification model (RF) was employed. RF, an ensemble machine learning algorithm, constructs multiple decision trees and aggregates their predictions to improve accuracy and limit overfitting. Its robustness to multicollinearity, capacity to capture complex non-linear relationships, and ability to quantify variable importance make it particularly well suited to archaeological data.

**Fig 5:**
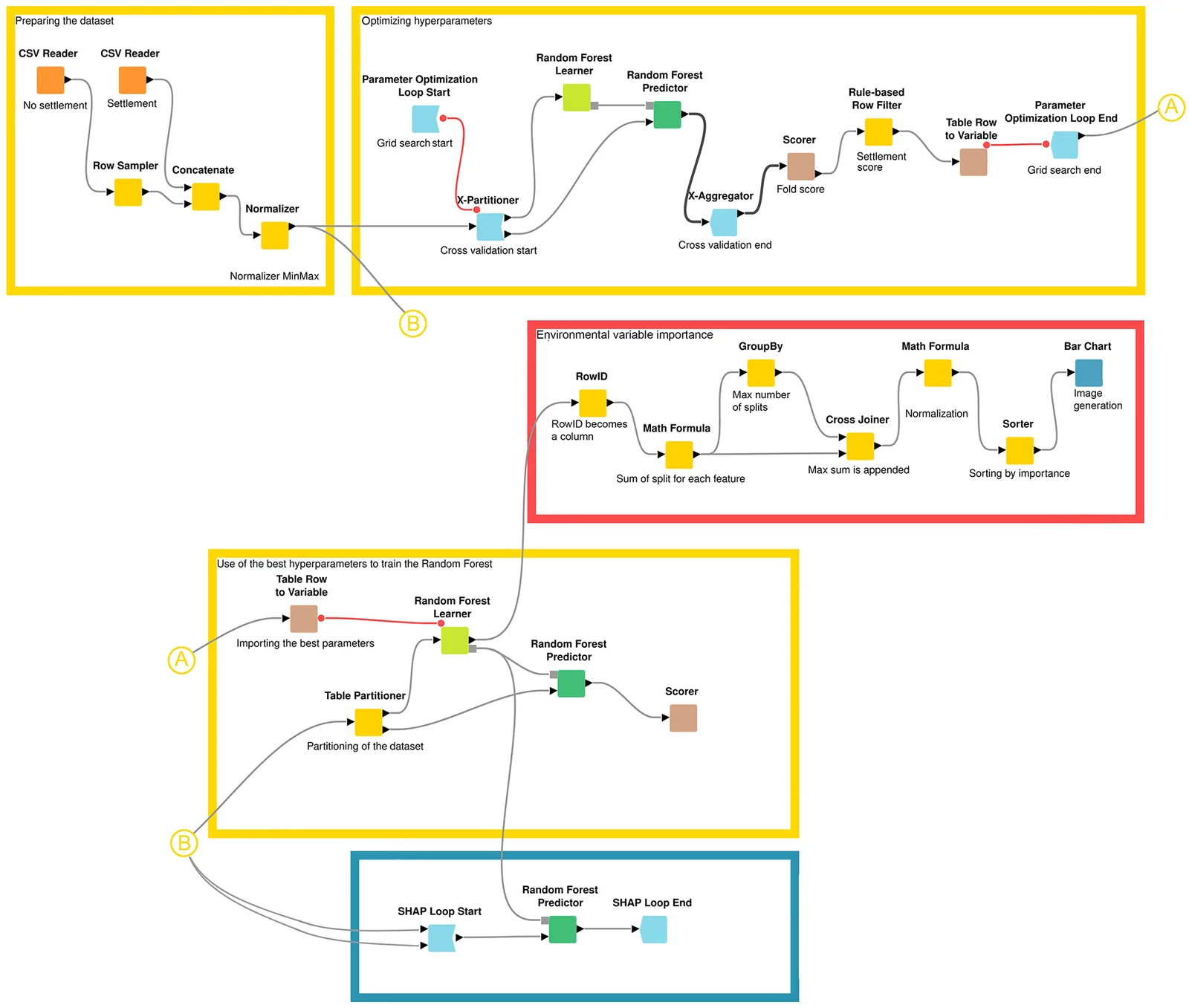
Schematic representation of the Random Forest workflow implemented in KNIME Analytics Platform 5.5.0. The diagram redraws the five stages of the analysis: dataset preparation, hyperparameter optimisation, training of the final model using the selected hyperparameters, assessment of environmental variable importance based on normalised split frequencies, and computation of SHAP values. Each block corresponds to a KNIME node and is labelled with the node name; block colours reproduce the node category colour coding used in KNIME Analytics Platform. Circled letters A and B indicate connections continuing between separate areas of the diagram.

As with all binary classification algorithms, RF requires both positive and negative examples for training. In many archaeological contexts, the available data consist exclusively of known settlement locations, resulting in a presence-only dataset. To address this limitation, pseudo-absence (or background) points were generated: locations where settlement is assumed to be absent. The inclusion of these invented points is essential, as it transforms the problem from one of presence-only modelling to a presence–absence framework. This enables the use of standard predictive modelling techniques and allows the model to distinguish the characteristics of occupied versus unoccupied locations, thereby providing more robust and interpretable insights into the factors influencing settlement patterns [29,30]. The pseudo-absences were generated randomly in QGIS, with a minimum distance of 500 meters from any known site [31]. This constraint was introduced to minimize the likelihood that undocumented settlements might share similar values for the environmental parameters previously described in this section. However, it is important to stress that these points are not truly locations where no settlement exists, but rather places where no investigation has been carried out. As highlighted by Yaworsky et al. [32], this violates the statistical assumptions underlying models such as RF and compromises the reliability of performance metrics, leading to an overestimation of the model’s predictive capacity. However, it is important to emphasise that the objective of this study is not predictive modelling, but the exploratory assessment of variable importance. The inclusion of pseudo-absence points, while problematic in a strictly predictive framework due to the risk of incorporating undiscovered sites among the negative class, does not invalidate the present analysis. Here, pseudo-absences serve solely to provide a contrasting class, enabling the Random Forest algorithm to evaluate which variables most effectively discriminate between known settlements and background locations. Nevertheless, this approach may still affect the relative ranking of variables, especially if pseudo-absence points inadvertently share similar environmental properties with actual settlements. To mitigate this risk, I compared Random Forest outputs with independent statistical analyses [3]. The results should therefore be understood not as predictions of settlement location, but as an exploratory identification of the environmental factors most strongly associated with settlement presence. The number of pseudo-absence points was selected following the recommendations of Barbet-Massin et al. [33], who showed that for machine-learning classifiers such as Random Forests the most stable and accurate predictions are obtained when the number of pseudo-absences is of the same order of magnitude as the number of presences. Accordingly, 100 pseudo-absence points were generated for 110 known settlement locations [extracted from 3], resulting in a nearly balanced dataset. The settlement and pseudo-absence point environmental parameters were initially used in their entirety and subsequently subdivided by chronological phases, considering only securely dated attributions. This allowed for the identification of phase-specific patterns. The model was implemented using the free and open-source KNIME Analytics Platform (v. 5.5.0) (Fig 5 and S2 File). Below is a description of the workflow adopted for each chronological phase and for the dataset as a whole.

### Assessment of collinearity

Although Random Forest is generally robust to correlated predictors in terms of predictive performance, strong collinearity may still affect the interpretation of variable importance, which is the primary objective of this study. Following Dormann et al. [34], I assessed collinearity among the input variables using two complementary diagnostics. First, a pairwise Spearman rank-correlation matrix was computed across all twelve predictors using the complete dataset (settlement and pseudo-absence points). Spearman’s ρ was selected because it detects monotonic (not necessarily linear) relationships and, being rank-based, is appropriate for datasets containing both continuous predictors (altitude, distances, and soil-class surfaces) and the ordinal topographic-setting variable. Second, to evaluate multicollinearity arising from the combined effects of multiple predictors, a Variance Inflation Factors (VIF) was calculated for all twelve predictors. The ordinal topographic-setting variable was included using its numerical coding (1–6), reflecting a monotonic gradient of decreasing topographic prominence. Because standard VIF calculation relies on auxiliary ordinary least-squares (OLS) regressions, which assume linear relationships among predictors, treating an ordinal variable as continuous is an approximation. However, this approach was preferred over exclusion to ensure the collinearity diagnostics covered the complete set of predictors specified in the final models.

### Optimizing hyperparameters

Hyperparameters of the RF model (parameters that are not learned by the model itself but must be set prior to training), such as the number of trees (nrModels), maximum depth (maxLevels), and minimum number of samples per leaf (minNodeSize), were optimized through a grid search procedure combined with 10-fold cross-validation. In this approach, a predefined set of hyperparameter combinations is systematically tested (the grid search procedure), which involves exhaustively evaluating model performance across all specified combinations of parameter values. Model performance is evaluated for each using repeated training and validation cycles (the cross-validation). During the cross-validation, the dataset is partitioned into ten equal subsets; in each iteration, nine subsets are used for training and one for validation, rotating through all folds. This procedure ensures that performance estimates are robust to variation in the training data and allows for the identification of the hyperparameter configuration that yields the best performance.

Within the grid search, the predictions obtained for all ten folds were pooled into a single table, from which one confusion matrix was derived. The Recall on the settlement class computed from it served as the objective function by which the candidate configurations were compared, and is reported in S4 Table as the Objective value (Recall); no other metric in that table derives from this stage. The remaining metrics reported there, Recall, Precision, Specificity, F-measure, Accuracy and Cohen’s Kappa [35], derive from the independent model described below, and are defined as follows.

Recall measures the proportion of actual positive cases that are correctly identified, thus reflecting the model’s ability to avoid false negatives. Precision quantifies the proportion of predicted positive cases that are true positives, indicating the model’s ability to avoid false positives. Specificity measures the proportion of actual negative cases that are correctly identified, capturing the model’s ability to avoid false positives and thus complementing recall. The F-measure score, calculated as the harmonic mean of precision and recall, offers a balanced measure of model performance, especially in cases where the classes are imbalanced. Accuracy measures the overall proportion of correct predictions. Cohen’s Kappa [36] indicates how much better the model’s predictions are than random guessing, by correcting for agreement that could occur purely by chance. Following the guidelines proposed by Landis and Koch [37], K values < 0.20 indicate poor agreement, 0.21–0.40 fair, 0.41–0.60 moderate, 0.61–0.80 substantial, and > 0.80 almost perfect agreement. In the context of settlement prediction, high recall reduces the risk of overlooking actual sites, while high precision minimizes the misclassification of non-sites as settlements.

For the purposes of this study, Recall on settlements was selected as the evaluation metric during the hyperparameter optimization phase. This choice, while reducing the model’s precision by allowing a higher number of false positives, ensures that virtually all true settlements are captured. For the purposes of this study, which seeks to explore the relative importance of environmental factors rather than to predict settlement location, the trade-off is acceptable: minimising false negatives prevents the underestimation of variables that genuinely contributed to settlement siting, even if this comes at the cost of incorporating some noise into the analysis.

### Use of the best hyperparameters to train the Random Forest

The procedure described above serves exclusively to identify the best-performing hyperparameter configuration. A new Random Forest was then trained from scratch with the selected configuration. In this second stage the data were partitioned once into a training set comprising 80% of the cases and a held-out test set comprising the remaining 20%; the model was fitted on the former and its predictions on the latter were passed to a Scorer node. All the remaining metrics reported in S4 Table (Recall, Precision, Sensitivity, Specificity, F-measure, Accuracy and Cohen’s Kappa) derive from that single run.

### Importances of environmental features

In the RF model, feature importance was quantified in two complementary ways. The first relies on the frequency with which each variable was used to split a node across all trees in the forest (split criterion: Gini index). These counts were then normalized, so that the importance value of each feature reflects its relative contribution to node partitioning within the ensemble. This approach highlights which variables most frequently contribute to dividing the data during model training, but it does not provide information on the direction of the association (i.e., whether a feature promotes or excludes settlement presence), nor does it necessarily correspond to true predictive influence.

The second approach is based on SHAP (SHapley Additive exPlanations), a cooperative game–theoretic framework that attributes to each feature its marginal contribution to a model’s prediction, averaged over all possible coalitions of features [38–41]. Unlike split frequencies, SHAP values capture both the magnitude and the direction of a feature’s effect on the output, thereby providing locally accurate and consistent explanations of individual predictions. In the present implementation the SHAP values are expressed in units of predicted probability and are computed for the entire dataset rather than for the test set alone. This choice reflects the fact that SHAP values are explanations of an already trained model, quantifying how each predictor contributed to the prediction returned for a given case, and are not estimates of performance; the latter remain those reported on the test data (S4 Table). Extending the computation to all cases leaves both the model and its predictions unchanged, while substantially increasing the number of observations available for the dependence plots and thus the stability of the fitted trends.

To summarise the contribution of each predictor in a single quantity, the mean absolute SHAP value was computed across all cases (N = 208, settlements and pseudo-absence points), following standard practice in the SHAP literature. Because the model is a binary classifier, the contributions towards the two classes are exact mirror images of one another; all values reported here refer to the positive class (settlement present), consistent with the sign convention adopted throughout. Taking the absolute value discards the direction of each contribution and retains only its magnitude, so that the resulting ranking measures how much each variable moves the model’s output, irrespective of whether it does so towards or away from settlement presence.

For the global analysis (all phases combined), SHAP dependence values were examined for each factor, restricted to those cases that are both real settlements (pseudo-absence points being excluded) and were assigned to the target class Yes by the model (i.e., a predicted probability of settlement presence ≥ 0.5). The analysis is thus confined to the cases in which the model correctly identified a settlement.

At the level of individual phases, where the number of observations was too limited to produce reliable dependence plots, the mean SHAP values were used instead for those factors, computed separately by phase and restricted by the same filter as well.

In summary, four distinct quantities are reported below, and they are not interchangeable. Two derive from the pooled model: the mean absolute SHAP value, computed over all 208 cases and unsigned, which ranks the predictors by the magnitude of their influence; and the SHAP dependence values, restricted to the settlements the model assigned to the target class, which resolve that influence across the range of each variable. Two derive from the nine phase-specific models, each trained on its own dataset (S11 Table): the normalized split frequencies, computed over all cases entering each model, which record how often a predictor is used to partition the data; and the mean signed SHAP values, restricted to the correctly predicted settlements of each phase, which measure the direction and strength of its effect on site prediction.

### Permutation tests

To assess independently whether the classification achieved by the models exceeds chance expectation permutation tests were performed on the pooled model and on each of the nine phase-specific ones.

For each model, the class labels (settlement / pseudo-absence) were randomly permuted across cases, thereby destroying any association between the environmental variables and site presence while leaving the distribution of the predictors unchanged. A Random Forest was then trained and evaluated on the permuted data, and the procedure was repeated 500 times to generate a null distribution of the performance obtainable in the absence of any genuine signal. The p-value is the proportion of permutations reaching a performance equal to or greater than the observed one.

Performance was measured under stratified ten-fold cross-validation, for the observed and for the permuted data alike, so that all estimates refer to cases not seen during training. Two metrics were used in parallel: Recall on the settlement class, which is the objective function of the grid search described above, and Accuracy, which is directly comparable with the figures reported in S4 Table. Because Recall is computed on the positive class alone, it rests on half the available cases and its null distribution is correspondingly more dispersed, particularly for the phases with fewest settlements; Accuracy uses all cases and provides a more stable estimate, while Recall remains the criterion by which the models were originally selected. Where the two diverge, this is stated explicitly in the results.

The tests were run on the same datasets used for the original models (S11 Table), and using the hyperparameters selected for each phase by the grid search (S4 Table).

As a complementary diagnostic, the performance obtained on the training data (here the model is fitted to all the cases of the phase and scored on those same cases, so that “training data” refers to the complete phase dataset and not to the 80% partition used for the metrics of S4 Table) was compared with the cross-validated and out-of-bag estimates. A Random Forest fits its training data closely, and does so whether or not a genuine signal is present; the difference between these quantities therefore provides an indication of the extent to which the reported performance reflects generalisable structure rather than memorisation of the training cases.

The tests were implemented in Python 3.13.14 using scikit-learn, as the loop-based construction required for repeated label permutation is impractical in KNIME. Absolute values are therefore not strictly identical to those in S4 Table, which derive from a single held-out partition in KNIME rather than from repeated cross-validation in a different software implementation; the comparison between observed and permuted performance, which is the object of the test, is unaffected, since both are computed under identical conditions. The script is provided as supplementary material (S1 File).

## Results

Of the 110 settlements originally compiled, two were excluded from the analyses. Site 165 was removed because it has subsequently been re-dated to the Eneolithic [42] and therefore falls outside the chronological range considered here. Site 181 was removed because the distances from rivers and from springs could not be calculated for this location, and records with missing values were filtered out prior to modelling. All results reported below are therefore based on 108 settlements and 100 pseudo-absence points (S1 Table).

The results are presented in order of decreasing analytical scope. I first report the collinearity diagnostics, which establish that all twelve predictors could be retained, and the performance of the models together with the permutation tests, which delimit how far each of them can be interpreted at all. I then turn to the pooled model, examining first the overall ranking of predictors by mean absolute SHAP value and then, through the dependence plots, the behaviour of each predictor across the range of its values. The phase-specific models are considered last, reporting the normalized split frequencies and the mean SHAP values for each chronological phase.

### Collinearity diagnostics

The Spearman rank-correlation matrix (S8 Table and S1 Fig) shows that four predictor pairs exceed the |0.7| threshold: Sea–C5 (ρ = −0.84), C2–C5 (ρ = −0.79), Altitude–Sea (ρ = 0.75) and Sea–C2 (ρ = 0.75). All four correspond to a single coast-to-inland gradient, along which distance from the sea, altitude, and the relative extents of soil classes C2 and C5 co-vary. A fifth pair, Altitude–C2, approaches the threshold at 0.69 and belongs to the same gradient. Among the soil classes specifically, only the C2–C5 pair exceeds the threshold, while outside this pair, no inter-class correlation reaches the |0.7| threshold; the remaining soil–soil correlations are moderate at most (the strongest being C2–C6 at ρ = +0.44 and C5–C6 at ρ = −0.41), indicating that the quasi-compositional structure of the soil variables produces only weak-to-moderate dependence. The Variance Inflation Factors confirm this at the multivariate level (S9 Table): all values fall below the conventional critical value of 10, with a maximum of 5.25 for Sea, followed by Altitude (4.16) and C5 (3.68). The ordinal topographic-setting variable returns one of the lowest values of the set (1.29), so that the approximation involved in including it has no material bearing on the diagnostic. Although four pairwise correlations exceeded the adopted Spearman threshold, all VIF values remained below the adopted threshold, indicating that these correlations did not compound into problematic multivariate collinearity. Consistent with this, the four pairs all involved variables tracking a single coast-to-inland environmental gradient rather than independent redundancies. No variable exclusion was therefore warranted, and all predictors were retained in the subsequent analyses.

### Model performance

The performance metrics of the models are reported in S4 Table. For the pooled model, Accuracy is 0.71 and Cohen’s Kappa 0.43, on a held-out partition of 42 cases. Among the phase-specific models Accuracy ranges from 0.62 (MBA12) to 1.00 (FBA12), and Kappa from 0.21 (RBA) to 1.00 (FBA12); these figures, however, must be read against the size of the partitions on which they are calculated, which comprise between four cases (EBA) and fourteen (RMCAIII), so that a single misclassification alters accuracy by between 7 and 25 percentage points. The perfect classification obtained for FBA12 corresponds to three true positives and three true negatives out of six test cases.

The permutation tests (S12 Table) place these results on firmer ground. In the phase-specific models the null distributions centre close to 0.50 for both metrics, as expected of their balanced design; in the pooled model, where settlements slightly outnumber pseudo-absence points (108 against 100), they centre at 0.503 for accuracy and 0.543 for recall.

The pooled model performs significantly better than chance on both criteria, at the resolution limit of the procedure: Accuracy 0.716 (p = 0.002) and recall 0.730 (p = 0.002), with none of the 500 permutations reaching the observed values. Its cross-validated accuracy is virtually identical to the figure obtained on the held-out partition (0.716 against 0.71), indicating that with 42 test cases the latter was already a reliable estimate.

Among the phase-specific models, four return performance significantly above chance on both metrics: FBA12 (Accuracy 0.867, p = 0.002; Recall 0.850, p = 0.008), RMCAIIA (0.860, p = 0.002; 0.883, p = 0.002), RMCAIIB (0.827, p = 0.002; 0.817, p = 0.002) and EBA (0.750, p = 0.048; 0.800, p = 0.044). For the first three, no permutation reached the observed value on either metric.

The result for FBA12 is of particular relevance. The perfect classification reported in S4 Table cannot be taken at face value, since it rests on six test cases; under repeated cross-validation the same model returns 0.867 against a null mean of 0.487. The separation between settlements and pseudo-absences in this phase is therefore genuine, but its strength is more modest than the held-out figure suggests.

Three phases, MBA3, FBA3 and RMCAIII, are significant on Accuracy (p = 0.036, 0.008 and 0.046) but not, or only marginally, on Recall (p = 0.182, 0.052 and 0.116). Since Recall is computed on the settlement class alone, and therefore on half the available cases, its null distribution is the more dispersed of the two; this divergence is best read as indicating a signal that is real but weaker than in the four models above, rather than as a contradiction between metrics.

Two phases fail to outperform chance on either criterion. MBA12 returns an Accuracy of 0.575 (p = 0.293) and a Recall of exactly 0.500 (p = 0.605), identical to the null mean; RBA performs no better, with an accuracy of 0.545 (p = 0.315) and a Recall of 0.400 (p = 0.820) that falls below the null expectation. For these two phases the environmental variables considered here do not discriminate settlement locations from pseudo-absences.

The training–validation comparison points in the same direction. Training accuracy is high throughout (0.864–1.000), as expected of a Random Forest irrespective of whether a genuine signal is present, so that high values on training data are uninformative in themselves. The gap between training and cross-validated accuracy is smallest for RMCAIIA (0.040), FBA3 (0.100) and FBA12 (0.133), and largest for RBA (0.455) and MBA12 (0.300), the same two phases identified as non-significant by the permutation tests. The pooled model sits in an intermediate position (0.284), as expected of a model trained across the whole chronological range. Out-of-bag estimates track the cross-validated ones closely throughout, confirming that the latter are not an artefact of the particular fold partition.

Taken together, these results delimit the interpretive scope of the analyses that follow. The pooled model, on which the dependence plots rest, discriminates settlements from pseudo-absences well above chance. Among the phase-specific models, those for FBA12 and for both earlier RMCA phases are robust, as is EBA despite its small sample; MBA3, FBA3 and RMCAIII are moderately supported; and MBA12 and RBA are not supported. The SHAP patterns described below for these last two phases should accordingly be regarded as descriptive of model behaviour rather than as evidence of environmental determinants of settlement.

### Different models

The ALL model, which aggregates all chronological phases, attains moderate predictive performance. Yet this model necessarily blends heterogeneous phase-specific patterns that do not correspond to a single territorial process. Statistically, its discriminant function represents an average response to environmental gradients drawn from distinct contexts. As a result, the model highlights those predictors that retain discriminative power across the full temporal sequence: even under internal heterogeneity, Random Forests tend to amplify variables that remain informative over large regions of the feature space. In this respect, the ALL model provides a robust indicator of the overall stability of each predictor in distinguishing sites from non-sites throughout the protohistoric period.

Using both the aggregated ALL model and the phase-specific models therefore enables a clear analytical distinction: the ALL model captures the intrinsic, temporally stable importance of predictors within the wider protohistoric landscape of *Latium vetus*, while the phase-resolved models illuminate the historical dynamics and shifting settlement strategies that unfolded within each individual period.

### Mean absolute SHAP values, pooled model

**Fig 6:**
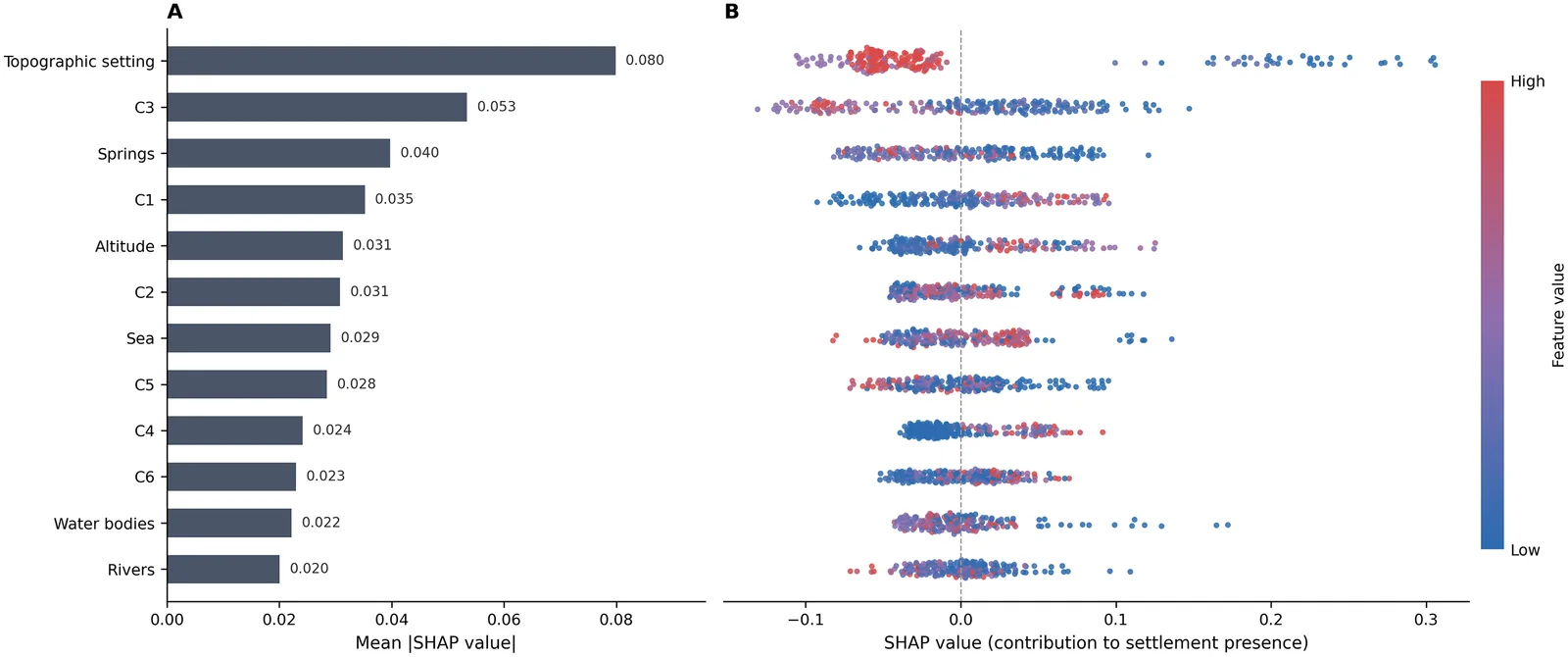
Global importance of the environmental variables in the pooled Random Forest model. (A) Mean absolute SHAP value for each predictor, computed over all 208 cases (settlements and pseudo-absence points), and ranked in decreasing order; the same ordering is used in both panels. (B) Beeswarm plot of the individual SHAP values underlying those means: each point is a single case, positioned along the x-axis according to its contribution and coloured according to the value of the variable, from low (blue) to high (red). Values are stacked vertically where they overlap, so that the width of each band indicates the local density of cases. SHAP values refer to the positive class (settlement present) and are expressed in units of predicted probability. The dashed grey line marks SHAP = 0. For topographic setting the colour scale refers to the ordinal code (1 = isolated peak to 6 = plain), so that low values correspond to the most prominent landforms. Underlying values are reported in S13 Table.

Mean absolute SHAP values for the pooled model are reported in S13 Table (Fig 6A). Topographic setting ranks first by a wide margin (0.080), some 50% above the second-ranked predictor and four times the last. C3 follows (0.053), then springs (0.040) and C1 (0.035). The remaining eight predictors occupy a narrow band between 0.020 and 0.032, within which the differences are too small to sustain a meaningful ordering.

Two features of this ordering call for comment. First, it confirms at the global scale what the phase-specific analyses show repeatedly: topographic setting is the single predictor whose influence is of a different order from the rest. Second, and more unexpectedly, three of the four hydrological variables occupy the lower half of the ranking, with rivers last of the twelve. This should not be read as evidence that watercourses were unimportant. A mean absolute SHAP value compresses a variable’s entire behaviour into a single number, and rivers act as a strictly local attractor: their contribution is substantial for the forty-eight settlements within six minutes’ walk and negligible across the remaining fifty-two (see the dependence plots below), so that the average understates the effect where it operates and overstates it where it does not. The same applies, in a different form, to the sea, whose strong coastal signal is confined to nine settlements (see the dependence plots below). Water bodies behave in the same way and more sharply still: their contribution is concentrated in a small group of settlements lying within a few minutes of a lake or lagoon, against a median distance of an hour across the dataset as a whole. Variables of this kind rank low precisely because their influence is spatially restricted rather than diffuse, and the aggregate ranking is by construction unable to express it.

The beeswarm plot (Fig 6B, S13 Table) makes the point directly, since it displays the full distribution of contributions from which each mean is derived, with each case coloured according to the value of the variable. The contrast is starkest for topographic setting, where the two colour groups separate completely: all thirty-nine cases on plateaux, isolated peaks or ridges fall to the right of zero and all one hundred and sixty-nine on hills, plains or slopes to the left, with no overlap between them. For the hydrological predictors the points are ordered by feature value in a consistent direction. The fifteen largest contributions of rivers come from settlements at a median distance of 0.3 minutes from a watercourse, against a median of 6.1 minutes across all cases, and mean contributions decline steadily from +0.020 in the nearest quartile to −0.014 and −0.011 in the two most distant. Water bodies behave likewise and more sharply, the fifteen largest values corresponding to a median distance of 4.2 minutes against 60.5 overall, with only the nearest quartile returning a positive mean (+0.029). Springs show the steepest gradient of the four, from +0.059 in the nearest quartile to −0.051 in the third. The sea is the exception: its largest contributions likewise come from immediate coastal locations, but the quartile means are not monotonic (+0.004, −0.021, + 0.013, + 0.004), reflecting a bimodal structure. The low position of these variables in the ranking therefore does not indicate an absence of effect, but its confinement to a restricted portion of their range, over which the direction of the effect is nonetheless stable. All quartile figures reported here are computed over the complete set of 208 cases.

It should also be borne in mind that this ranking derives from the pooled model, which aggregates all chronological phases and cannot express the diachronic reweighting between hydrological and topographic criteria documented below and it is the dependence plots examined in the following section that make this structure visible.

### SHAP dependence plots, pooled model

**Fig 7:**
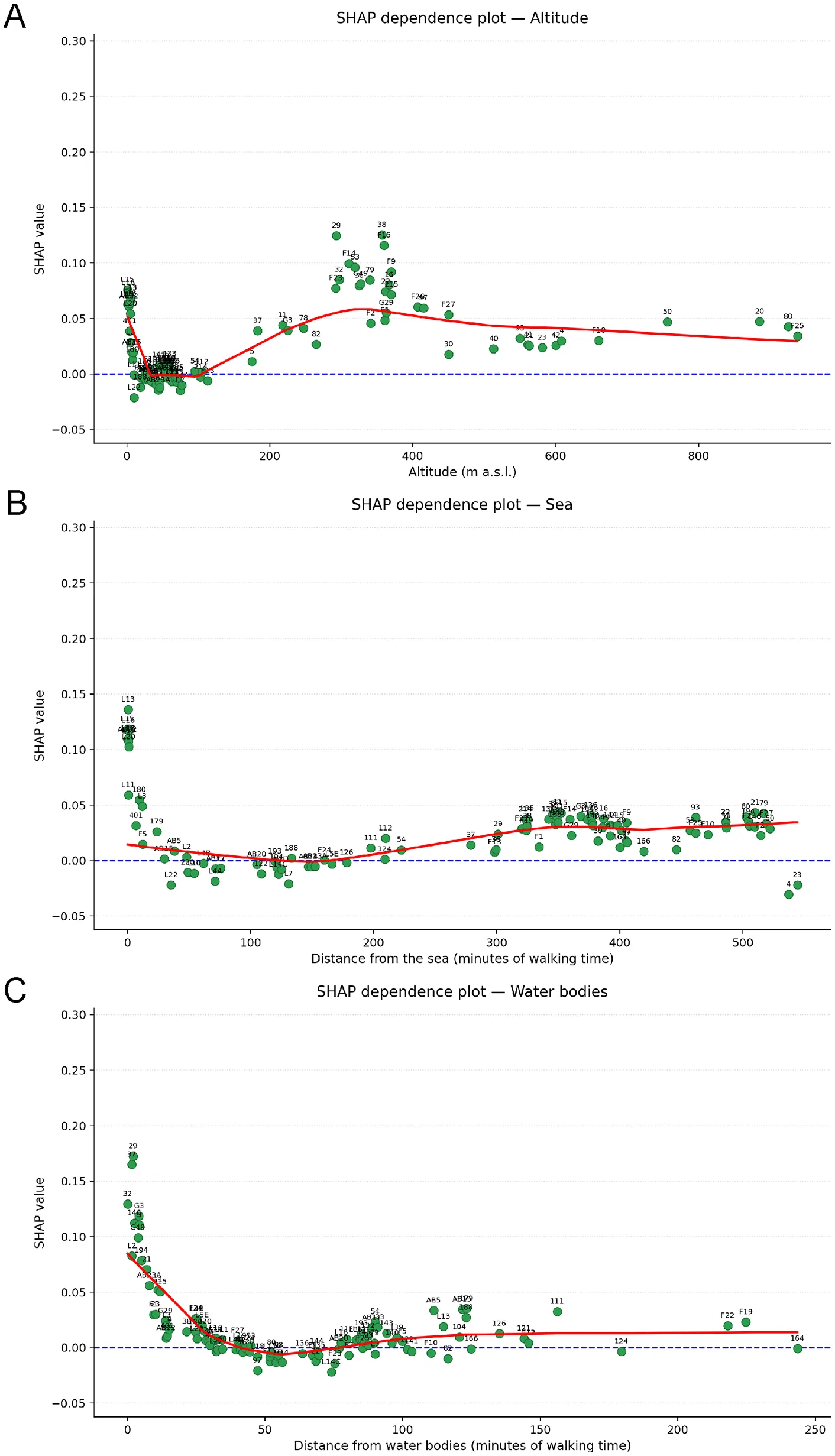
SHAP dependence plots for the main environmental variables in the Random Forest model: altitude, distance from the sea and distance from water bodies. SHAP values are expressed in units of predicted probability: they measure the shift in predicted probability of settlement presence attributable to each variable, relative to the model’s mean prediction. The dashed blue line marks SHAP = 0, and the red line shows the LOESS trend (frac = 0.5).

**Fig 8:**
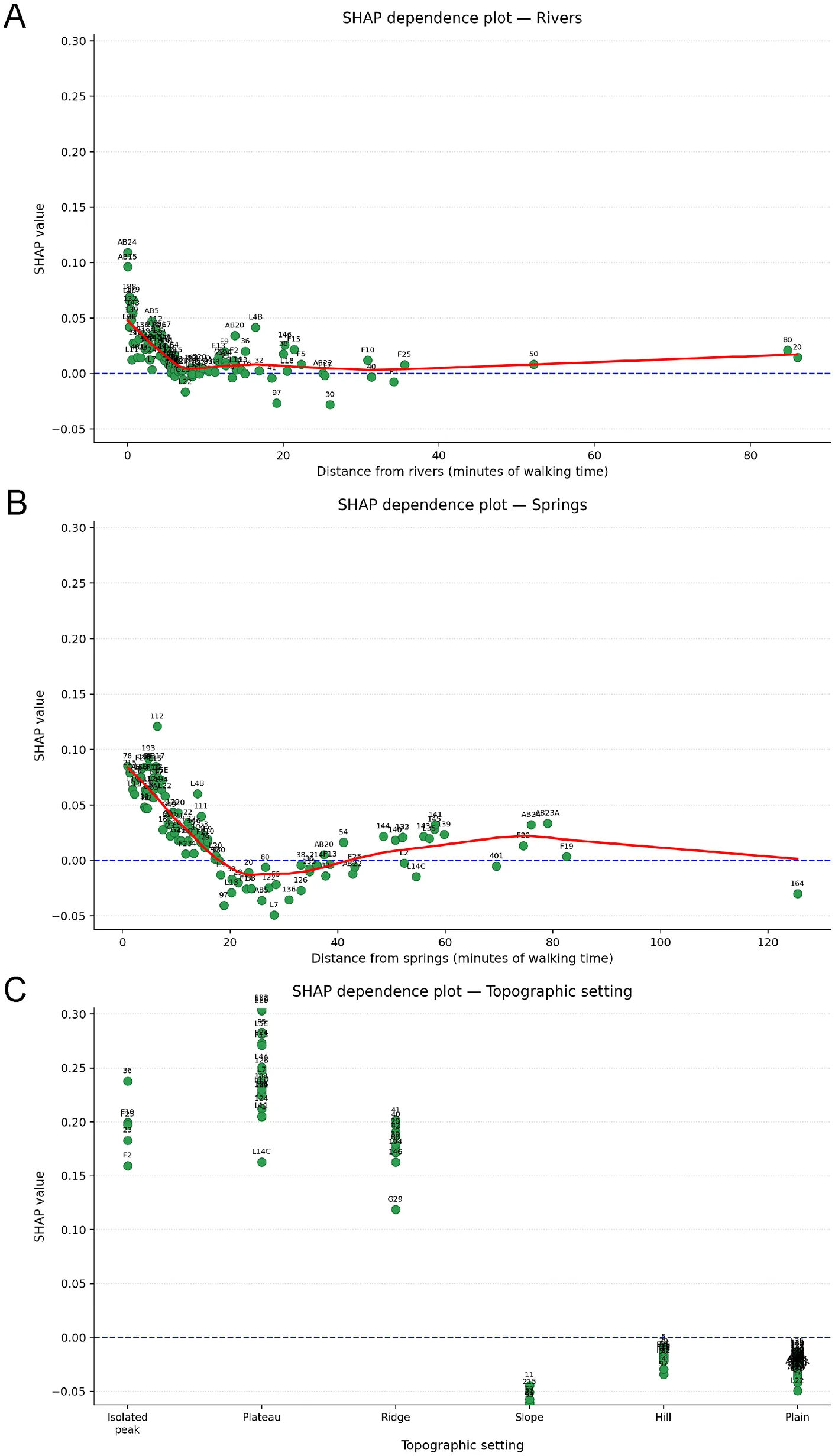
SHAP dependence plots for the main environmental variables in the Random Forest model: distance from rivers, distance from springs and topographic setting. SHAP values are expressed in units of predicted probability: they measure the shift in predicted probability of settlement presence attributable to each variable, relative to the model’s mean prediction. The dashed blue line marks SHAP = 0, and the red line shows the LOESS trend (frac = 0.5).

**Fig 9:**
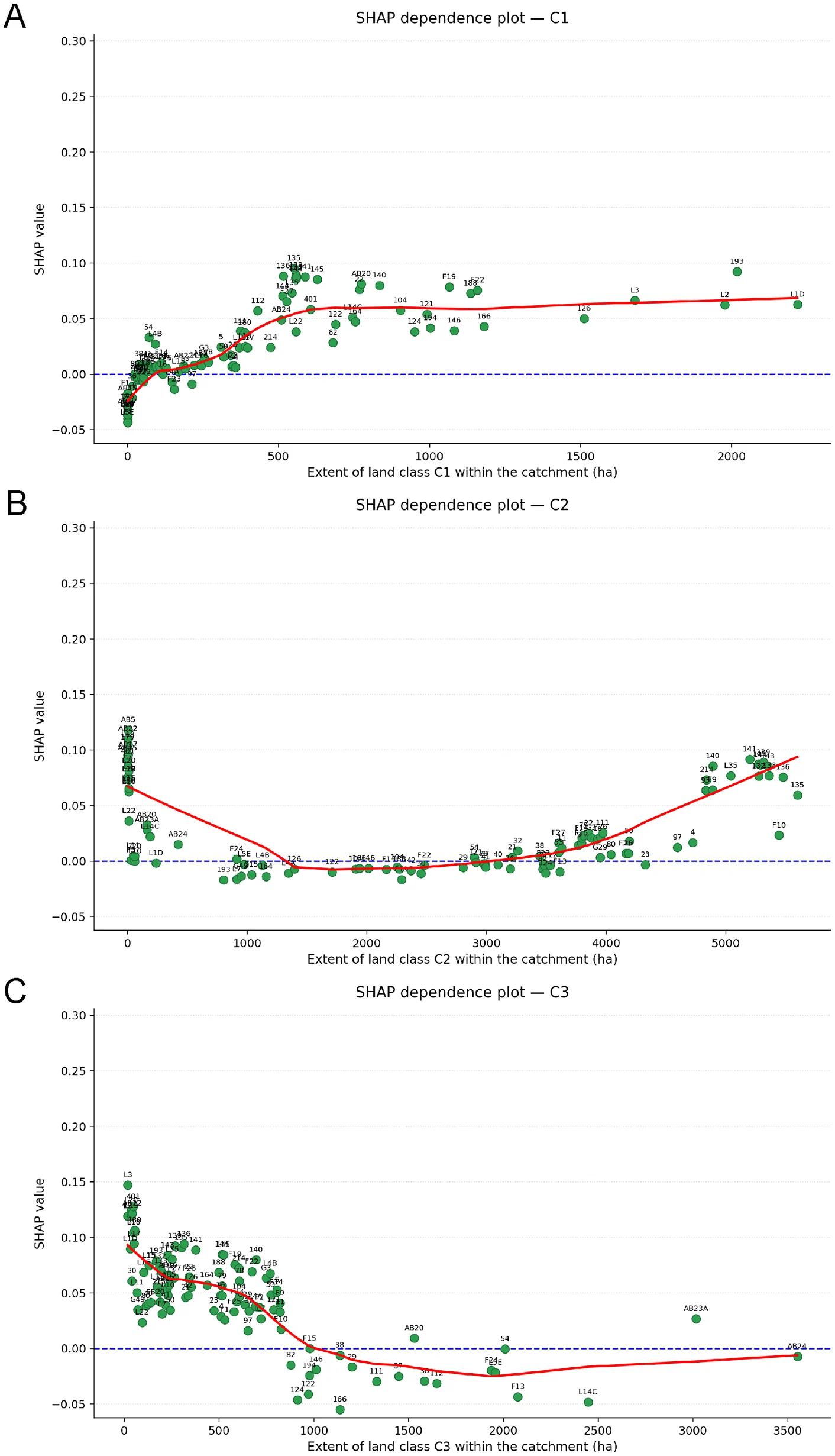
SHAP dependence plots for the soil classes in the Random Forest model: C1-3. SHAP values are expressed in units of predicted probability: they measure the shift in predicted probability of settlement presence attributable to each variable, relative to the model’s mean prediction. The dashed blue line marks SHAP = 0, and the red line shows the LOESS trend (frac = 0.5).

**Fig 10:**
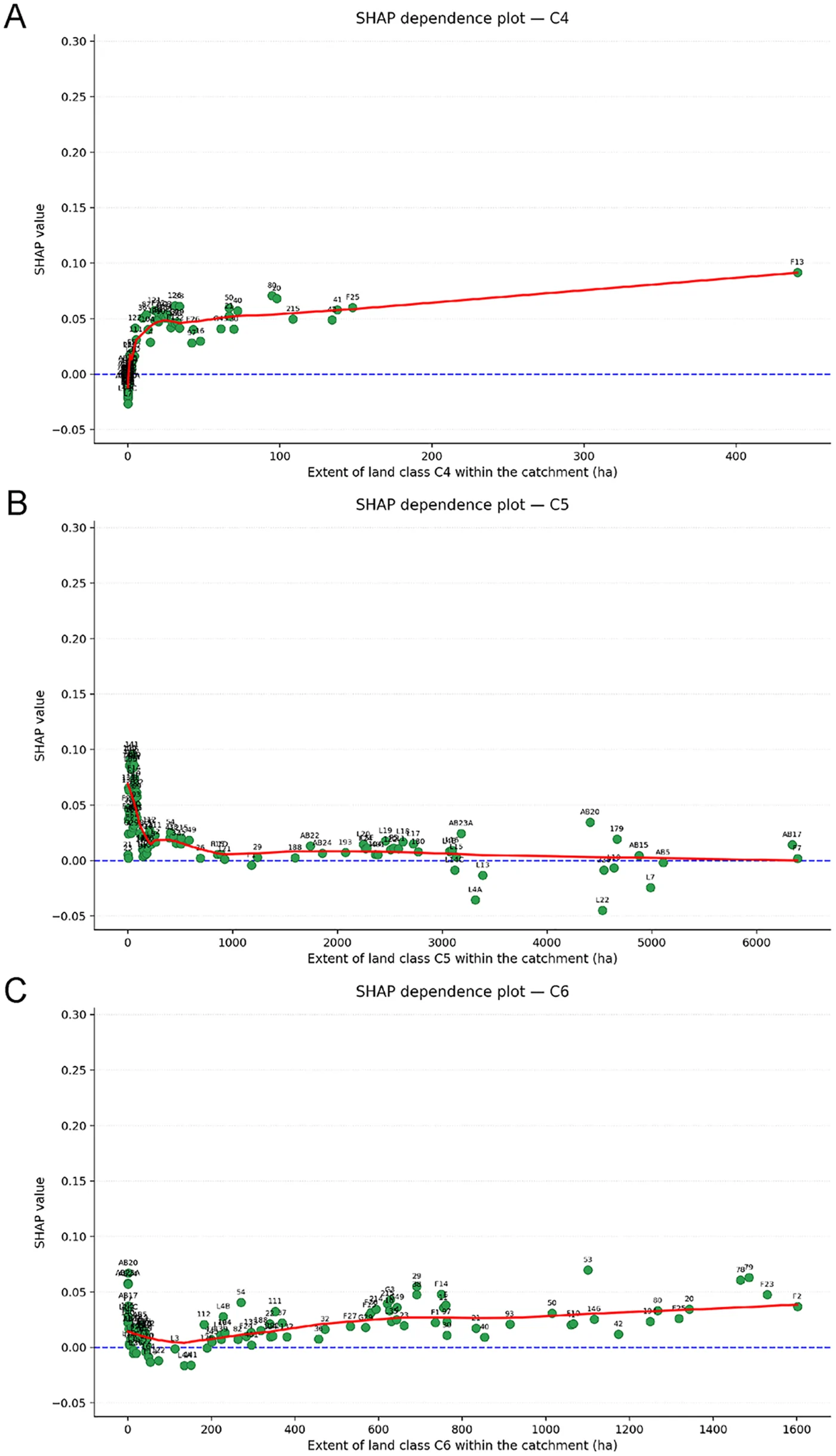
SHAP dependence plots for the soil classes in the Random Forest model: C4-6. SHAP values are expressed in units of predicted probability: they measure the shift in predicted probability of settlement presence attributable to each variable, relative to the model’s mean prediction. The dashed blue line marks SHAP = 0, and the red line shows the LOESS trend (frac = 0.5).

Whereas the ranking above summarises each predictor in a single value, the dependence plots (Fig 7–10) resolve its behaviour across the range of the variable (S5 Table). In this way, it becomes possible to observe how the predictive contribution changes (positively, > 0, or negatively, < 0) as the value of the feature varies. A LOESS curve (frac = 0.5) [43] has been added to each plot in order to estimate the overall trend. This allows the directional contribution of predictors (whether monotonic, threshold-like or non-linear) to be identified, and highlights which variables retain robust predictive behaviour through time.

**Altitude** (Fig 7A) discriminates settlements from pseudo-absences at both ends of its range, with an extensive interval of indifference between them. The lowest elevations form a compact and uniformly favourable group: all fourteen settlements below 10 m a.s.l. return positive contributions, from +0.013 to +0.076 (mean +0.050). The first negative value occurs at 10 m, and from there to site F13, at 113 m, the variable carries essentially no discriminating power: contributions alternate in sign and none exceeds ±0.022 in either direction. From the next settlement upwards (site 5, at 175 m) all contributions are positive, and the effect strengthens markedly at a well-defined point. From F23 (292 m) the values jump to a substantially higher level and remain there: the settlements between 292 and 415 m (site 97) are all positive, and include the two highest individual values of the plot, site 38 at 357 m and site 29 at 293 m (both ≈0.125). Above 450 m the contributions remain positive without exception but fall back to a lower plateau, with the highest settlement of the dataset (F25, 938 m) contributing +0.034. Altitude therefore operates through two distinct favourable settings rather than along a single gradient, immediate coastal lowlands on the one hand, intermediate uplands on the other, separated by a broad intermediate zone in which elevation is uninformative. The two are of unequal strength: the settlements between 292 and 415 m return contributions averaging +0.082, some three-fifths higher than the fourteen coastal settlements below 10 m (+0.050), and include the largest individual values recorded for this predictor. In the latter case the positive contribution may be associated with strategic visibility and defensibility.

The dependence plot for **sea** (Fig 7B) reveals a tripartite structure. The nine settlements lying within one minute’s walk of the shoreline form a tight cluster with the highest values in the plot (0.059 to 0.136). Four further sites between 6.7 and 12.3 minutes retain clearly positive but much reduced contributions (0.015 to 0.055). From about 20 minutes the sign of the effect reverses: across the whole interval between 20 and 200 minutes the contributions are predominantly negative, and between 49 and 131 minutes thirteen consecutive settlements return negative values without exception, though of small magnitude (−0.021 to −0.001). Beyond about 200 minutes the pattern reverses again and becomes strikingly consistent: sixty consecutive settlements, from site 111 (198 min) to site 50 (522 min), all return positive contributions of comparable magnitude (mean +0.029). The only negative values are those of the two most distant settlements in the dataset (sites 4 and 23, at 537 and 544 minutes). The variable therefore discriminates settlements from non-settlements at both extremes of its range while weakly penalising the intermediate zone, capturing two distinct environmental niches, immediate coastal locations and markedly inland positions, rather than a continuous gradient. The two niches are, however, of very unequal weight. The coastal niche is small but sharply defined: nine settlements, all within a single minute of the shoreline, returning contributions averaging +0.108. The inland niche is far broader but much shallower, comprising sixty settlements spread over more than five hours of walking time and contributing an average of +0.029 each. The intervening penalty is weaker still: across the twenty-five settlements between the two niches the mean contribution is −0.005, and six of them are in fact positive, so that the variable is better described as identifying a strong coastal attractor set against a diffuse inland background than as opposing two equivalent alternatives.

The dependence plot for **water bodies** (Fig 7C) resolves into four groups, the first two of which overlap in distance and differ in the strength of their contribution. The thirteen strongest contributions of the plot (0.050 to 0.172, mean 0.098) belong without exception to settlements lying within twelve minutes’ walk of a lake or lagoon, and include the highest single value recorded (site 29, 0.172). Below site 215 (11.8 min, 0.050) no further settlement of this distance band reaches that level: proximity was therefore not always sufficient, since F1 (9.5 min) and site 23 (10.2 min) lie within the same band yet contribute only about 0.030, a value matched by settlements more than a hundred minutes away (AB5 at 111 min, site 111 at 156 min). A second, much weaker group occupies the same range of distances: of the twenty-one settlements between F1 (9.5 min) and L10 (32.1 min), the nineteen that do not belong to the strong cluster are all positive but some six times lower (0.002 to 0.030, mean 0.015). From L22 (32.2 min) the sign reverses: across the interval to AB20 (76.8 min) twenty-five of twenty-nine settlements return negative values (mean −0.006), the four exceptions all falling in the first half of the range and none exceeding 0.007; beyond site 53 (44.9 min) the negative run is unbroken. From L16 (77.6 min) onwards the pattern dissolves into scatter, with twenty-nine of thirty-nine sites positive and contributions distributed on both sides of zero (mean 0.010), indicating the absence of any consistent directional effect at these distances.

The dependence plot for **rivers** (Fig 8A) shows a short-range attraction followed by indifference. The forty-eight settlements lying within 6.06 minutes’ walk of a watercourse form an unbroken sequence of positive contributions, with the effect decaying rapidly across this interval: mean values fall from 0.048 within the first two minutes to 0.026 between two and four, and 0.016 between four and six. The decline is therefore steep rather than gradual, indicating that the advantage conferred by river proximity was concentrated in the immediate riparian corridor and dissipated within a few hundred metres of it. The strongest contributions are those of AB24 and AB15, both located directly on a watercourse (0.109 and 0.096). Beyond around six minutes (6.06) the variable ceases to be informative. Across the remaining settlements the mean contribution is 0.005, values scatter on both sides of zero (fourteen of them negative, though only two below −0.02), and distance shows no detectable relationship with the contribution. Rivers therefore behave as a purely local attractor: once the riparian corridor is left behind, proximity to rivers neither favours nor disfavours settlement.

The dependence plot for **springs** (Fig 8B) displays the most regular structure of the whole set. The fifty-eight settlements lying within eighteen minutes’ walk of a spring form an unbroken sequence of positive contributions, without a single exception, and within this sequence the effect declines linearly with distance. Regressing the SHAP values of these fifty-eight settlements on their walking distance from the nearest spring yields a strong negative correlation (Pearson’s r = −0.80; p < 10^−^¹³), with the linear model accounting for 64% of the variance in the contributions (R² = 0.64). Mean contributions fall by a factor of eight across the interval, from 0.072 in the first three minutes to 0.009 between fifteen and eighteen, with the highest single value returned by site 112 (0.121). The fitted line reaches zero at 18.4 minutes, which is precisely where the observed sign change occurs: from L3 (18.2 min) nineteen consecutive settlements, up to site 214 (36.0 min), all return negative values without exception, and the trough is comparatively deep (−0.049 to −0.004, mean −0.021). Beyond thirty-seven minutes the pattern becomes mixed, with a slight prevalence of positive values, with fifteen of twenty-three settlements above zero (mean 0.010), before the single most distant site in the dataset (164, at 125 min) returns −0.030.

Springs thus emerge as a highly localised factor of attraction operating through a genuine reversal rather than a simple decay: their contribution diminishes steadily and predictably with walking time, turns unfavourable in an intermediate belt between roughly eighteen and thirty-six minutes where they were too distant to be exploited yet still part of the immediate landscape, and becomes largely indifferent beyond that range.

The dependence plot for **topographic settings** (Fig 8C) exhibits by far the strongest and most orderly structure in the model, and the only one producing a complete separation between groups of cases. All thirty-four settlements occupying plateaux, isolated peaks or ridges return positive SHAP values, and all sixty-six occupying hills, plains or slopes return negative ones, without a single exception. The separation is not merely one of sign but of scale: the lowest value in the first group (0.119) and the highest in the second (−0.009) are separated by a gap of 0.128. Plateaux yield the highest contributions (mean 0.246, reaching 0.306 at site 122, the largest single value recorded anywhere in the model), followed by isolated peaks (mean 0.196) and ridges (mean 0.176). Among the remaining classes the contributions are consistently negative but roughly an order of magnitude smaller: slopes are the least favourable (−0.055), followed by plains (−0.028) and hills (−0.020).

The dependence plot for **C1** (Fig 9A) resolves into three stages. Where this highly fertile land class is entirely absent from the catchment the contribution is unambiguously unfavourable: all thirteen settlements with no C1 return negative values, tightly clustered between −0.044 (site L5E) and −0.022 (site AB15), with a mean of −0.035. From the first non-zero values up to site 97 (214 ha) the contributions fluctuate around zero, with positive and negative values alternating and only three sites exceeding ±0.02. Site 97 is the last negative value of the plot: from that point onwards fifty-three consecutive settlements return positive contributions without exception. Computed by intervals of extent, the mean contribution rises from +0.010 between 214 and 300 hectares (n = 5) to +0.021 between 300 and 400 (n = 11) and +0.041 between 400 and 500 (n = 2), reaching +0.066 above five hundred hectares (n = 35). Above that extent the values reach a level around which they then remain, with the thirty-five settlements in this range averaging +0.066 (from +0.029 to +0.096) and showing no further increase.

The behaviour of C1 is therefore consistent with a threshold rather than a cumulative logic. Limited patches of high-quality soil were not sufficient to favour settlement, and their complete absence was actively unfavourable, but once a few hundred hectares were available the advantage was fully realised and further extent brought no additional benefit. This is consistent with a catchment-scale interpretation: beyond the surface a community could realistically cultivate, additional fertile land ceased to be a differentiating factor.

The dependence plot for **C2** (Fig 9B) displays a pronounced U-shaped structure, with strong positive contributions at both extremes of the range and a negative interval between them. Where this land class is nearly absent the effect is at its strongest: the eighteen settlements with less than twelve hectares of C2 all return positive values, ranging from site L22 (0.036) to site AB5 (0.117) (mean 0.083). The effect then collapses abruptly, from L15 (11.6 ha, 0.065) to F5 (30.5 ha, 0.001), within twenty hectares and two consecutive sites. Across the interval that follows the contributions hover around zero without a stable sign, remaining weakly positive up to about two hundred hectares (seven settlements, mean 0.013) and alternating thereafter. A consistently negative stretch is confined to the middle of the range: from L7 (912 ha) to site 29 (2806 ha) twenty-one consecutive settlements return negative values without exception. Beyond 2806 hectares the negative signal dissolves, and from about 3500 hectares the contributions become positive again, at first modestly (nineteen of twenty-one sites up to 4800 ha, mean 0.013) and then strongly: all fifteen settlements with more than 4800 hectares of C2 return positive values, averaging 0.074 and reaching 0.092.

The two positive extremes are of comparable magnitude, so the variable does not express a fertility gradient but distinguishes two contrasting catchment configurations, those in which C2 is effectively absent and those in which it is overwhelmingly dominant, from the intermediate condition in which it is present in moderate quantity. The negative interval is an order of magnitude weaker than either positive tail, so the pattern is better described as two distinct positive signals separated by indifference than as an active penalty at intermediate extents. Given that the total catchment area is fixed, a near-total absence of C2 necessarily implies the predominance of some other land class, and the two positive tails may therefore reflect the model’s response to strongly characterised catchments of either kind rather than to the properties of C2 itself.

The dependence plot for **C3** (Fig 9C) displays the clearest structure among the soil classes, and one of the clearest in the model. The seventy-nine settlements with less than 830 hectares of this land class form an unbroken sequence of positive contributions, without a single exception, spanning almost the entire lower two-thirds of the range. Within this sequence the effect is strongest at the smallest extents (the eleven settlements below sixty hectares average 0.110, and the maximum of the plot is reached at eighteen hectares with L3: 0.147), and then declines to a stable plateau, with mean values of 0.052 (60–200 ha), 0.064 (200–450 ha) and 0.049 (450–830 ha) across the successive intervals up to 830 hectares. The reversal is abrupt. The last positive value (F10, 826 ha, 0.018) is followed immediately by the first negative one (site 82, 879 ha, −0.015), and across the whole interval from 830 to 2450 hectares eighteen of nineteen settlements return negative contributions (mean −0.024). The minimum of the plot is reached at 1137 hectares (site 166, −0.055). These negative values beyond roughly 830 hectares should not be read as a genuine disadvantage conferred by the presence of moderately fertile soils. Rather, they indicate that once the available surface exceeded the scale of land a community could realistically exploit, additional hectares ceased to be informative: the difference between ten and a hundred hectares of workable soil may have been decisive, whereas that between one thousand and two thousand was of no practical consequence. At such magnitudes the SHAP values no longer capture the intrinsic role of the variable but reflect its covariation with other components of the catchment.

The contribution of **C4** is negligible, as it is distributed in only minimal quantities within the study area (Fig 10A).

The dependence plot for **C5** (Fig 10B) shows a predominantly positive influence across almost the entire range, with a well-defined maximum at small to moderate extents. Ninety-one of the hundred settlements return positive contributions, and the settlements with less than about 1180 hectares of this land class form an unbroken positive sequence without exception, up to site 121 (922 ha), the last before the first negative value (F1, 1178 ha). The effect peaks between roughly fifteen and ninety hectares, where all twenty-eight settlements return values averaging 0.063 and reaching 0.095 (site 141, at 39 ha). Below fifteen hectares the contribution is positive but lower (mean 0.037), and above ninety hectares it falls to a low but persistently positive plateau, averaging 0.015 between 90 and 950 hectares and 0.009 between 950 and 3100. Only beyond roughly 3100 hectares do negative values become frequent (eight of the fifteen settlements in this range), with the minimum of the plot at 4528 hectares (L22, −0.045).

For **C6** (Fig 10C), it is important to note first that the scale of the surfaces involved is very limited, representing only minimal portions of the landscape. Within this narrow range, the dependence plot for C6 shows an unexpectedly structured and predominantly positive pattern. Eighty-nine of the hundred settlements return positive contributions. Very small extents produce a first positive signal: the fourteen settlements with less than twelve hectares of this land class are all positive, averaging 0.029 and reaching 0.067 (AB20). Between roughly twelve and two hundred hectares the signal weakens and becomes irregular, with eleven of twenty-nine settlements returning small negative values and the minimum of the plot at 135 hectares (L4A, −0.016). Beyond two hundred hectares, however, the contribution becomes systematically positive and grows with extent: the fifty-seven settlements in this range are positive without a single exception, their values correlate significantly with the surface available (r = 0.55, p < 10^−^⁴), and the means rise from 0.022 between 200 and 700 hectares to 0.032 above 700, with the maximum of the plot at 1100 hectares (site 53, 0.070).

C6 therefore does not behave as a negligible predictor. Since this class comprises land unsuitable for cultivation but capable of supporting woodland, its positive contribution at substantial extents is perhaps interpretable in terms of access to timber, fuel, pasture and wild resources, which were as necessary to the viability of a settlement as arable land.

Taken together, the soil dependence plots do not describe a simple fertility gradient in which better land classes attract settlement and poorer ones repel it. Each class contributes through a distinct and interpretable mechanism, and the resulting picture is one of balanced catchment composition rather than maximisation of any single soil type. The two classes suited to staple cultivation behave as complementary necessities: C1, the prime arable land, probably operates through a threshold: its complete absence actively penalises a location, while its advantage is fully realised at around five hundred hectares and does not grow further, consistent with the surface a community could actually work. C3, suitable for tree crops, is the most consistently favourable class of all, positive without exception up to roughly 830 hectares and strongest where its extent is smallest, but reversing sharply beyond that threshold in a clear pattern of diminishing returns. The poorer classes, far from being irrelevant, carry positive signals of comparable magnitude: C5, despite its severe limitations, contributes most strongly at moderate extents, perhaps because steep or stony ground is the natural counterpart of the prominent, defensible landforms favoured by settlement; and C6, unsuitable for agriculture but capable of supporting woodland, grows steadily more favourable with extent beyond two hundred hectares, pointing to the value of timber, fuel and wild resources as structural components of a viable catchment. C2 stands apart: only marginally less fertile than C1, it rewards the two extreme configurations, near-absence and overwhelming dominance, while remaining indifferent in between, a behaviour best understood compositionally, as the signature of strongly characterised catchments rather than of the agronomic properties of the class itself. What emerges may suggest that settlement locations were associated not with the greatest possible amount of fertile land, but with catchments combining a sufficient core of arable soil, a moderate presence of rough terrain, and an appreciable reserve of uncultivable land, a mixed portfolio in which each component, from cereals to woodland, had a bounded and complementary role.

### Normalized split frequencies by phase

**Fig 11:**
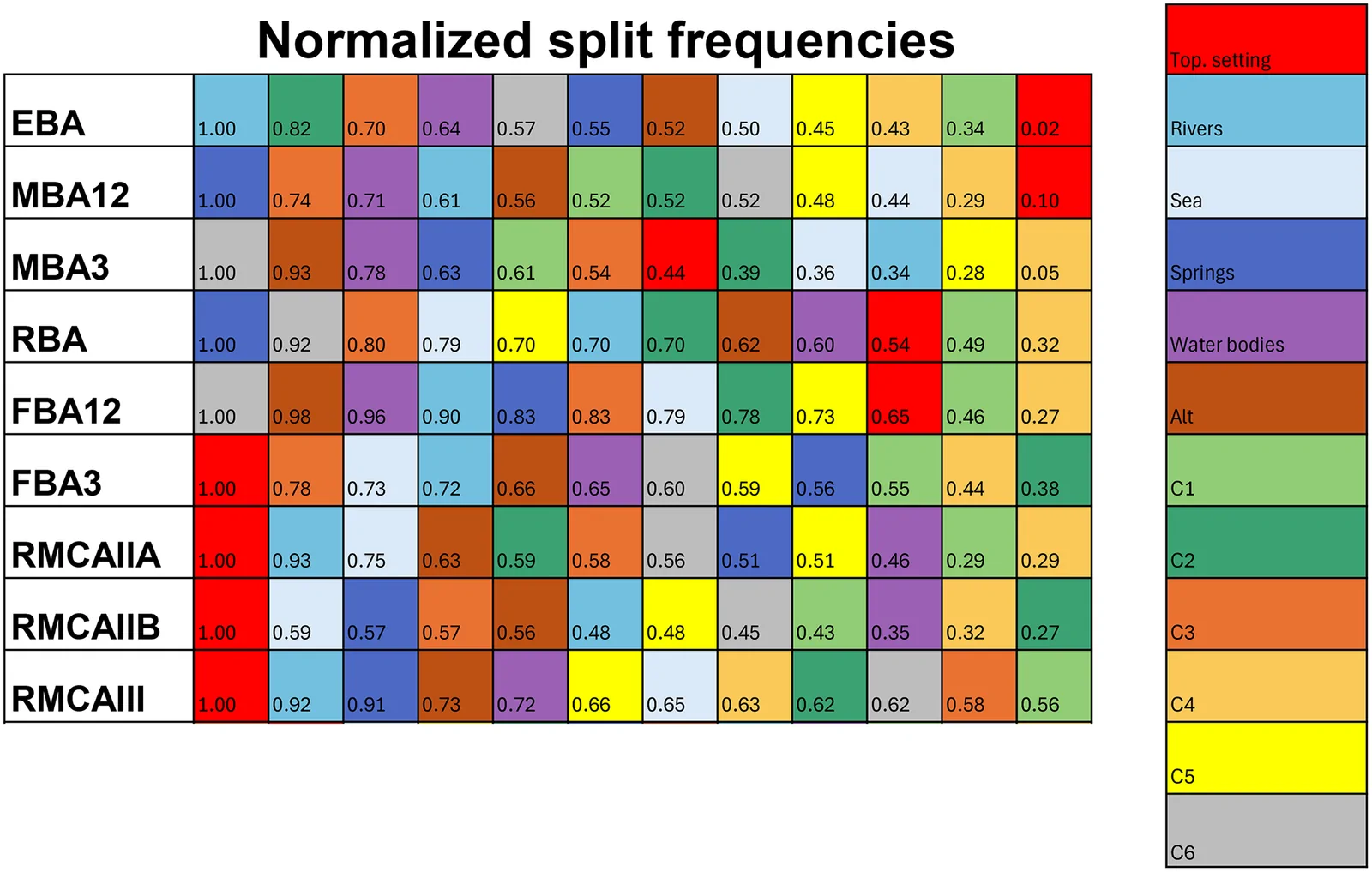
Importance of environmental variables across chronological phases. Variable importance based on normalized split frequencies in the Random Forest.

**Fig 12:**
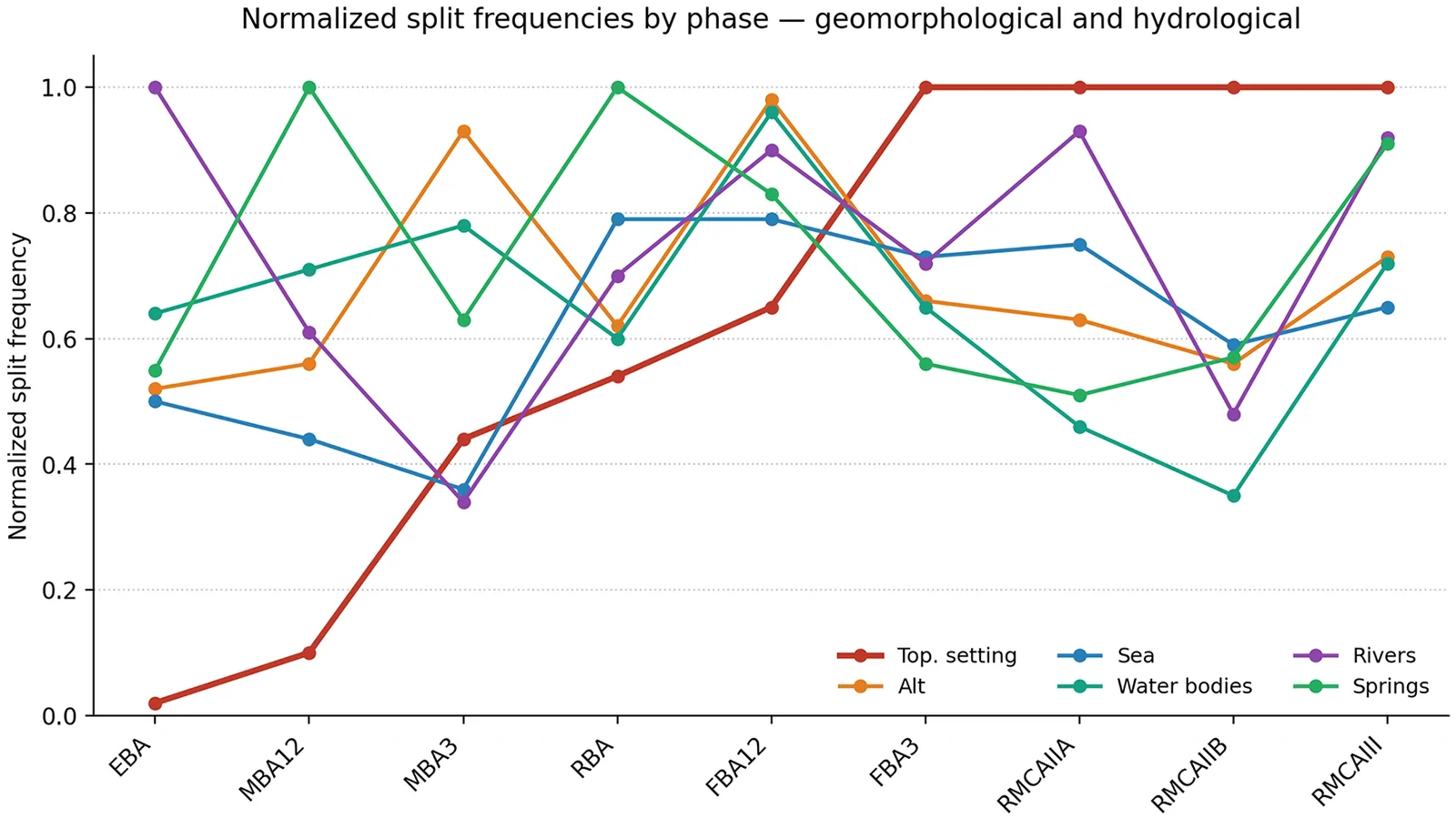
Trend of normalized split frequencies across the chronological phases, geomorphologic parameters.

**Fig 13:**
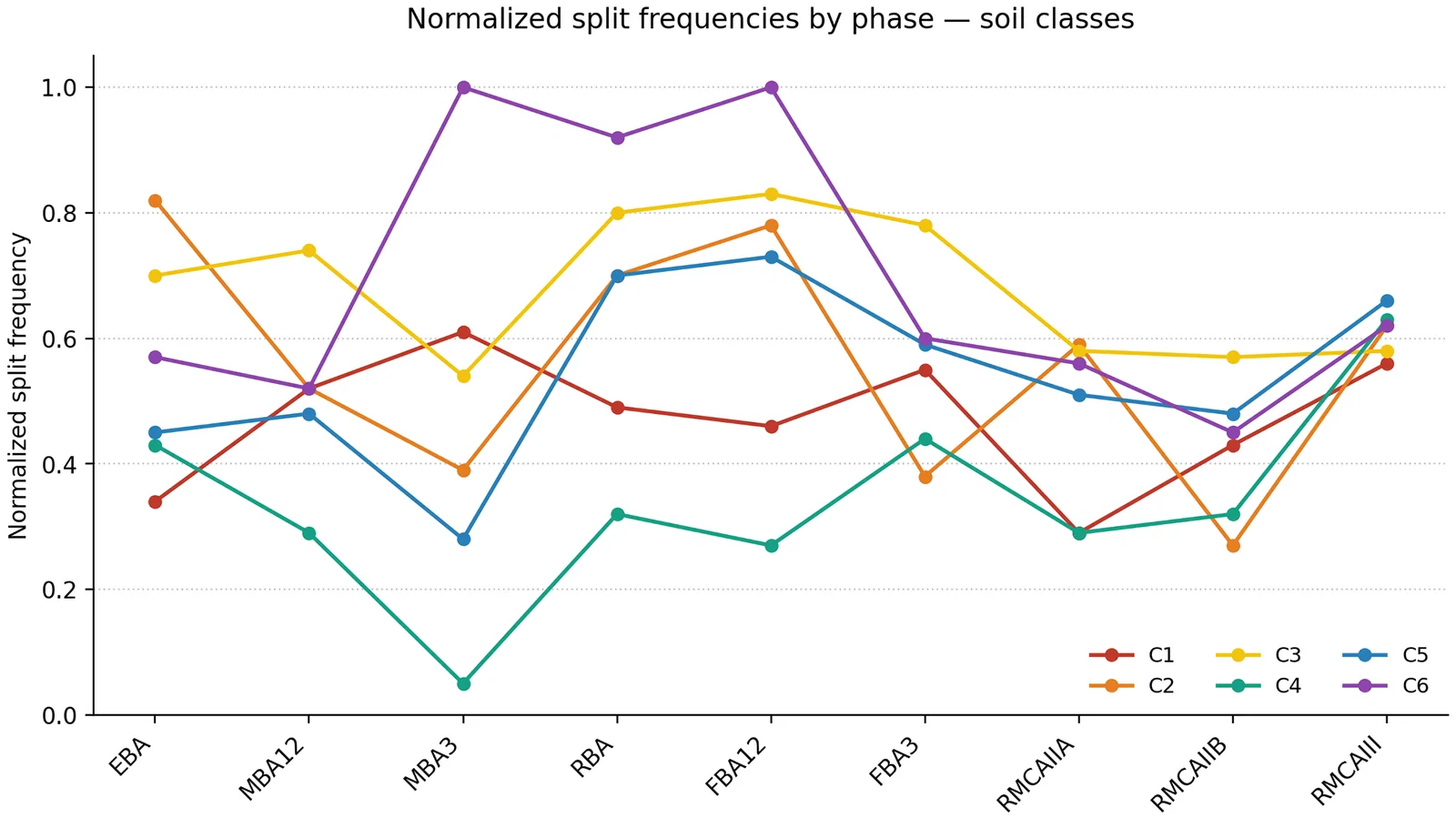
Trend of normalized split frequencies across the chronological phases, soil parameters.

Normalized split frequencies show a clear shift in the predictors used by the model to discriminate archaeological sites from non-sites across the chronological sequence (Figs 11–13 and S10 Table). In the EBA–MBA12, hydrological variables dominate: Rivers reaches very high values (1.00 and 0.61), and both Springs and Water bodies exhibit strong contributions (up to 1.00 and 0.71), whereas topographic setting remains negligible (0.02–0.10). From MBA3 onwards, topographic structure becomes increasingly informative. Its trajectory is the most regular in the entire matrix, rising monotonically without a single reversal (0.02, 0.10, 0.44, 0.54, 0.65) before attaining maximum importance in all phases from FBA3 to RMCAIII (1.00), where it remains saturated. Altitude contributes consistently across the entire sequence (0.52–0.98), peaking in FBA12.

Proximity to the coast (Sea) displays moderate values in EBA-MBA3 (0.36–0.50) but intensifies from RBA onward, reaching 0.79 in both RBA and FBA12 and remaining high in RMCA phases (0.59–0.75). Crucially, the hydrological predictors do not decline as topographic setting rises: Springs returns to 1.00 in RBA and to 0.91 in RMCAIII, and Rivers reaches 0.93 in RMCAIIA and 0.92 in RMCAIII, values comparable to those of the earliest phases. The sequence therefore does not document the replacement of one set of predictors by another, but the emergence of topographic setting as a saturating additional criterion alongside hydrological variables that remain structurally informative throughout.

Soil-fertility classes (C1–C6) show heterogeneous contributions. C1 maintains moderate values across the whole sequence (0.29–0.61), whereas C2 fluctuates considerably (0.27–0.82), with its highest value in the EBA and its lowest in RMCAIIB. C6 becomes highly informative in specific intervals (1.00 in MBA3 and FBA12), and C4 remains generally low throughout, with the single exception of RMCAIII, where it rises to its maximum (0.63).

Together, these patterns document a progressive addition rather than a substitution of criteria: an early landscape discriminated on hydrological grounds is joined, from MBA3 onwards, by a topographic dimension that becomes and remains the single most informative structural criterion of the model.

### Phase-by-phase mean SHAP values

**Fig 14:**
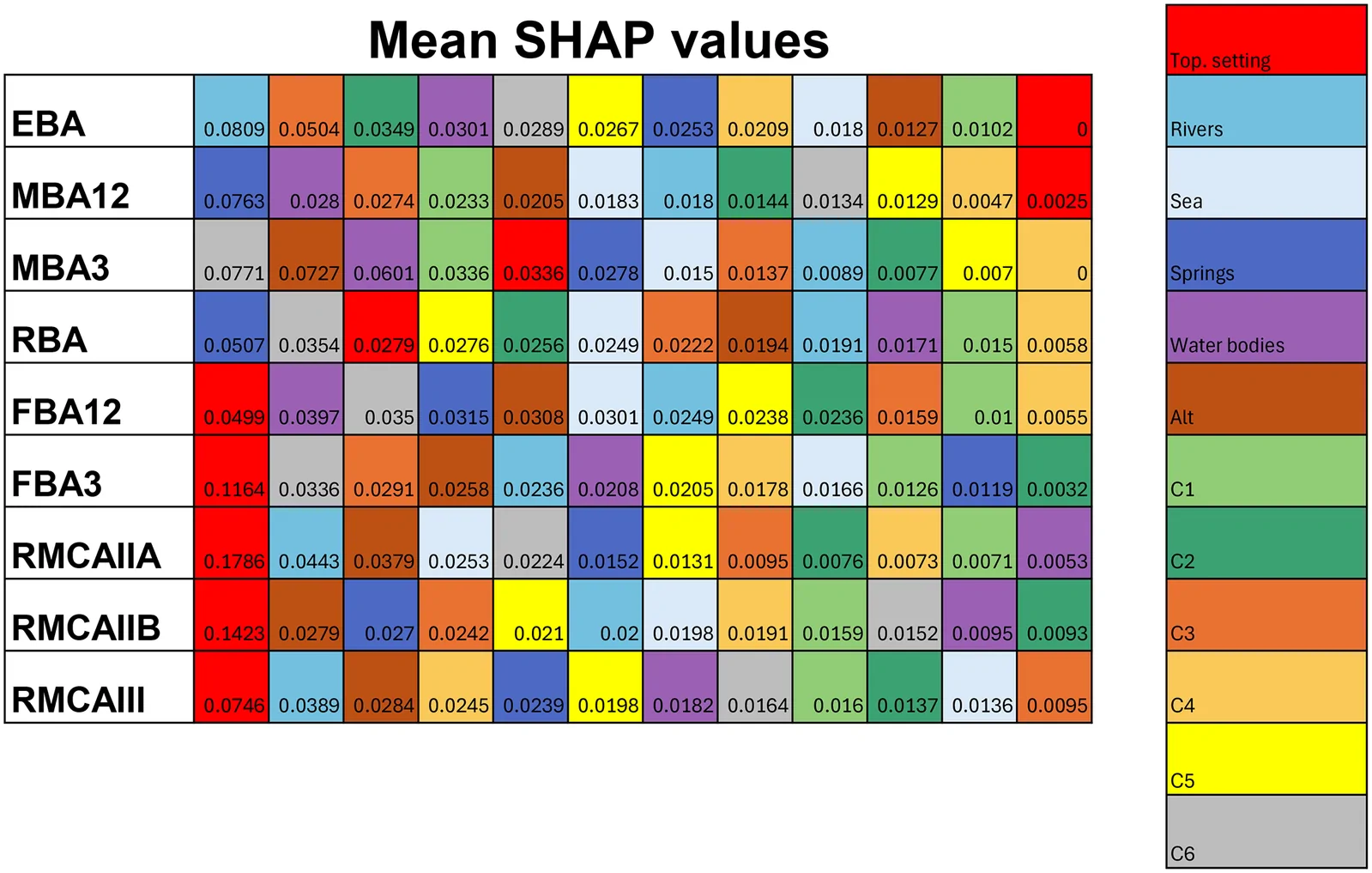
Phase by phase mean SHAP values for the real settlement positive outcome.

The mean SHAP values are analysed here phase by phase for each feature (Fig 14). The values refer only to those settlements whose existence was correctly predicted (S6 Table).

It must be emphasised that the number of sites available for each phase remains modest, ranging from nine in the EBA to thirty in RMCAIII (S6 Table). From the Iron Age onwards (RMCAIIA) the situation improves owing to the larger number of attestations, yet the overall sample remains restricted, which means that the patterns identified here should be regarded as exploratory signals rather than confirmatory evidence. The results are best interpreted as heuristic insights pointing to recurrent trends that require further testing, rather than as statistically robust demonstrations of settlement behaviour.

**Fig 15:**
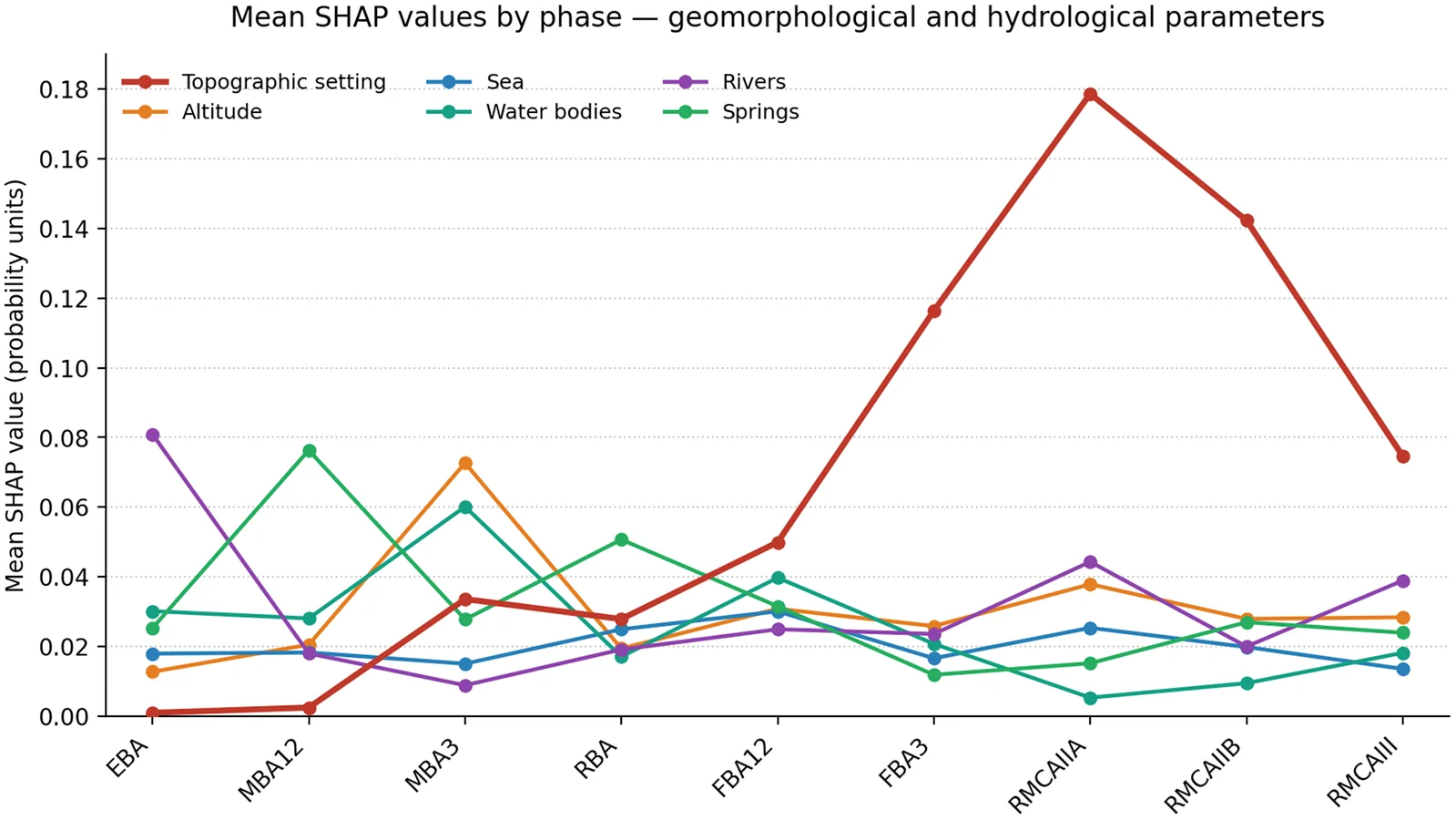
Trend of mean SHAP values for the real settlement positive outcome across the chronological phases, geomorphologic parameters.

**Fig 16:**
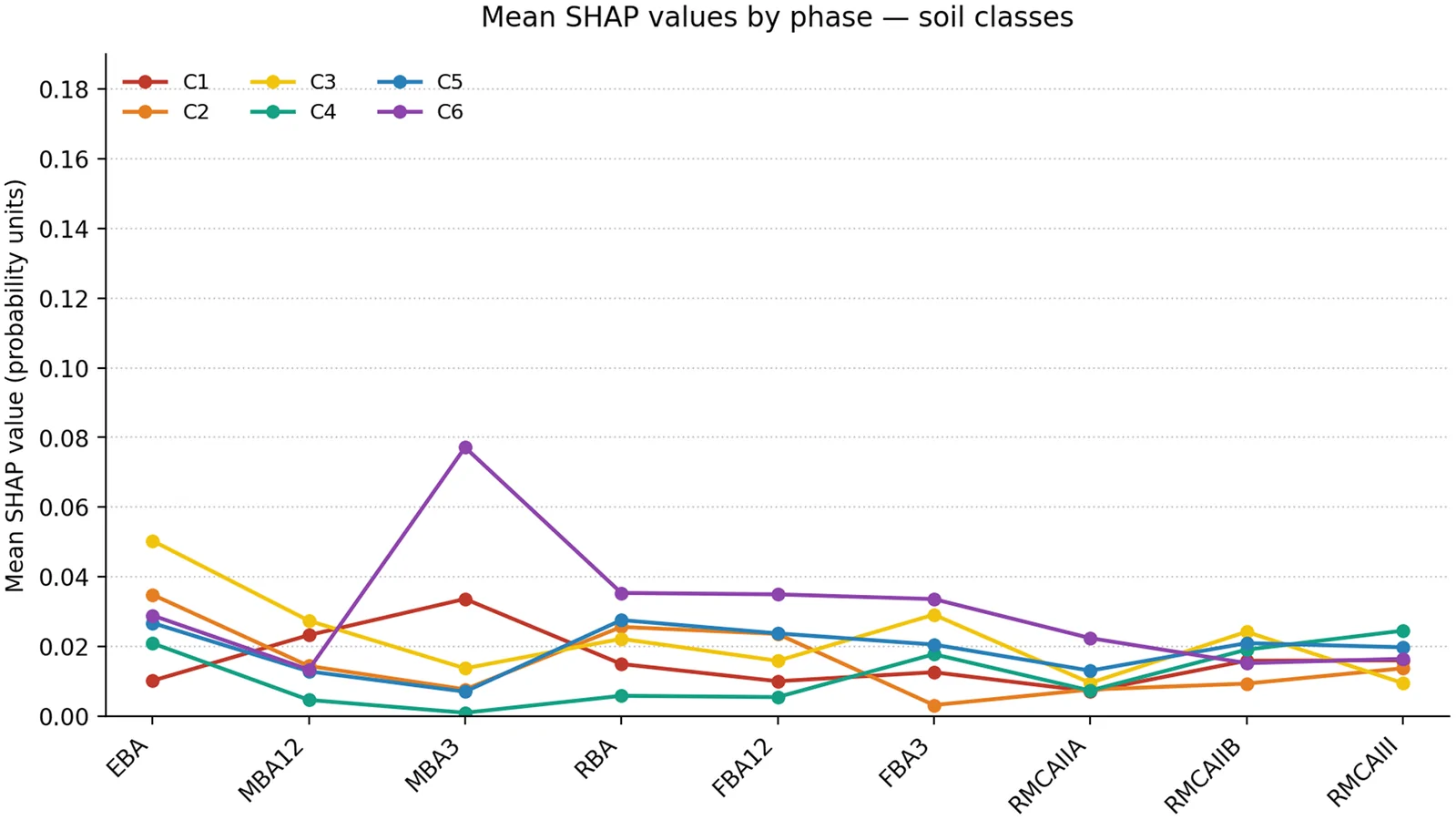
Trend of mean SHAP values for the real settlement positive outcome across the chronological phases, soil parameters.

Across the chronological sequence, the mean SHAP values reveal a progressive reconfiguration of the environmental factors that contribute to distinguishing archaeological sites from non-sites (Figs 15, 16). In the earliest phases the model draws predominantly on hydrological variables, although the specific source differs: in the EBA the leading predictor is proximity to rivers (0.081), followed by the soil classes C3 (0.050) and C2 (0.035), whereas in MBA12 springs provide by far the most substantive contribution (0.076). During both phases the influence of topographic setting is effectively nil (0.001 and 0.002) and altitude plays only a marginal role, indicating that local hydrological availability, and secondarily soil fertility, constituted the most informative dimension for separating sites from their surrounding landscapes. A first shift becomes apparent in MBA3, where topographic setting registers a measurable effect for the first time (0.033, an order of magnitude above the preceding phases), although it is not yet the leading predictor: the phase is characterised instead by the highest contributions of C6 (0.077) and altitude (0.073), together with water bodies (0.060). This configuration marks the onset of a move away from purely hydrological criteria towards the morphological properties of settlement locations, while the overall distribution of predictive weight remains diffuse. In the subsequent RBA phase the contributions of individual variables are generally low and evenly spread, with springs retaining a modest lead (0.050) and no predictor clearly dominating the discrimination process. From FBA12 onward the structure of model importance changes. Topographic setting becomes the leading variable (0.050), initially by a narrow margin over water bodies and C6, but its contribution then increases sharply in FBA3 (0.116) and peaks in RMCAIIA (0.179), where it exceeds the second-ranked predictor by a factor of four. It remains clearly dominant in RMCAIIB (0.142) before attenuating in RMCAIII (0.075), where it is still the highest-ranked variable but by a reduced margin. Throughout these later phases altitude provides a modest and stable secondary contribution (0.026–0.038), consistent with its coupling to topographic preferences.

Taken together, the phase-specific mean SHAP values confirm and refine the trends already highlighted by the normalized split frequencies, by identifying which predictors not only structure the model but also actively favour site occurrence. The earliest phases are characterised by the prominence of hydrological availability and, locally, soil fertility, whereas from FBA12 onward topographic setting emerges as the factor exerting the strongest and most persistent positive influence on site presence, with altitude providing a secondary reinforcement. Hydrological and pedological predictors retain small positive mean contributions throughout the sequence, and in some later phases rivers still rank second (0.044 in RMCAIIA; 0.039 in RMCAIII). What changes across the sequence is the magnitude of their effect relative to topographic setting, which comes to overshadow all other predictors within an increasingly canalised settlement logic.

## Discussion

### Comparison with the statistical framework

The only study on *Latium vetus* that applies a statistical test (one-sample Kolmogorov-Smirnov goodness-of-fit test, two-tailed) to settlement distribution for these periods is Alessandri 2013, which thus provides the most appropriate framework within which to contextualise the Random Forest results. That study demonstrated that access to freshwater springs was a statistically significant determinant of settlement location throughout the protohistoric sequence of *Latium vetus* (and plausibly beyond). Specifically, the clustering of settlements within 12.2 minutes of a spring was found to be significant. This threshold is very close to the sign change observed at 18.2 minutes, at which the predictive contribution of springs shifts from positive to negative. That proximity to springs, and to potable water sources more broadly, served as a major driver, particularly in the earliest phases, is also supported by both the split-frequency analysis and the phase-resolved SHAP values. This concordance reinforces the interpretation of an interval around 15 minutes from water sources as a critical threshold for settlement siting.

Alessandri 2013 also identified proximity to the sea and altitude as statistically significant variables.

In the case of the sea, a clustering of settlements within 62.7 minutes was observed. The dependence plot presented here confirms that coastal proximity is a real determinant, but places the effect on a considerably tighter scale: the positive contribution is confined to the first twelve minutes of walking time, and is overwhelmingly concentrated in the nine settlements lying within a single minute of the shoreline. At 62.7 minutes the SHAP contributions are in fact weakly negative. The two results are not in contradiction, since they measure different things: the earlier analysis tested whether settlements are more clustered towards the coast than would be expected at random, whereas the SHAP values quantify how far each distance discriminates settlements from pseudo-absence points. The present analysis suggests that the earlier threshold captured the outer margin of a coastal tendency whose predictive strength is in reality restricted to immediate shoreline locations.

For altitude, the statistical analysis identified two significant clusters, below 33 m and between 281 and 381 m, and the dependence plot confirms both, though with a lower band narrower than the earlier threshold suggested. The predictive effect of low elevation proves to be concentrated within the first ten metres rather than extending to 33 m, but the two analyses agree in isolating the immediate coastal lowlands as a distinct and favourable setting.

The upper band also corresponds closely, though the two intervals are offset. The statistical clustering was identified between 281 and 381 m, whereas the dependence plot places the segment of strongest SHAP contributions between 292 and 415 m. The split frequencies likewise indicate that elevation contributes a consistent amount of information across all phases (0.52–0.98).

These parallels should not be viewed as formal validation, yet they amount to more than incidental correspondence. The recurrence of comparable thresholds across independent analytical frameworks indicates a coherent signal that plausibly reflects underlying settlement-selection processes. Although additional analyses on expanded datasets will be required to test the generality of these thresholds, the present convergence already provides a multi-method basis for their interpretation.

### Interpretation of the results from a (proto)historic perspective

Taken together, all these observations point to a systematic, long-term shift in the dimensions along which settlement location differs from non-settlement areas (all metrics in S4 Table).

#### EBA (2100−1700 BCE): Dispersed settlement driven by hydrological features.

**Fig 17:**
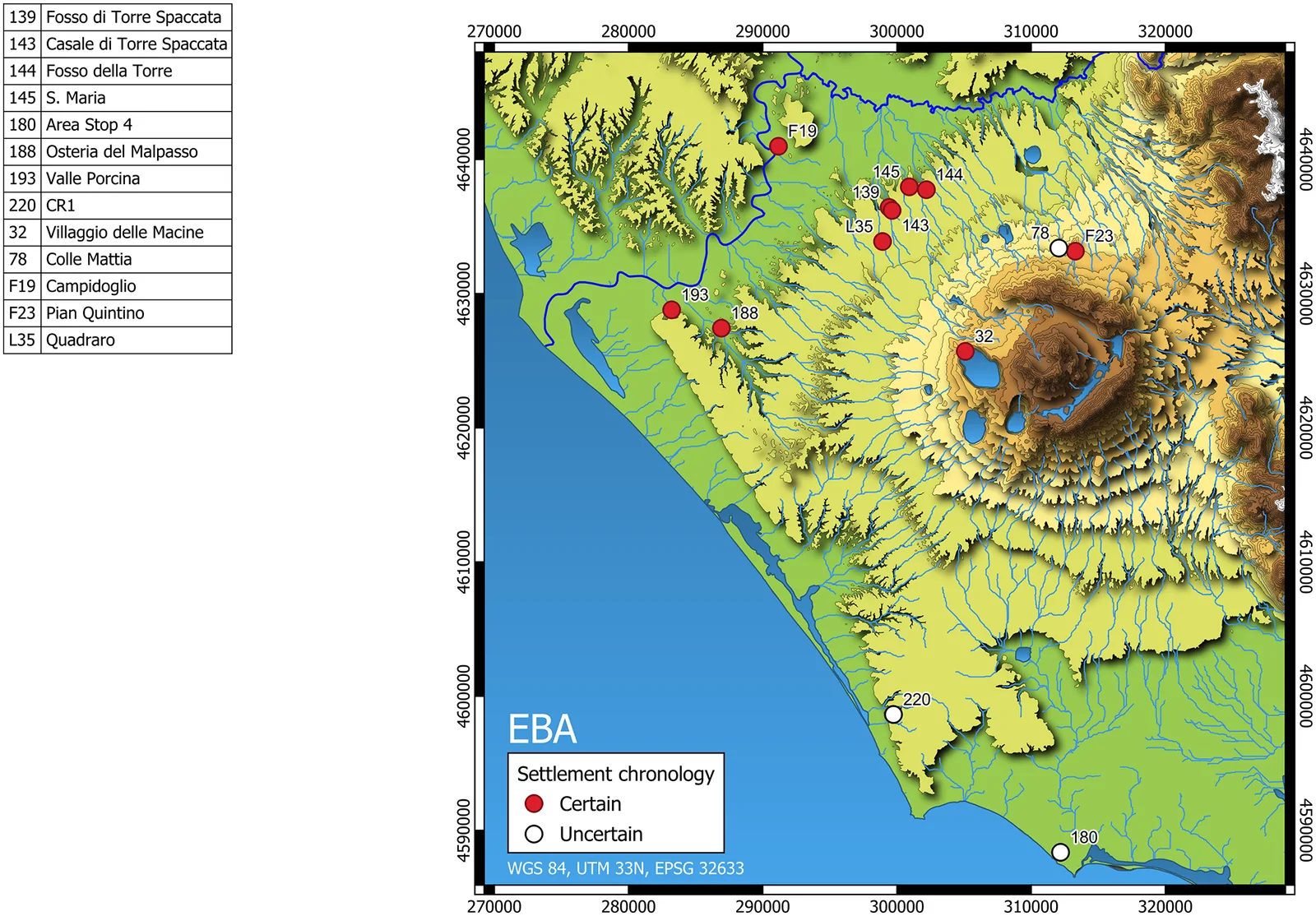
Phase map with Early Bronze Age settlements. Site locations, reconstructed shoreline and lakes/lagoons as determined by Alessandri (2013). Background, elaborated by the author from TinItaly dataset (Tarquini S., I. Isola, M. Favalli, A. Battistini, G. Dotta, 2023. TINITALY, a digital elevation model of Italy with a 10 meters cell size (Version 1.1). Istituto Nazionale di Geofisica e Vulcanologia (INGV). https://doi.org/10.13127/tinitaly/1.1). The dataset is published under a CC BY 4.0 license (downloaded from https://tinitaly.pi.ingv.it/Download_Area1_1.html). Map created with QGIS 3.40, (www.qgis.org).

Both the normalized split frequencies and the mean SHAP values for the earliest phase (Fig 17) indicate, on the one hand, that the site’s morphological characteristics have a negligible impact (topographic setting returns the lowest values of the entire sequence), and on the other, that the model relies primarily on hydrological predictors, reflecting a landscape in which access to water is the most informative feature distinguishing sites from their surroundings. Access to rivers and water bodies, beyond providing potable water, may also have reflected the need to settle along major and easily navigable communication corridors, valleys often serving as preferential movement routes, or the opportunity to exploit specific ecological niches such as freshwater fishing or the hunting of migratory waterfowl. An illustrative case of the latter is the site of Villaggio delle Macine (EBA-MBA12), where archaeozoological analyses indicate a strong incidence of hunting activity, with the presence of deer and roe deer, together with ichthyofauna and turtles [44]. It is worth also noting A. Guidi’s hypothesis suggesting that settlement proximity to perennial water sources may have been influenced by a particularly arid climatic phase, as evidenced by palynological analyses from the surrounding regions [45]. Alongside the hydrological signal, soil quality occupies a prominent place: classes C2 and C3 rank second and third on both measures. The combination is coherent from a subsistence perspective, since C2 offers land suitable for all forms of cultivation with only minor limitations, while C3 supports tree crops, so that the two classes describe complementary rather than equivalent agricultural options. That both should emerge in the earliest phase, alongside proximity to watercourses and in the absence of any topographic criterion, points to a settlement logic organised around the productive potential of the immediate catchment rather than around the defensibility of the site itself.

#### MBA12 (1700−1400 BCE): Territorial expansion.

The MBA12 model is the one case in the sequence, together with the RBA, in which the classification does not exceed chance expectation on either metric. Its cross-validated recall coincides exactly with the null expectation, and the gap between training and validated performance is among the widest observed. The environmental variables considered here therefore do not distinguish MBA12 settlements from pseudo-absence points, and neither the split frequencies nor the mean SHAP values for this phase can be interpreted as evidence of environmental preference: they describe how the model behaves, not how settlements were chosen.

**Fig 18:**
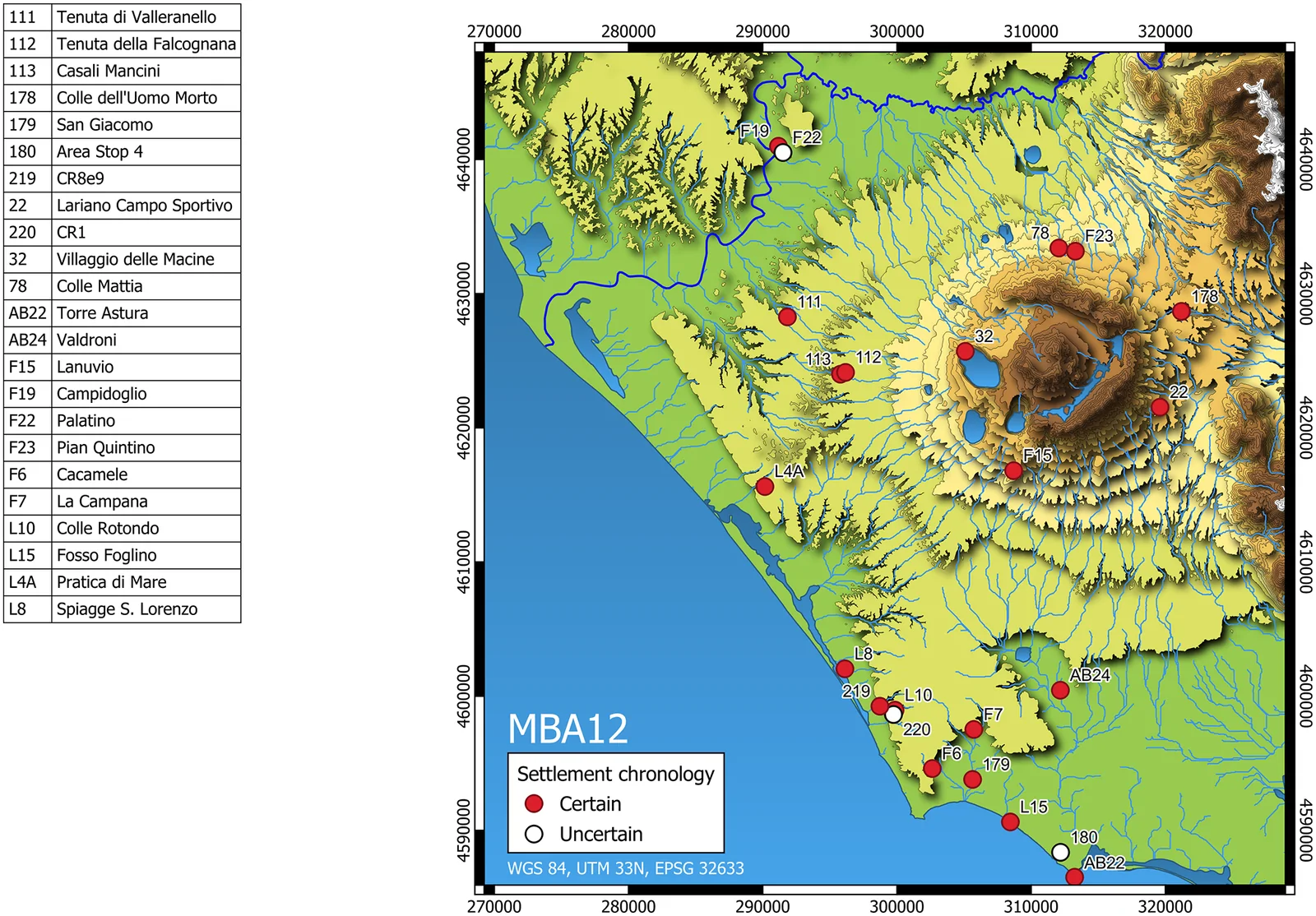
Phase map with Middle Bronze Age settlements, subphases 1 and 2. Site locations, reconstructed shoreline and lakes/lagoons as determined by Alessandri (2013). Background, elaborated by the author from TinItaly dataset (Tarquini S., I. Isola, M. Favalli, A. Battistini, G. Dotta, 2023. TINITALY, a digital elevation model of Italy with a 10 meters cell size (Version 1.1). Istituto Nazionale di Geofisica e Vulcanologia (INGV). https://doi.org/10.13127/tinitaly/1.1). The dataset is published under a CC BY 4.0 license (downloaded from https://tinitaly.pi.ingv.it/Download_Area1_1.html). Map created with QGIS 3.40, (www.qgis.org).

The absence of a signal is, however, archaeologically informative. MBA12 (Fig 18) is on independent grounds an expansive phase, in which the number of known settlements rises sharply (almost double) and multiple territorial domains appear to have been explored simultaneously [3]. A phase of this kind is precisely one in which settlements would not be expected to share a consistent environmental profile: if communities were testing a range of ecological niches rather than converging on a preferred one, no single combination of variables could separate their locations from the surrounding landscape. The failure of the model is thus the expected outcome of a settlement system not yet organised around a dominant environmental criterion, and it stands as the counterpart of the increasingly structured patterns that emerge from MBA3 onwards.

#### MBA3 (1400−1300 BCE): The beginning of a structured landscape.

**Fig 19:**
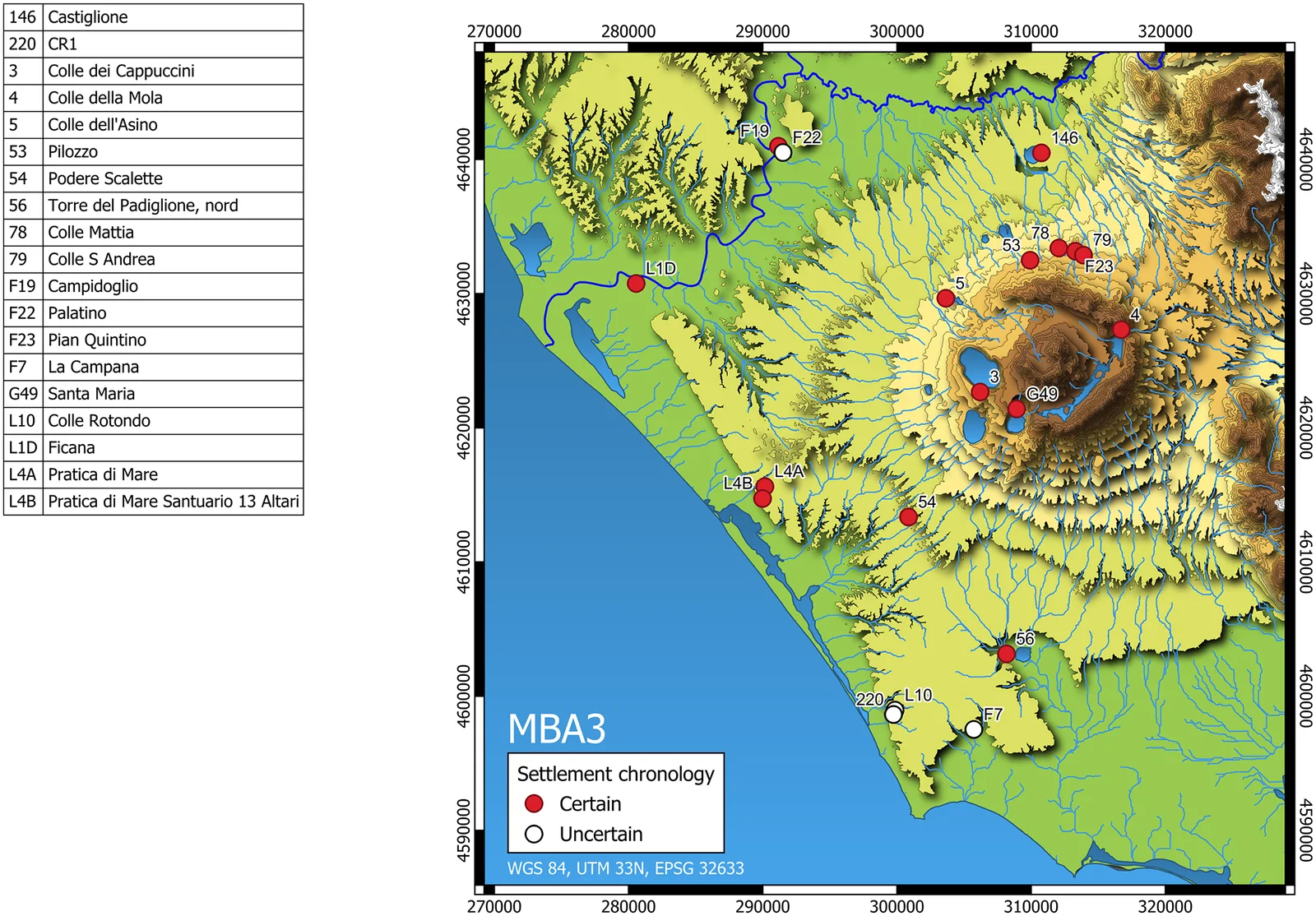
Phase map with Middle Bronze Age settlements, subphase 3. Site locations, reconstructed shoreline and lakes/lagoons as determined by Alessandri (2013). Background, elaborated by the author from TinItaly dataset (Tarquini S., I. Isola, M. Favalli, A. Battistini, G. Dotta, 2023. TINITALY, a digital elevation model of Italy with a 10 meters cell size (Version 1.1). Istituto Nazionale di Geofisica e Vulcanologia (INGV). https://doi.org/10.13127/tinitaly/1.1). The dataset is published under a CC BY 4.0 license (downloaded from https://tinitaly.pi.ingv.it/Download_Area1_1.html). Map created with QGIS 3.40, (www.qgis.org).

The transition to MBA3 (Fig 19) marks a change of regime. The model classifies significantly better than chance on accuracy, and the gap between training and validated performance narrows appreciably with respect to the preceding phase; the signal is not equally strong on recall, so the results are best treated as indicative rather than firmly established, but they stand in clear contrast to the complete absence of discrimination found in MBA12. Topographic setting, inert in both preceding phases, registers a measurable contribution for the first time: its normalized split frequency rises more than fourfold, and its mean SHAP value increases by an order of magnitude. It is not yet the leading predictor, altitude, water bodies and soil class C6 return higher values on both measures, but the phase marks the point at which the morphology of the site enters the model at all, and the beginning of the trajectory that will make it the dominant criterion from FBA12 onwards.

**Fig 20:**
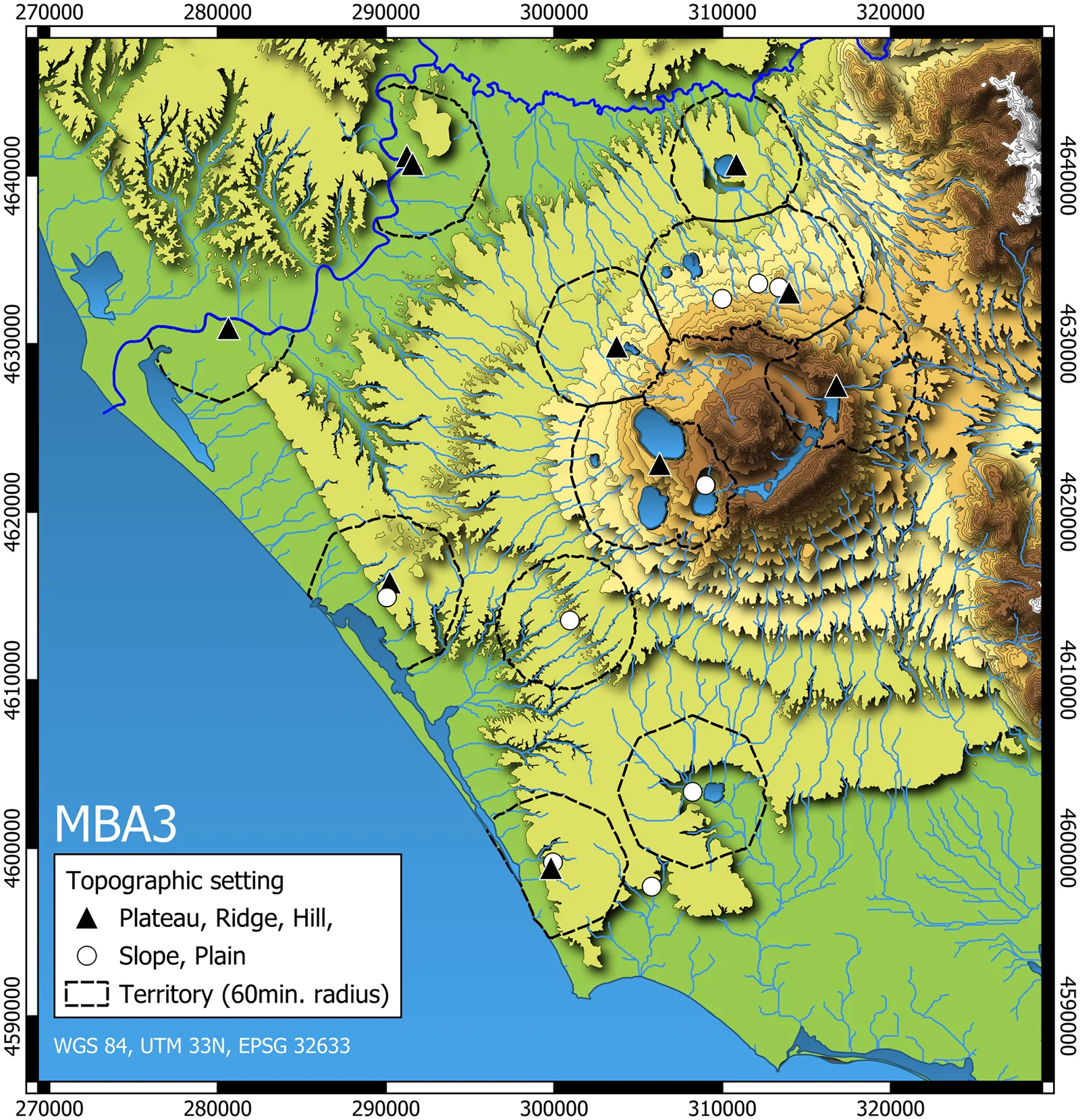
Phase map with Middle Bronze Age settlements, subphase 3. The sites are classified according to their topographic setting into two groups: those occupying more readily defensible positions, and those situated in more open settings. Site locations, reconstructed shoreline and lakes/lagoons as determined by Alessandri (2013). Background, elaborated by the author from TinItaly dataset (Tarquini S., I. Isola, M. Favalli, A. Battistini, G. Dotta, 2023. TINITALY, a digital elevation model of Italy with a 10 meters cell size (Version 1.1). Istituto Nazionale di Geofisica e Vulcanologia (INGV). https://doi.org/10.13127/tinitaly/1.1). The dataset is published under a CC BY 4.0 license (downloaded from https://tinitaly.pi.ingv.it/Download_Area1_1.html). Map created with QGIS 3.40, (www.qgis.org).

This phase is indeed characterized by a numerical rise in settlements located in defensible and moderately defensible positions. Equally noteworthy is the territorial distribution of defensible settlements (Fig 20). In the Colli Albani, they appear to be positioned at three vertices of an imaginary square, and whose territories, reconstructed through the Bubble Model [19], are practically all tangent to one another at a distance slightly less than a 60-minute walk. Along the coast, defensible settlements are distributed at intervals of approximately 4 hours’ walking distance (Ficana to Pratica di Mare, 235 min.; Pratica di Mare to Colle Rotondo, 259 min.). Furthermore, five of the settlements in defensible or moderate defensible positions persist into the subsequent phase: Ficana (L1D), Pratica di Mare (L4A), Colle dei Cappuccini (3), Rome (F19/F22), and Colle della Mola (4); the first four continue well into the Iron Age.

#### RBA (1300−1150 BCE): The emerging of social complexity.

**Fig 21:**
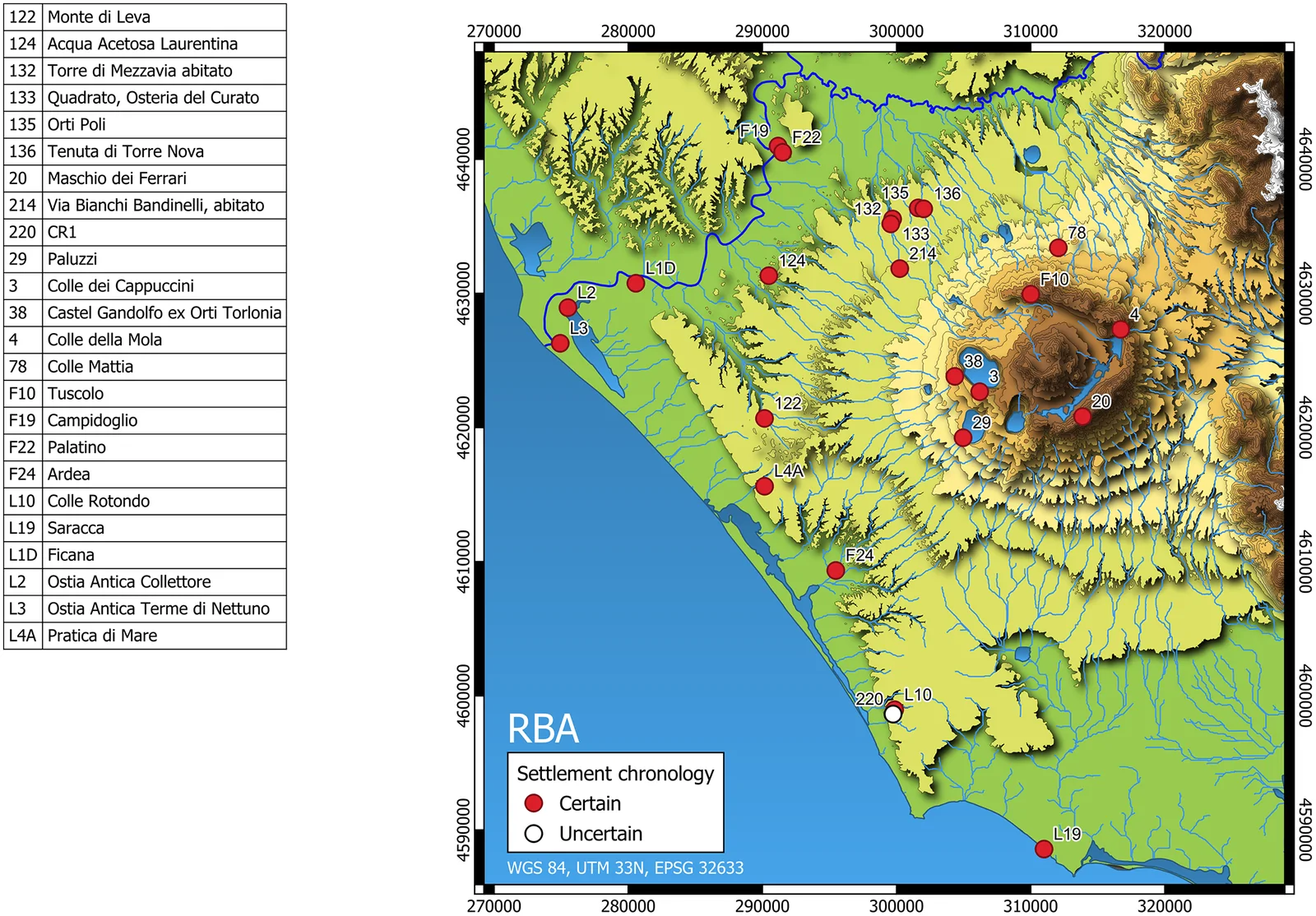
Phase map with Recent Bronze Age settlements. Site locations, reconstructed shoreline and lakes/lagoons as determined by Alessandri (2013). Background, elaborated by the author from TinItaly dataset (Tarquini S., I. Isola, M. Favalli, A. Battistini, G. Dotta, 2023. TINITALY, a digital elevation model of Italy with a 10 meters cell size (Version 1.1). Istituto Nazionale di Geofisica e Vulcanologia (INGV). https://doi.org/10.13127/tinitaly/1.1). The dataset is published under a CC BY 4.0 license (downloaded from https://tinitaly.pi.ingv.it/Download_Area1_1.html). Map created with QGIS 3.40, (www.qgis.org).

As already observed for MBA12, where exploratory dynamics forced the model to rely on broad decision criteria, the RBA phase (Fig 21) again exhibits a marked weakening of the landscape signal. Here too the classification does not exceed chance expectation on either metric, and the divergence between training and validated performance is the widest in the sequence. The environmental variables considered here therefore do not distinguish RBA settlements from pseudo-absence points, and neither the split frequencies nor the mean SHAP values for this phase can be read as evidence of environmental preference.

**Fig 22:**
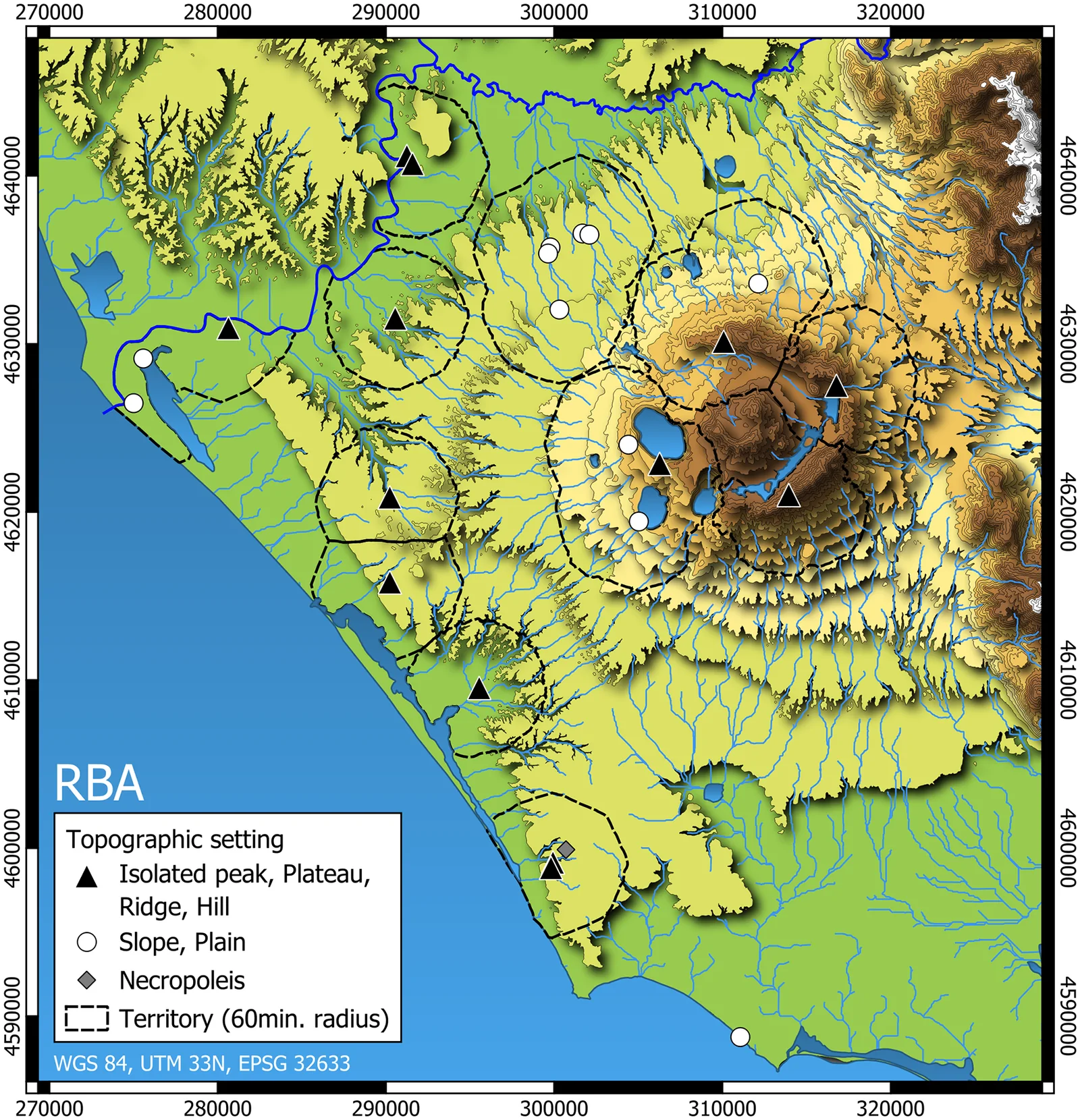
Phase map with Recent Bronze Age settlements. The sites are classified according to their topographic setting into two groups: those occupying more readily defensible positions, and those situated in more open settings. Site locations, reconstructed shoreline and lakes/lagoons as determined by Alessandri (2013). Background, elaborated by the author from TinItaly dataset (Tarquini S., I. Isola, M. Favalli, A. Battistini, G. Dotta, 2023. TINITALY, a digital elevation model of Italy with a 10 meters cell size (Version 1.1). Istituto Nazionale di Geofisica e Vulcanologia (INGV). https://doi.org/10.13127/tinitaly/1.1). The dataset is published under a CC BY 4.0 license (downloaded from https://tinitaly.pi.ingv.it/Download_Area1_1.html). Map created with QGIS 3.40, (www.qgis.org).

In the Colli Albani, the occupation of Maschio dei Ferrari completes the settlement framework anticipated in the previous phase, effectively saturating the Vulcano Laziale. Along the coast, newly founded defensible sites such as Ardea and Monte di Leva fill the gaps left open earlier, and their presence appears to structure surrounding settlement patterns: within their spheres of influence, smaller non-defensible sites sometimes tend to cluster, suggesting forms of hierarchical or gravitational organization (Fig 22). A notable exception is the cluster east of Rome, where no settlement emerges as a clear focal point. In the immediate vicinity of Colle Rotondo, the earliest cremation cemetery of *Latium vetus*, Cavallo Morto, marks a striking shift in funerary practice, replacing earlier cave inhumations that, as far as we know, were accessible to the entire community. The small size of these cemeteries, here and in the ensuing Final Bronze Age, implies a more restricted access to burial, pointing to emerging social differentiation. At the same time, the first appearances of Mycenaean and Italo-Mycenaean pottery at Rome [46] and at Casale Nuovo to the south signal the integration of the region into long-distance exchange networks that transmitted not only goods but also ideas and technical expertise. Overall, the Recent Bronze Age stands out as a rapidly evolving phase in which multiple innovations converge, reflecting profound shifts in socio-economic structures and marking the onset of trajectories that will become clearer in the following periods and will culminate on the eve of the Iron Age.

This may also account for the difficulty the model encounters in this phase. The normalized split frequencies show that topographic setting rises steadily across the sequence, from near-irrelevance in the earliest phases to complete dominance from FBA3 onwards, and the RBA falls almost exactly at the midpoint of that trajectory. It is plausible that settlements founded in this phase were selected according to criteria that were themselves in transition: some still following the hydrological logic of the preceding centuries, others already anticipating the topographic one that would prevail from the Final Bronze Age. A single model cannot separate two populations that respond to different rules, and the resulting signal is not weak so much as internally contradictory. On this reading, the failure of the RBA model differs in kind from that of MBA12: there the absence of a signal reflected expansion into a range of ecological niches, none of them dominant, whereas here it reflects the coexistence of an old criterion being abandoned and a new one not yet established. In both cases, periods of rapid change leave a landscape that is harder to predict, not because settlement choices were arbitrary, but because more than one logic was operating at once.

#### FBA12 (1150−1050 BCE): Territorial stabilisation.

**Fig 23:**
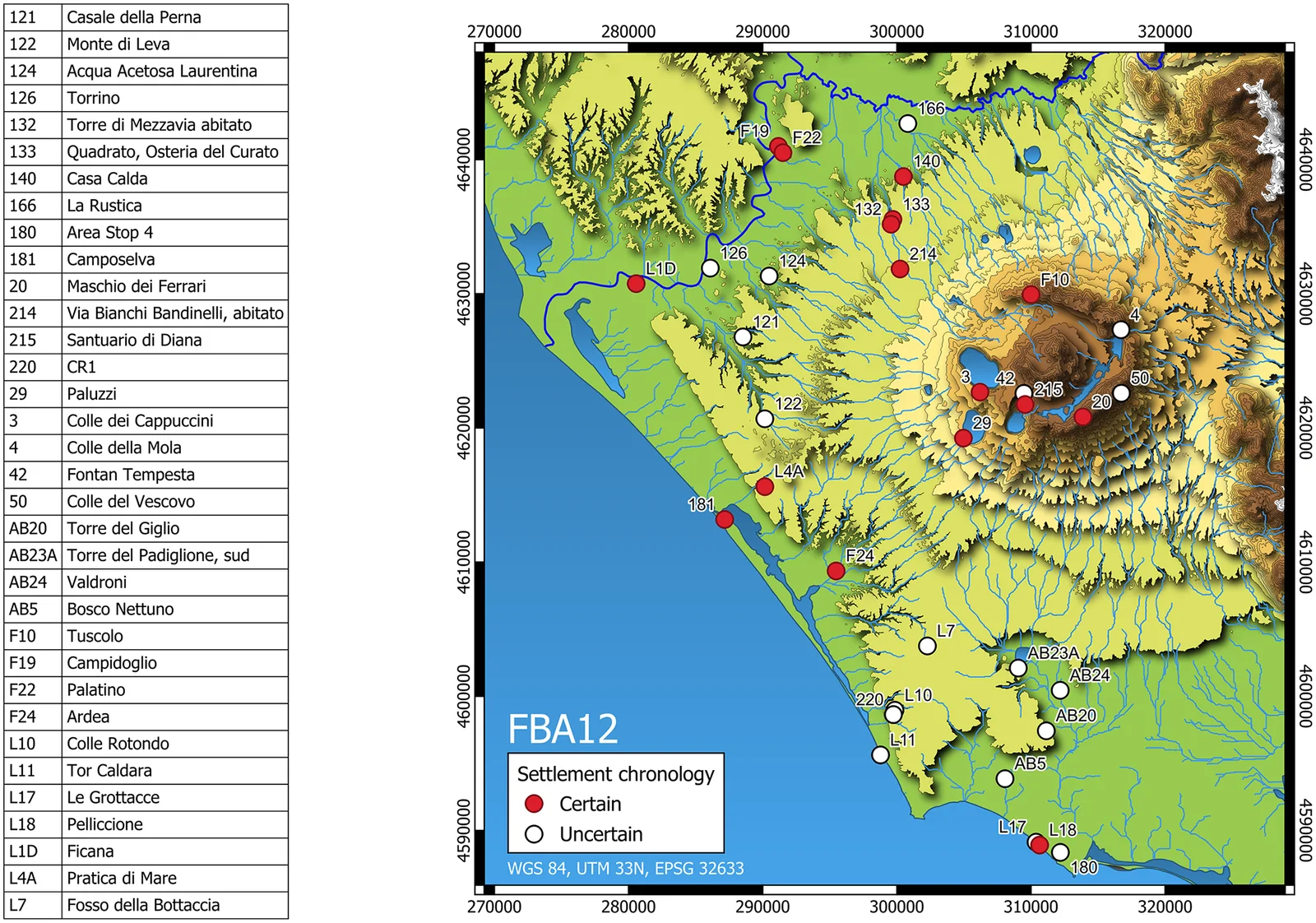
Phase map with Final Bronze Age settlements, subphases 1 and 2. For clarity, the site of Casale Nuovo, situated just beyond the boundaries of the study area, has been included. Site locations, reconstructed shoreline and lakes/lagoons as determined by Alessandri (2013). Background, elaborated by the author from TinItaly dataset (Tarquini S., I. Isola, M. Favalli, A. Battistini, G. Dotta, 2023. TINITALY, a digital elevation model of Italy with a 10 meters cell size (Version 1.1). Istituto Nazionale di Geofisica e Vulcanologia (INGV). https://doi.org/10.13127/tinitaly/1.1). The dataset is published under a CC BY 4.0 license (downloaded from https://tinitaly.pi.ingv.it/Download_Area1_1.html). Map created with QGIS 3.40, (www.qgis.org).

The FBA12 phase (Fig 23) displays a clear and internally coherent settlement signal. The classification obtained on the held-out partition was mathematically perfect on every metric, and while such a result cannot be taken at face value, since it rests on six test cases and may in principle arise from overfitting rather than from a genuine archaeological signal, the permutation test now confirms that the underlying separation is real: under repeated cross-validation the model performs well above chance on both criteria, and none of the 500 permutations reached the observed accuracy. The gap between training and validated performance is also among the narrowest in the sequence. The strength of the signal is therefore genuine, though more moderate than the perfect classification suggested.

The pattern is consistent with the broader trends identified in the diachronic analysis. The mean SHAP values indicate that, from this phase onward and through to the RMCAIII phase, settlement location is strongly associated with specific topographic configurations, suggesting the emergence of a markedly canalised settlement logic in which communities increasingly concentrated on morphologically distinctive and elevated locations.

The normalized split frequencies rank the predictors differently, placing C6 and altitude first and topographic setting only tenth. The two measures are not in conflict, since they quantify distinct properties: split frequencies record how often a variable is used to partition the data, across settlements and pseudo-absences alike, whereas the mean SHAP values reported here are computed on correctly predicted settlements only, and therefore measure how far each variable pushes the model towards site presence. A variable may be structurally useful in delimiting where sites are not found, and thus be selected frequently for splitting, without contributing much to their positive identification. Topographic setting behaves in the opposite way: used less often, but decisive when it is.

**Fig 24:**
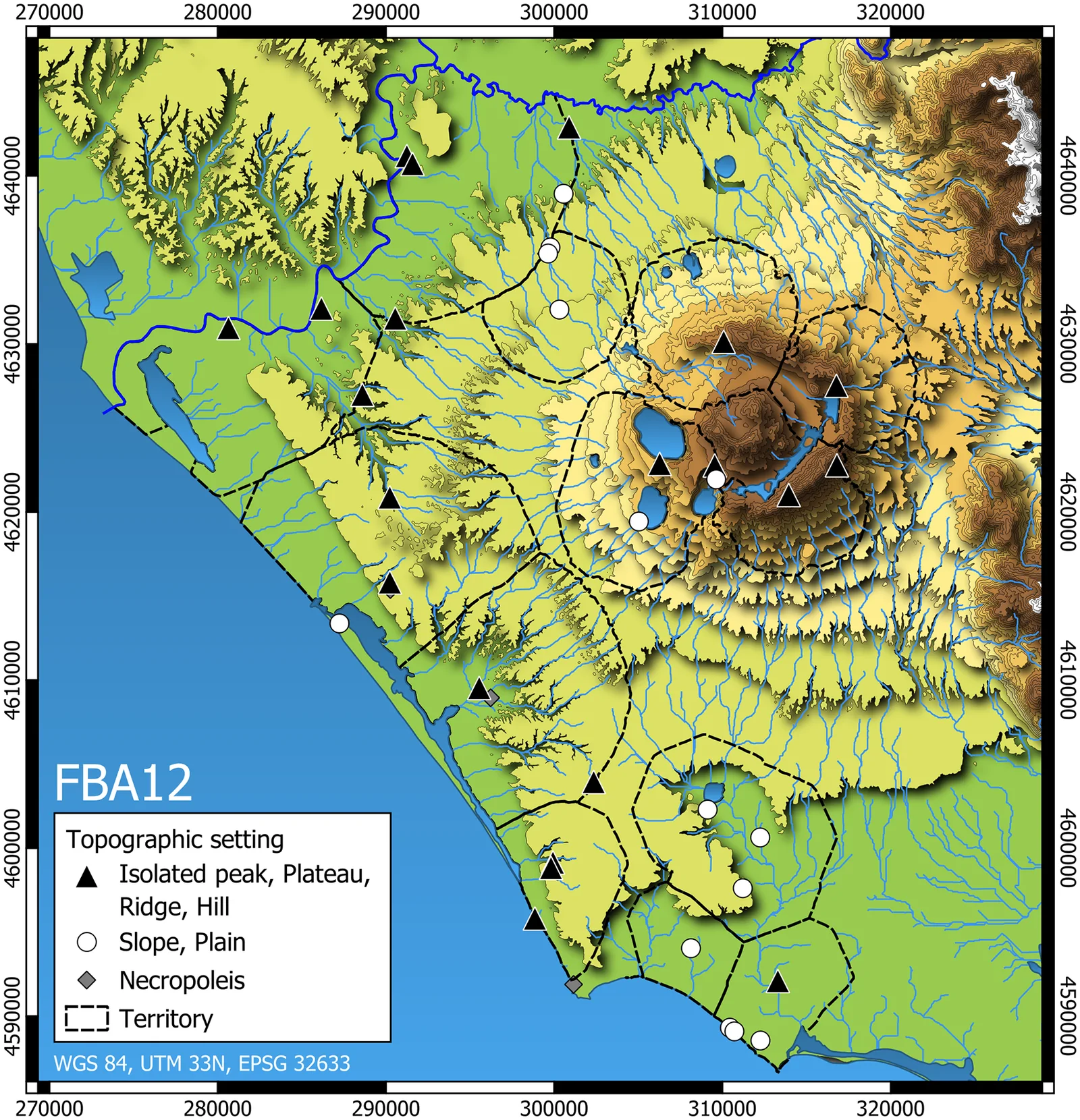
Phase map with Final Bronze Age settlements, subphases 1 and 2. The sites are classified according to their topographic setting into two groups: those occupying more readily defensible positions, and those situated in more open settings. Site locations, reconstructed shoreline and lakes/lagoons as determined by Alessandri (2013). Background, elaborated by the author from TinItaly dataset (Tarquini S., I. Isola, M. Favalli, A. Battistini, G. Dotta, 2023. TINITALY, a digital elevation model of Italy with a 10 meters cell size (Version 1.1). Istituto Nazionale di Geofisica e Vulcanologia (INGV). https://doi.org/10.13127/tinitaly/1.1). The dataset is published under a CC BY 4.0 license (downloaded from https://tinitaly.pi.ingv.it/Download_Area1_1.html). Map created with QGIS 3.40, (www.qgis.org).

At the territorial scale, the spatial configuration of sites, particularly the group of smaller settlements forming a ring around the Roman core at around 130 minutes from the Capitoline/Palatine system, suggests that the effective radius of control exercised by major, defensible centres may have nearly doubled (Fig 24). When the same radius is applied to the larger coastal settlements, their spheres of influence tend to be mutually tangent, with the notable exception of the boundary between Ardea and Pratica di Mare, whereas in the remaining areas a limit of approximately one hour’s walking distance appears to persist. Several additional necropoleis have been identified in the region, all consisting of few and isolated cremation burials, broadly comparable in conception to those of the RBA. Overall, the FBA12 phase appears to stand in direct continuity with the preceding period, without marked discontinuities, while consolidating the settlement logic that emerges at the end of the Bronze Age.

#### FBA3 (1050−950 BCE): Institutionalisation of inequality.

**Fig 25:**
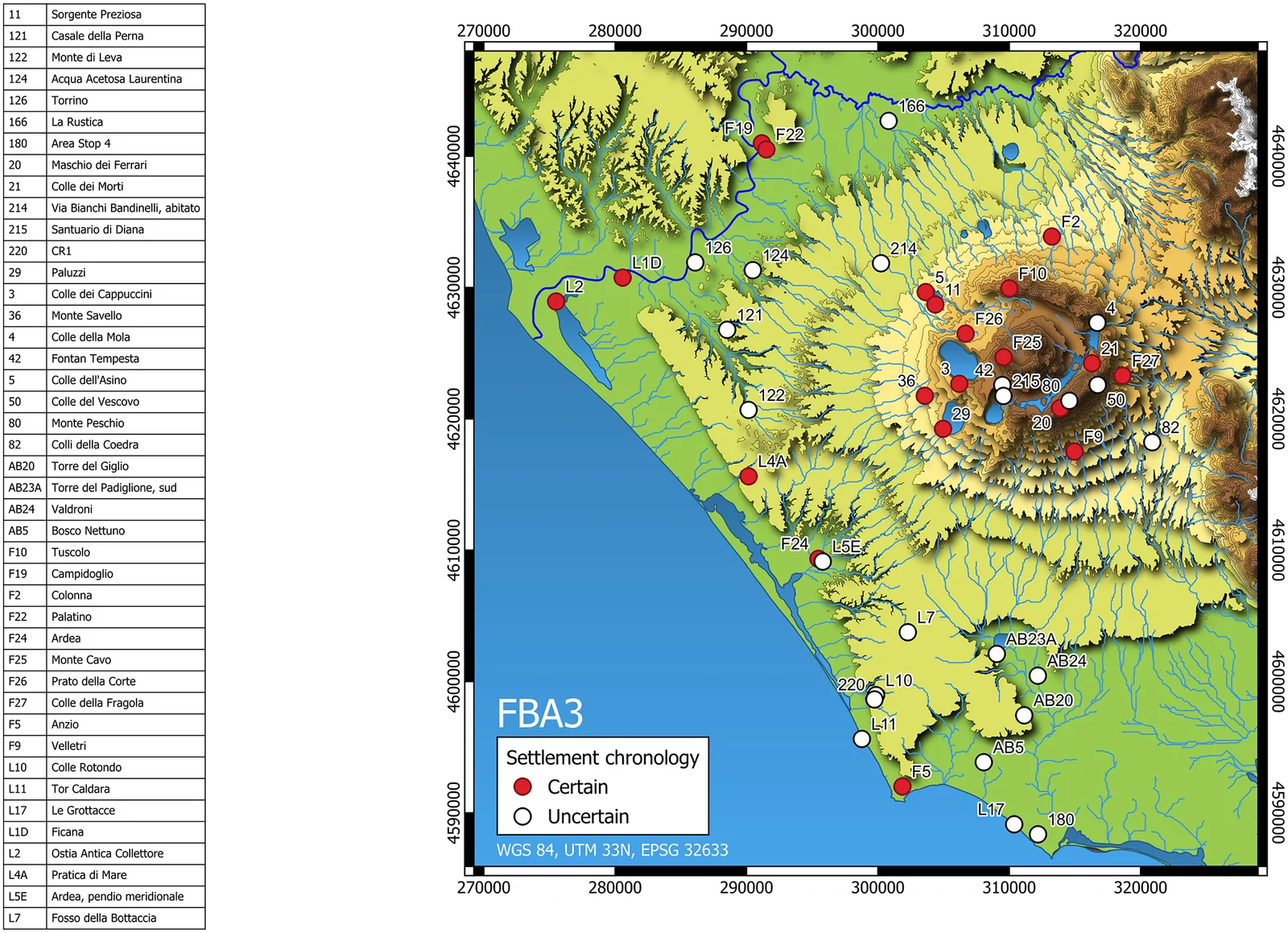
Phase map with Final Bronze Age settlements, subphase 3 (also known as Roma Colli Albani, subphase I). Site locations, reconstructed shoreline and lakes/lagoons as determined by Alessandri (2013). Background, elaborated by the author from TinItaly dataset (Tarquini S., I. Isola, M. Favalli, A. Battistini, G. Dotta, 2023. TINITALY, a digital elevation model of Italy with a 10 meters cell size (Version 1.1). Istituto Nazionale di Geofisica e Vulcanologia (INGV). https://doi.org/10.13127/tinitaly/1.1). The dataset is published under a CC BY 4.0 license (downloaded from https://tinitaly.pi.ingv.it/Download_Area1_1.html). Map created with QGIS 3.40, (www.qgis.org).

The FBA3 phase maintains a solid level of model performance, confirming the consolidation of a structured settlement signal (Fig 25). The classification exceeds chance expectation on accuracy, though the result on recall falls just short of the conventional threshold, and the gap between training and validated performance is the second narrowest in the sequence**,** an indication that what the model learns in this phase generalises well to cases it has not seen. The environmental and topographic signatures governing site location in FBA3 therefore remain highly discriminative, and settlement choices appear strongly constrained by specific landscape configurations, allowing the model to operate with narrow decision boundaries within an otherwise stable and well-canalised settlement system. This time, both the mean SHAP values and the normalized split frequencies consistently identify the topographic setting as by far the most important driver in the selection (or long-term maintenance) of settlement location.

The FBA3 phase marks the first clear appearance of the so-called Rome–Colli Albani ceramic facies (subphase I). This facies is defined primarily through funerary material culture: burial practices continue to be characterised by cremation, reserved for restricted segments of the population and accompanied by highly standardised assemblages. Within this framework, however, several important innovations emerge. Most striking is the sharp increase in the number of necropoleis and isolated graves, which rises from only three attestations in FBA12 to thirty-six in FBA3, indicating the rapid regional diffusion of the rite and, by extension, of the value system underpinning it. This expansion is further reflected in the spatial distribution of funerary evidence, which now covers almost the entirety of the *Latium vetus*, with the southernmost occurrence extending even beyond the study area, at Bosco del Polverino [47].

Two features of the grave goods are particularly revealing. The first is the widespread miniaturisation of selected ceramic and metal objects, including razors, lances, swords, greaves and double shields [3,48,49]. Although the precise meaning of this practice remains elusive, it closely parallels the prominent role of miniature vessels in ritual contexts, especially in chthonic cults and in votive deposits associated with water bodies [2]. In the study area, a well-known example is the Laghetto del Monsignore at Campoverde, where thousands of miniature vessels, together with high-value artefacts in full size, were deposited over a long chronological span, from RMCAIIA, with possible earlier elements in FBA3, through to the fifth century BCE [50]. The second salient feature is the exceptional wealth of many funerary assemblages, exemplified by the necropolis of Santa Palomba, where miniature bronze chariots frequently accompany the cremation urns [51]. Notably, such rich assemblages are often associated with juvenile individuals, as in tomb 5 at Le Caprine, where a child of approximately two years of age was buried with an exceptionally elaborate set of grave goods [52].

Taken together, the selective adoption of the cremation rite, the richness and standardisation of the assemblages, their association with individuals of all ages, and the pervasive role of miniaturisation in both funerary and votive contexts point to the emergence of a dominant elite whose status was inherited at birth, marking the institutionalization of inequality. Funerary ritual appears to have functioned as a key medium for the public expression and reproduction of this status, and possibly for the post-mortem elevation of the deceased to a semi-divine or ancestral sphere.

**Fig 26:**
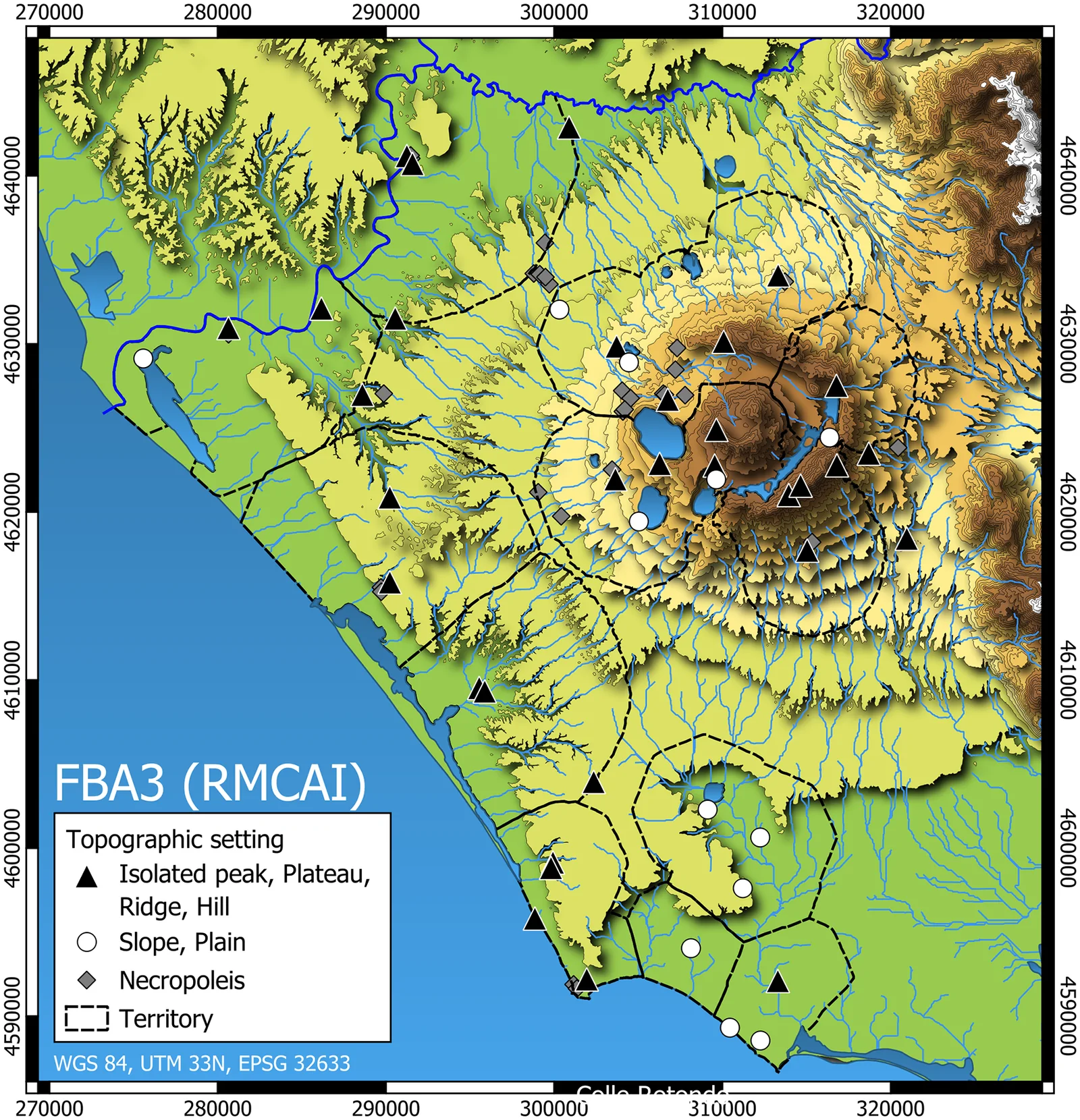
Phase map with Final Bronze Age settlements, subphase 3. The sites are classified according to their topographic setting into two groups: those occupying more readily defensible positions, and those situated in more open settings. Site locations, reconstructed shoreline and lakes/lagoons as determined by Alessandri (2013). Background, elaborated by the author from TinItaly dataset (Tarquini S., I. Isola, M. Favalli, A. Battistini, G. Dotta, 2023. TINITALY, a digital elevation model of Italy with a 10 meters cell size (Version 1.1). Istituto Nazionale di Geofisica e Vulcanologia (INGV). https://doi.org/10.13127/tinitaly/1.1). The dataset is published under a CC BY 4.0 license (downloaded from https://tinitaly.pi.ingv.it/Download_Area1_1.html). Map created with QGIS 3.40, (www.qgis.org).

The existence of elites plausibly rooted in kinship-based groups accords well with the simultaneous emergence of incipient hierarchical differentiation among defensible settlements within the same territories, a locational parameter that from this point onward strongly shapes settlement choices (Fig 26). Such sites likely functioned as the seats of these elites, alongside other settlements that may have fulfilled roles extending beyond purely residential functions. Further, as previously suggested by R. Peroni [2], in socio-economic systems where land ownership remained largely communal, elite economic strategies were necessarily directed toward alternative domains; among these, salt production appears particularly significant. In the *Latium vetus*, the earliest evidence for this activity dates to the FBA12 phase, at the site of Pelliccione [53], where salt was most likely produced using briquetage techniques.

#### RMCAIIA (950−880 BCE): Elite expansion.

**Fig 27:**
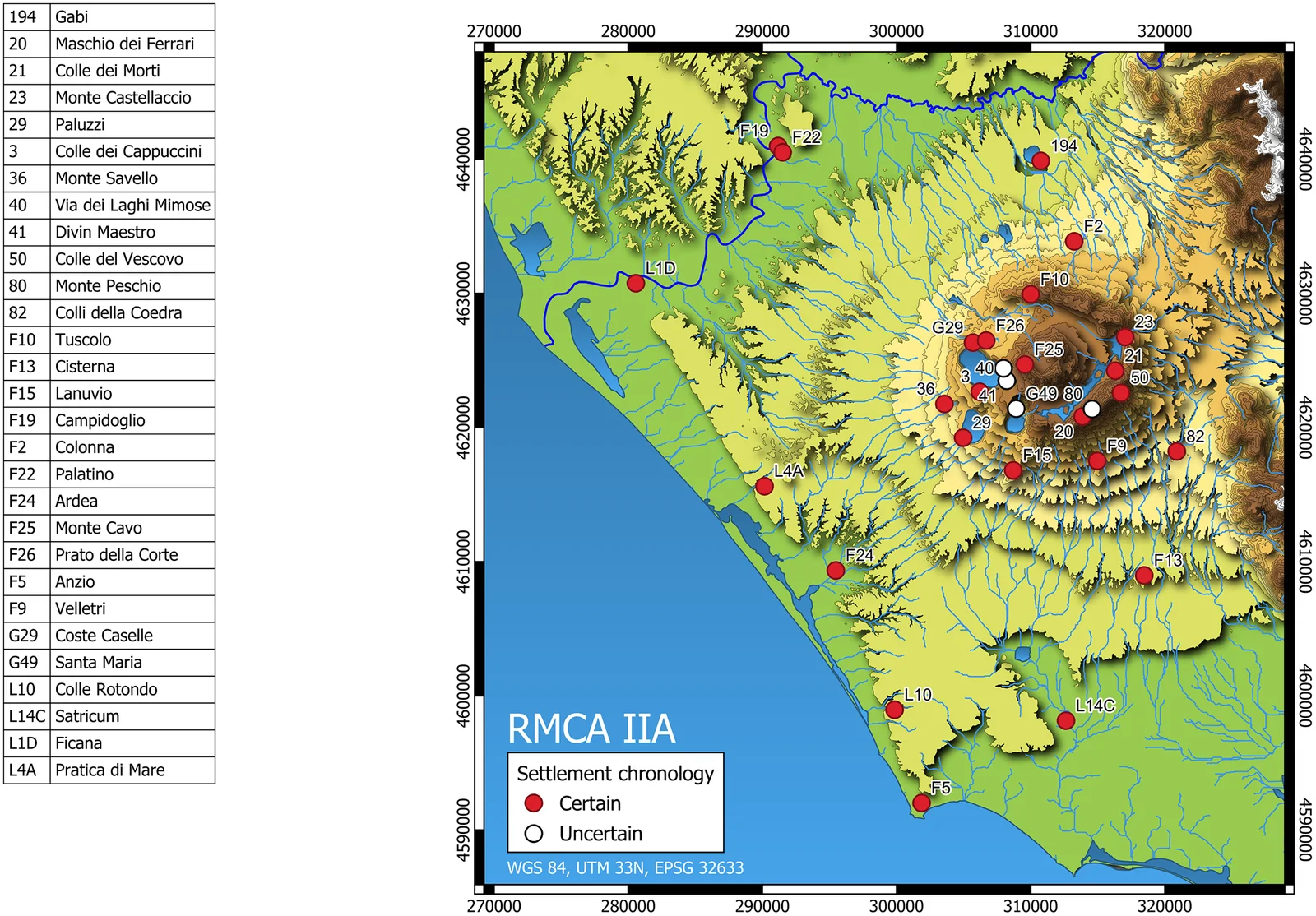
Phase map with Roma Colli Albani facies settlements, subphase IIA. Site locations, reconstructed shoreline and lakes/lagoons as determined by Alessandri (2013). Background, elaborated by the author from TinItaly dataset (Tarquini S., I. Isola, M. Favalli, A. Battistini, G. Dotta, 2023. TINITALY, a digital elevation model of Italy with a 10 meters cell size (Version 1.1). Istituto Nazionale di Geofisica e Vulcanologia (INGV). https://doi.org/10.13127/tinitaly/1.1). The dataset is published under a CC BY 4.0 license (downloaded from https://tinitaly.pi.ingv.it/Download_Area1_1.html). Map created with QGIS 3.40, (www.qgis.org).

The RMCAIIA phase (Fig 27) yields the strongest model of the entire sequence, and the one that generalises best: the classification exceeds chance expectation at the resolution limit of the permutation test on both metrics, and the gap between training and validated performance is the narrowest recorded. What the model learns in this phase, in other words, generalises almost without loss to settlements it has not seen, which is the signature of a settlement logic that is not merely selective but consistent.

This pattern is closely mirrored by the feature-importance metrics. Both the mean SHAP values and the normalized split frequencies converge in identifying topographic setting as the dominant driver of settlement location. Its mean SHAP value is by far the highest recorded for any predictor in any phase, indicating a strong and consistently positive contribution to site prediction, while its normalized split frequency reaches the maximum possible value, demonstrating that the variable is systematically selected during tree construction to reduce impurity. As in FBA3, and unlike FBA12, here structural importance and predictive contribution are aligned at the top of the ranking, pointing to a mature and internally coherent settlement logic in which topographic configuration functions as both a primary filter and a decisive attractor.

Secondary variables play a clearly subordinate but not negligible role. Proximity to rivers returns the second highest mean SHAP value of the phase, and its split frequency is likewise the second highest, so that watercourses retain a consistent positive contribution alongside the dominant topographic criterion. Altitude follows closely, consistent with its coupling to topographic preferences rather than acting as an independent driver, while the remaining hydrological variables contribute positively but weakly. What distinguishes this phase is therefore not the disappearance of the earlier environmental criteria but their subordination: topographic setting contributes roughly four times as much as any other predictor, and the variables that had dominated the earlier phases persist as secondary considerations rather than being displaced altogether.

**Fig 28:**
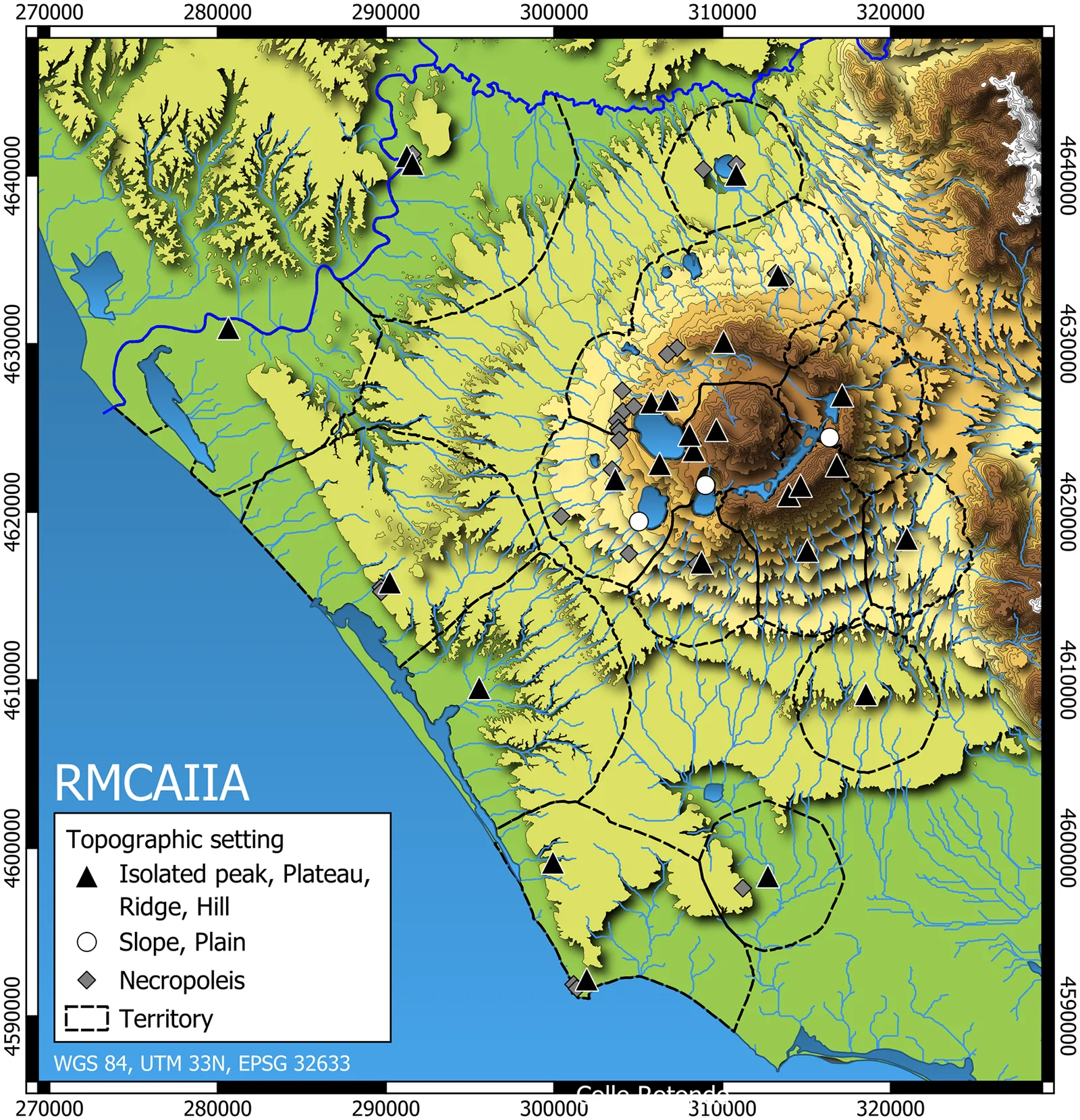
Phase map with Roma Colli Albani facies settlements, subphase IIA. The sites are classified according to their topographic setting into two groups: those occupying more readily defensible positions, and those situated in more open settings. Site locations, reconstructed shoreline and lakes/lagoons as determined by Alessandri (2013). Background, elaborated by the author from TinItaly dataset (Tarquini S., I. Isola, M. Favalli, A. Battistini, G. Dotta, 2023. TINITALY, a digital elevation model of Italy with a 10 meters cell size (Version 1.1). Istituto Nazionale di Geofisica e Vulcanologia (INGV). https://doi.org/10.13127/tinitaly/1.1). The dataset is published under a CC BY 4.0 license (downloaded from https://tinitaly.pi.ingv.it/Download_Area1_1.html). Map created with QGIS 3.40, (www.qgis.org).

Taken together, the convergence of performance and importance metrics suggests that RMCAIIA represents a phase of consolidated selectivity. Settlement choices are anchored to a narrowly defined set of landscape configurations, dominated by topographic setting, allowing the model to operate with high confidence and minimal error (Fig 28). This is the phase in which the environmental logic of settlement is most fully realised: the smallest loss between training and validated performance, indicate that the criteria governing settlement location were applied with unusual consistency. Rather than a breakdown or a loosening of the spatial logic established in the Late Bronze Age, RMCAIIA appears to represent its point of maximum coherence, at the threshold of the Early Iron Age.

More specifically, the coastal sectors, the areas close to the Tiber, and the northern portion of the Colli Albani remain largely stable. In the southern Colli Albani, a process of territorial reorganisation, still operating within the settlement logics identified thus far, leads to the emergence of Lanuvio, Cisterna and Satricum, proto-urban centres that would persist well beyond the Iron Age.

From a socio-economic perspective, this pattern is best understood as the spatial consolidation of processes already observed in the preceding phase. The incipient hierarchisation among defensible settlements identified for the FBA3 becomes fully operational in the RMCAIIA, as elevated and morphologically distinctive locations function as stable territorial anchors through which elite power was exercised, rendered visible, and reproduced. While the core locational logic remains highly selective and tightly anchored to topographic setting, other lines of evidence point to the emergence of a more articulated settlement system, in which functional differentiation accompanies an increasingly hierarchical territorial organisation. Settlement placement thus becomes a normative act embedded within a structured political economy, in which social regulation increasingly outweighs environmental opportunity alone, a development that the environmental model, by its nature, cannot capture, and which must be inferred from the archaeological record itself.

A further indication of the expansive dynamics characterising this phase is offered by the necropoleis. During the RMCAIIA phase, Rome witnesses a systematic relocation of funerary areas away from the urban core, likely driven by the spatial demands of an expanding settlement. Burial grounds are first displaced from the area of the Arch of Augustus to that of the Temple of Antoninus and Faustina in RMCAIIA, and subsequently, during RMCAIIA2 or at the latest at the onset of RMCAIIB, moved further to the Esquiline Hill. A comparable reconfiguration can be inferred for the Capitoline necropoleis, which shift from the Forum of Caesar to the Forum of Augustus, and later, in RMCAIIB, to the Quirinal Hill [3,9,54].

Similar dynamics are evident beyond Rome. At Pratica di Mare, the necropolis located on the main plateau is confined to the Final Bronze Age, while in RMCAIIA burials are relocated just outside it, suggesting that the plateau itself had by then become an inhabited area [55]. An analogous process has been proposed for Ardea, where the plateau of Civitavecchia appears to have been occupied at the beginning of the Iron Age, and possibly already from the Final Bronze Age [56].

The funerary record also provides further insights into the organisation and internal structuring of society which once again displays substantial innovations. Most notably, this phase marks a decisive shift from cremation to inhumation as the dominant burial rite. While cremation does not disappear entirely, it becomes increasingly restricted and appears to be reserved for the most eminent individuals, suggesting a growing differentiation in access to ritual practices. Inhumation, by contrast, becomes the normative mode of burial, signalling a redefinition of funerary behaviour at the level of the broader community. This transformation is not merely ritual but social in nature, as it reflects changing mechanisms of identity construction and social reproduction.

At the same time, the internal organisation of necropoleis provides further clues to the structuring of social groups. The spatial arrangement of burials at Osteria dell’Osa [57], in particular, is characterised by the clustering of graves into distinct groups, a pattern that plausibly reflects segmentation along kinship lines. Rather than representing a homogeneous burial ground, the necropolis appears organised into discrete family units, each maintaining its own funerary space across generations. Within this framework, the selective persistence of cremation for high-status individuals can be interpreted as a strategy of distinction adopted by dominant lineages, reinforcing their social pre-eminence through the maintenance of an exceptional ritual practice. Together, the coexistence of different rites and the spatial segmentation of burial grounds point to a society in which inequality was no longer merely expressed through wealth or display but structurally embedded in both ritual norms and genealogical organisation.

#### RMCAIIB (880−800 BCE): Persistence of topographic control.

**Fig 29:**
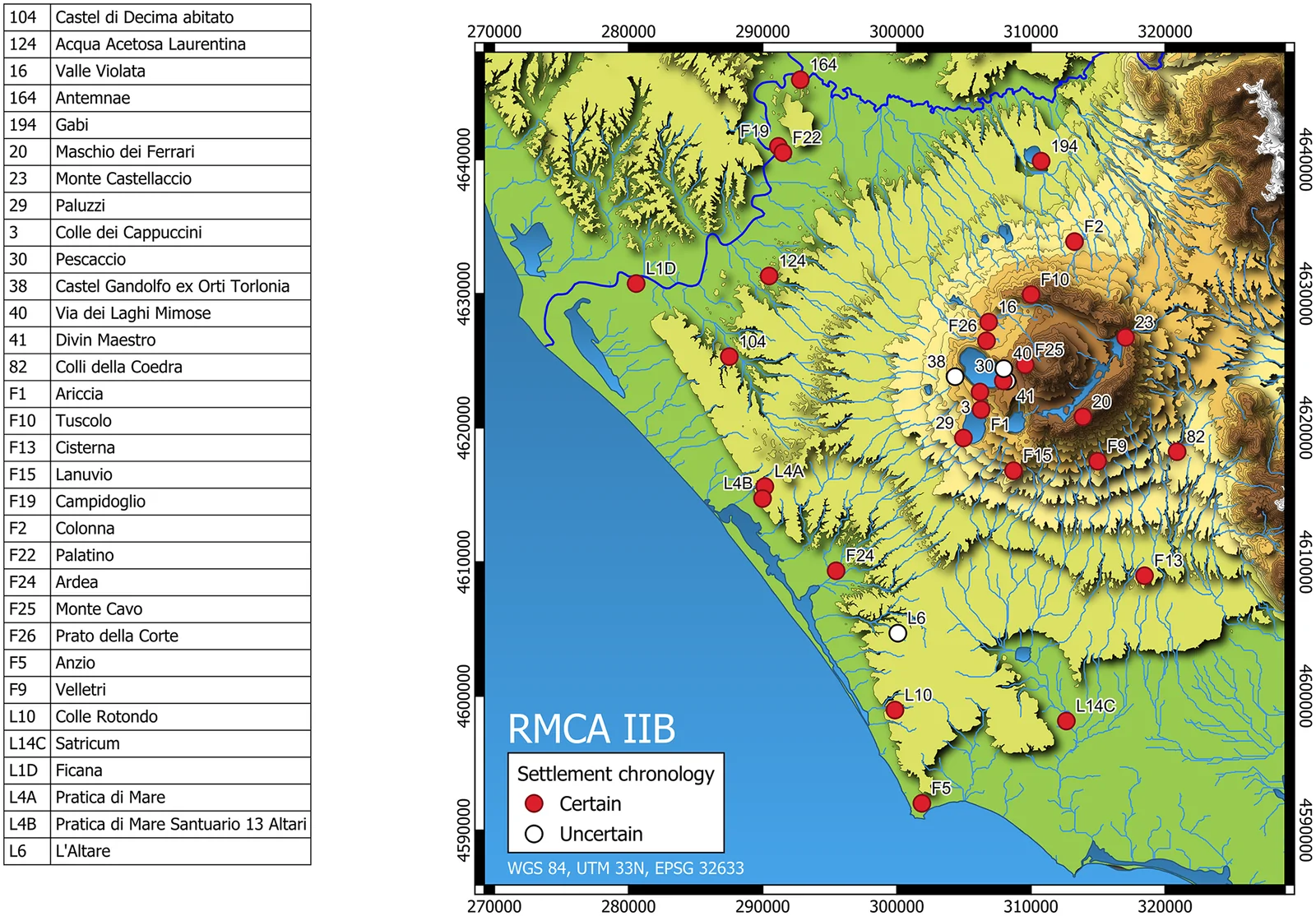
Phase map with Roma Colli Albani facies settlements, subphase IIB. Site locations, reconstructed shoreline and lakes/lagoons as determined by Alessandri (2013). Background, elaborated by the author from TinItaly dataset (Tarquini S., I. Isola, M. Favalli, A. Battistini, G. Dotta, 2023. TINITALY, a digital elevation model of Italy with a 10 meters cell size (Version 1.1). Istituto Nazionale di Geofisica e Vulcanologia (INGV). https://doi.org/10.13127/tinitaly/1.1). The dataset is published under a CC BY 4.0 license (downloaded from https://tinitaly.pi.ingv.it/Download_Area1_1.html). Map created with QGIS 3.40, (www.qgis.org).

The RMCAIIB phase (Fig 29) confirms the persistence of a highly structured and selective settlement system. The classification exceeds chance expectation at the resolution limit of the permutation test on both metrics, as in RMCAIIA, and the gap between training and validated performance remains narrow. The environmental signature of settlement is therefore as clearly defined here as in the preceding phase, if marginally less sharply resolved.

This shift is nevertheless embedded within a stable hierarchy of predictors. Both mean SHAP values and normalized split frequencies continue to identify topographic setting as the dominant driver of settlement location. Its mean SHAP contribution is by far the highest among all predictors, five times that of the next variable, and its split frequency reaches the maximum value, indicating that it is systematically used to structure the forest and exerts a strong positive influence on site prediction.

Secondary predictors are markedly subordinate but uniformly positive. Altitude returns the second highest mean SHAP contribution, consistent with its close coupling to topographic preferences rather than an independent driving role, followed by springs and soil class C3. The remaining hydrological variables (rivers, sea and water bodies) contribute positively but weakly, despite moderate split frequencies, the sea in particular being used relatively often to partition the feature space while adding comparatively little to the prediction of sites. The environmental criteria that had governed settlement in the earlier phases therefore persist, but at roughly a fifth of the weight carried by topographic setting: they are subordinated rather than displaced.

The convergence of performance and importance metrics indicates that RMCAIIB represents a phase of continuity and stabilisation within the structured settlement logic established in the Late Bronze Age and consolidated in RMCAIIA. The locational rules anchored in topographic setting remain firmly in place, and the model identifies them with the same reliability as in the preceding phase, pointing to a settlement system that reproduces an established order rather than negotiating a new one. At this stage, the territory itself was likely approaching, if not already reaching, full saturation. Most ecologically and strategically viable locations had been occupied, leaving little room for further expansion without disrupting the existing balance. This condition of spatial closure also provides a powerful explanation for the crystallisation of settlement patterns detected by the model.

**Fig 30:**
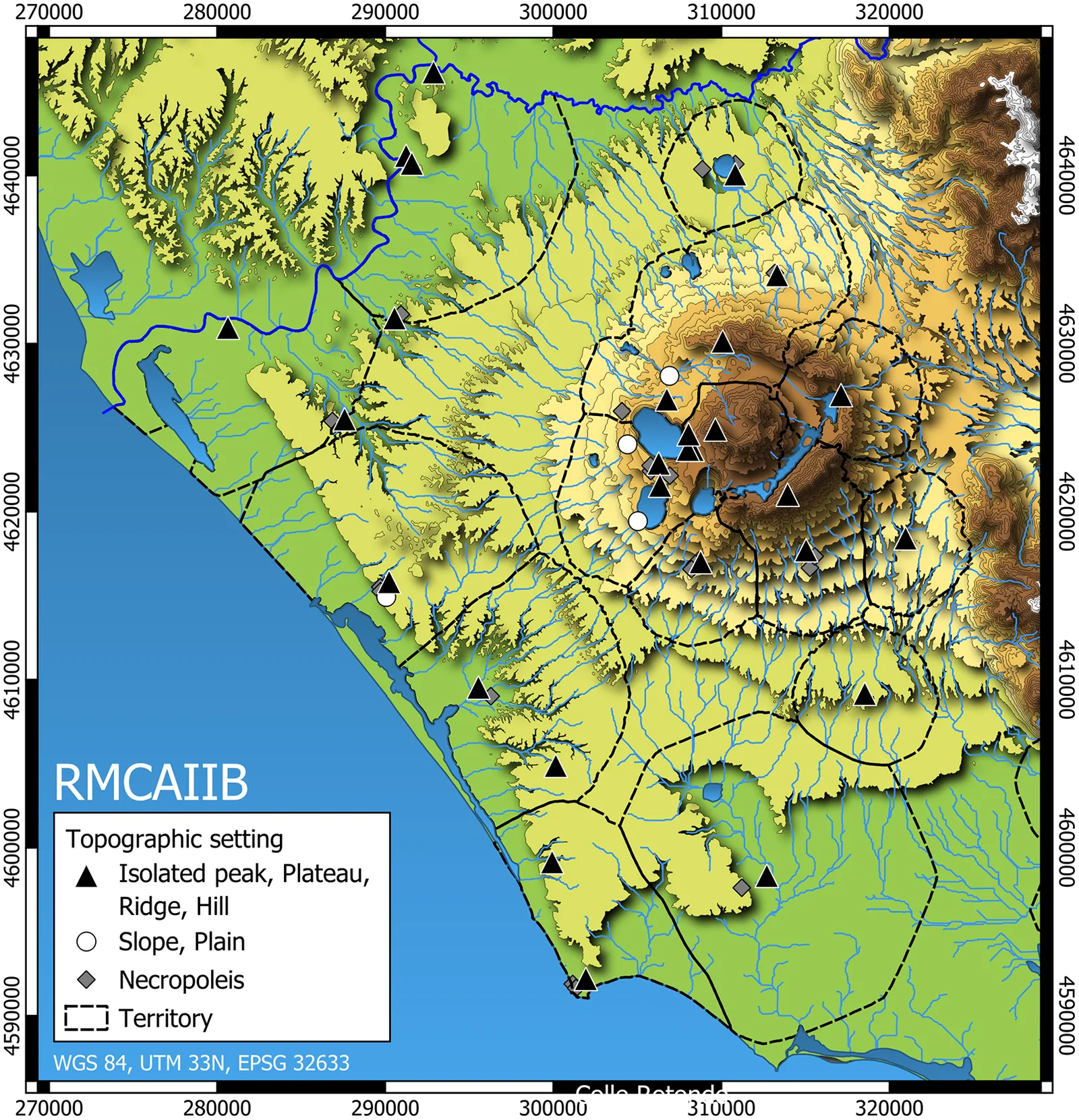
Phase map with Roma Colli Albani facies settlements, subphase IIB. The sites are classified according to their topographic setting into two groups: those occupying more readily defensible positions, and those situated in more open settings. Site locations, reconstructed shoreline and lakes/lagoons as determined by Alessandri (2013). Background, elaborated by the author from TinItaly dataset (Tarquini S., I. Isola, M. Favalli, A. Battistini, G. Dotta, 2023. TINITALY, a digital elevation model of Italy with a 10 meters cell size (Version 1.1). Istituto Nazionale di Geofisica e Vulcanologia (INGV). https://doi.org/10.13127/tinitaly/1.1). The dataset is published under a CC BY 4.0 license (downloaded from https://tinitaly.pi.ingv.it/Download_Area1_1.html). Map created with QGIS 3.40, (www.qgis.org).

At the territorial scale (Fig 30), the continuity of major centres and the limited diversification of secondary sites point to a political economy oriented toward the management of already integrated territories rather than their expansion. Most of the areas occupied by the major centres show evidence of spatial expansion. This is the case, as already noted, for Rome, which probably began to expand as early as RMCAIIA, but also for Pratica di Mare and Ardea, whose settlements extend onto the adjacent plateaus. In this phase, it is also possible to discern with some clarity an important territorial differentiation. If one considers the size of the settlements (where this can be estimated), two distinct organisational patterns seem to emerge. In the first, larger and dominant centres share the territory with much smaller, and plausibly subordinate, sites. This appears to be the case, for example, for Rome with Acqua Acetosa (and perhaps Antemnae), Ardea with l’Altare, and possibly Pratica di Mare with Tredici Altari. In the second pattern, by contrast, centres of comparable size coexist, as in the systems formed by Ficana and Castel di Decima; Tusculum with Colonna and Prato della Corte; Colle dei Cappuccini with Ariccia and Monte Cavo; and Anzio with Colle Rotondo. This evidence suggests the coexistence of a territorially organised system based on hierarchy, on the one hand, and a more federative arrangement, on the other, the latter being characteristic of the Alban Hills area. Although such federative systems are notoriously difficult to identify in the protohistoric archaeological record, they find echoes in later sources, such as the league of the *Populi Albenses*, which comprised thirty communities gravitating around the Colli Albani [8,58], or the configurations proposed for the Fiora Valley and the Marta basin [59].

Overall, RMCAIIB emerges as a phase of socio-political maturity: a landscape no longer shaped by transformation, but by the reproduction of a hierarchical or federative order that had already proven effective. In this sense, the phase represents a plateau, in which territorial saturation and institutional stability together underpin the long-term persistence of the political geography into the subsequent periods.

#### RMCAIII (800−725 BCE): Stability of principles, complexity of outcomes.

**Fig 31:**
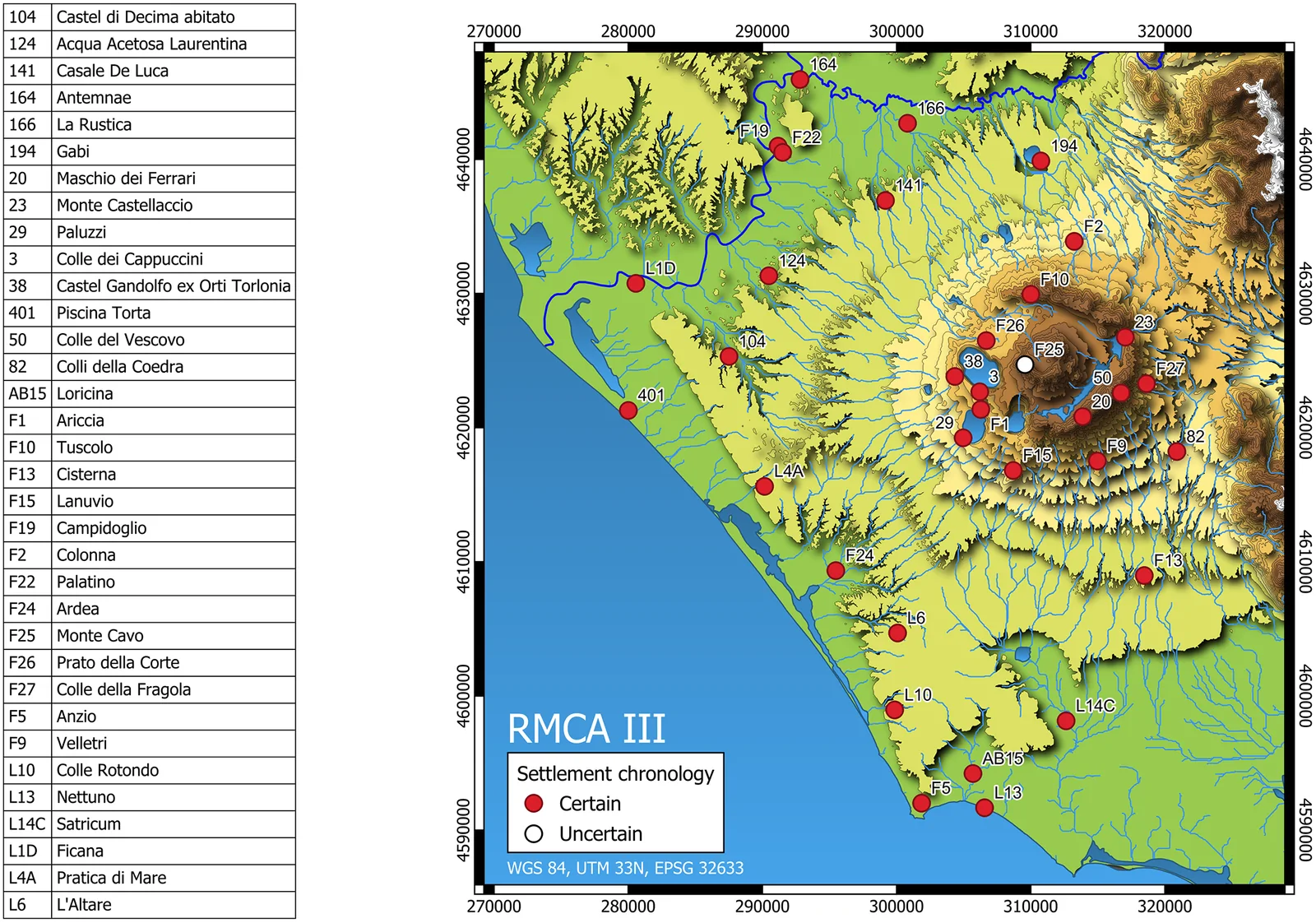
Phase map with Roma Colli Albani facies settlements, subphase III. Site locations, reconstructed shoreline and lakes/lagoons as determined by Alessandri (2013). Background, elaborated by the author from TinItaly dataset (Tarquini S., I. Isola, M. Favalli, A. Battistini, G. Dotta, 2023. TINITALY, a digital elevation model of Italy with a 10 meters cell size (Version 1.1). Istituto Nazionale di Geofisica e Vulcanologia (INGV). https://doi.org/10.13127/tinitaly/1.1). The dataset is published under a CC BY 4.0 license (downloaded from https://tinitaly.pi.ingv.it/Download_Area1_1.html). Map created with QGIS 3.40, (www.qgis.org).

**Fig 32:**
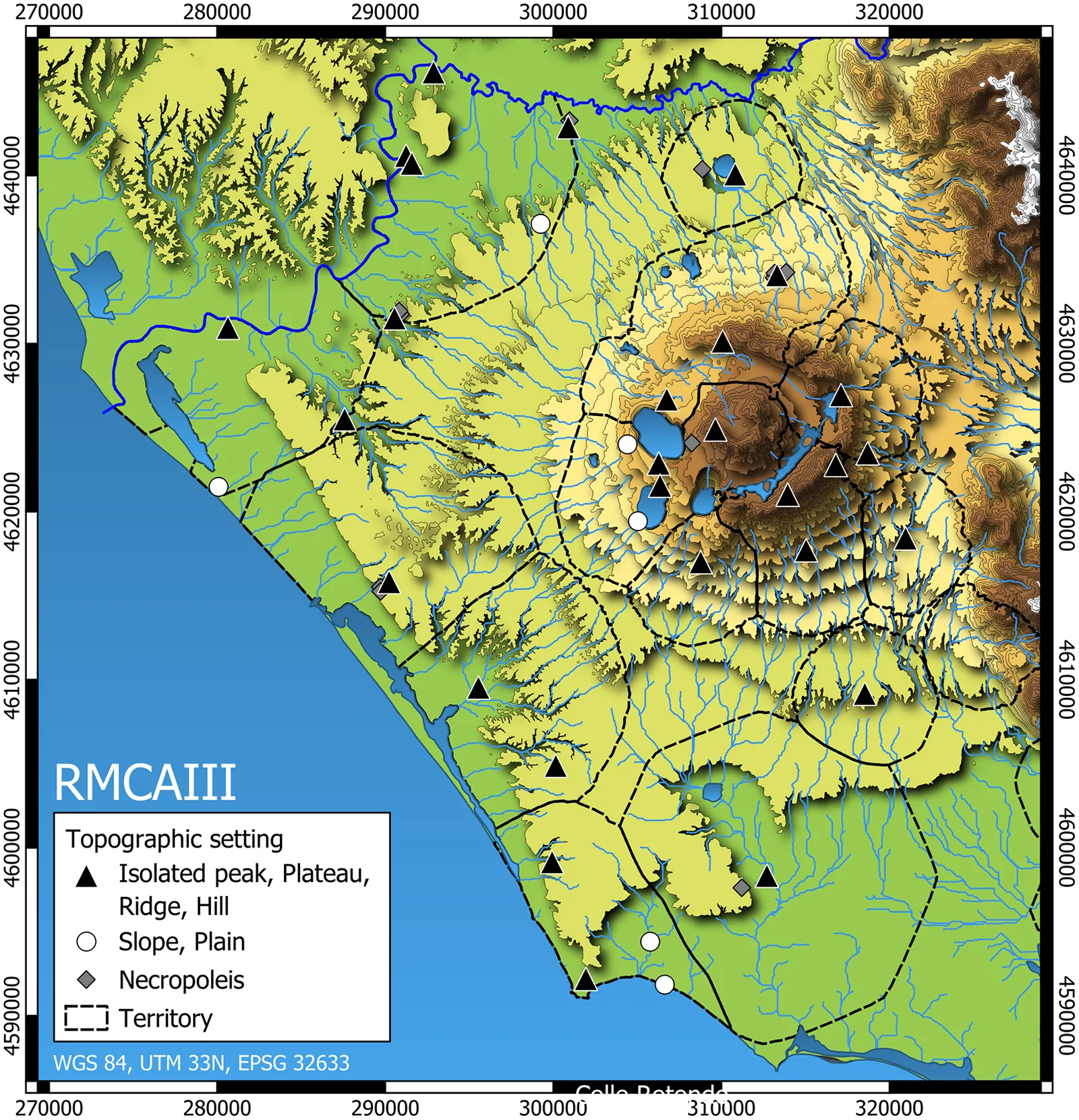
Phase map with Roma Colli Albani facies settlements, subphase III. The sites are classified according to their topographic setting into two groups: those occupying more readily defensible positions, and those situated in more open settings. Site locations, reconstructed shoreline and lakes/lagoons as determined by Alessandri (2013). Background, elaborated by the author from TinItaly dataset (Tarquini S., I. Isola, M. Favalli, A. Battistini, G. Dotta, 2023. TINITALY, a digital elevation model of Italy with a 10 meters cell size (Version 1.1). Istituto Nazionale di Geofisica e Vulcanologia (INGV). https://doi.org/10.13127/tinitaly/1.1). The dataset is published under a CC BY 4.0 license (downloaded from https://tinitaly.pi.ingv.it/Download_Area1_1.html). Map created with QGIS 3.40, (www.qgis.org).

The RMCAIII phase (Fig 31, 32) marks a perceptible attenuation of the model’s discriminative power relative to RMCAIIA and IIB, while preserving the same underlying settlement logic. The classification still exceeds chance expectation on accuracy, but only marginally, and it does not do so on recall; the gap between training and validated performance widens appreciably with respect to the two preceding phases. This is the weakest of the four late models, and the pattern indicates that the core locational rules remain selective but are no longer sufficient to capture the full diversity of settlement situations emerging in this phase.

This shift is nevertheless embedded within a stable hierarchy of predictors. Both mean SHAP values and normalized split frequencies continue to identify topographic setting as the dominant driver of settlement location, its split frequency reaching the maximum value and its mean SHAP contribution remaining the highest of all variables. That contribution has, however, almost halved with respect to RMCAIIB and is well below the peak recorded in RMCAIIA: morphologically distinctive positions remain the principal attractors, but they discriminate less sharply than before. The hydrological variables move in the opposite direction. Proximity to rivers returns the second highest mean SHAP value of the phase and springs follow closely, values comparable to those of the earliest phases in the sequence. Altitude retains a modest positive effect, reinforcing the interpretation that elevation acts mainly through its coupling with topographic configuration. Also notable is soil class C4, suited primarily to grazing, which reaches its highest values of the whole sequence on both measures.

Taken together, these metrics suggest that RMCAIII does not represent an abandonment of the dominant settlement drivers, but a phase in which a well-established topographic logic coexists with an increasingly diversified set of site functions and roles. The convergence of a weakening topographic signal with a renewed contribution from hydrological variables and pastoral land points to a widening of the occupied niche rather than to its displacement.

A particularly illuminating example is provided by the coastal site of Piscina Torta [60–63]. Here, between the mid-eighth and the sixth century BCE, salt was very likely produced using the briquetage technique, a process requiring large quantities of seawater, fuel wood, and clay for the serial production of disposable ceramic containers, and ideally situated near a brackish lagoon from which naturally enriched brine could be obtained. The location of Piscina Torta near the terminal sector of the reconstructed Ostia lagoon was therefore dictated by the co-occurrence of these specific constraints. Such a choice clearly departs from the consolidated criteria governing residential settlement, dominated by topographic setting, and thus tends to elude the model’s predictions, lowering sensitivity/recall. At the same time, it provides compelling evidence that ecological niches were increasingly being occupied for purposes other than habitation.

In this sense, RMCAIII captures a stage in which the political landscape forged in earlier phases remains structurally intact, but becomes spatially more complex and articulated. Settlement placement continues to be anchored to topographic prominence, yet the increasing mismatch between the dominant pattern and the full set of occupied sites signals a system under pressure from demographic growth, urban expansion, and the diversification of socio-political roles. The reduced predictive completeness of the model thus mirrors a historical reality in which stability of principles coexisted with growing complexity of outcomes, marking the transition from a canalised protohistoric landscape to the more heterogeneous spatial organisation of the early historical period.

## Conclusions

This study demonstrates the value of Random Forest modelling, coupled with explainable AI techniques, as an exploratory framework for investigating long-term settlement dynamics in protohistoric landscapes. Rather than aiming at predictive mapping, the approach has been used here to disentangle the relative and diachronic importance of environmental factors shaping settlement choices. The combination of normalized split frequencies and SHAP values proved particularly informative: the former capturing the structural role of variables in partitioning the feature space, the latter quantifying the magnitude and direction of their contribution to site prediction. Together, these metrics identify both stable predictors and phase-specific shifts, while also highlighting situations in which variables act primarily as exclusionary or contextual filters.

At the same time, the analysis exposes clear limitations. The reliance on pseudo-absence points, the small number of sites in some phases, and the inherent spatial and taphonomic biases of the archaeological record inevitably constrain the robustness of performance metrics and the generalisability of results.

A further limitation concerns the form in which predictor effects have been examined, and the level of resolution at which the analysis is framed. All the measures reported here are marginal: they quantify the contribution of each variable considered on its own, and do not resolve whether the effect of one predictor is conditional on the value of another. Such interactions are archaeologically plausible, but estimating them requires resolving the model’s response over a two-dimensional grid, which the number of cases available for each phase does not support. Examining them would require either a substantially larger dataset or a deliberate reduction in resolution, by grouping some of the phases into broader chronological blocks. Such reconfigurations therefore pose different questions rather than refining the present ones, and each would require its own interpretation against the archaeological record. They represent a natural direction for further work.

Within these limits, the modelling results reveal a coherent long-term transformation in the environmental logic of settlement. From the Early Bronze Age, when hydrological accessibility dominated the discrimination between sites and non-sites, the system progressively reweighted towards topographic structure as the principal axis of differentiation. From MBA3 onwards, and especially from FBA12 through the RMCA phases, morphologically distinctive and elevated settings emerged as the dominant attractors, producing increasingly canalised and statistically separable settlement signatures. This diachronic shift from hydrologically anchored to topographically anchored settlement patterns is not merely an environmental trend, but reflects a deeper transformation in the social use of space, in which defensibility, visibility, and positional control became central to the organisation of inhabited landscapes.

Read from a historical perspective, the model captures the gradual emergence, consolidation, and eventual stabilisation of a politically structured landscape in *Latium vetus*. The exploratory and weakly differentiated patterns of the early phases give way, in MBA3, to the first coherent structuring of territory around defensible nodes. During the Recent and Final Bronze Age, this framework is progressively saturated and formalised, in parallel with growing social differentiation, the institutionalisation of inequality, and the appearance of elite lineages anchored to key places. In the RMCA phases, this logic crystallises into a mature territorial order, in which settlement placement becomes a normative and socially regulated act embedded within hierarchical or federative systems. The later decline in recall observed in RMCAIII does not indicate a breakdown of this logic, but rather its coexistence with increasing functional diversification, as specialised sites, such as coastal salt-production centres like Piscina Torta, occupy ecological niches defined by economic constraints rather than residential norms. In this sense, the growing mismatch between dominant locational rules and the full range of occupied sites mirrors a landscape likely under pressure from territorial saturation, demographic growth, and socio-political complexity.

Overall, the integration of Random Forest modelling with archaeological interpretation offers a powerful means to bridge ecological constraints and historically contingent choices. By making explicit the shifting dimensions along which settlements differ from their surroundings, the approach provides a quantitative backbone to long-standing qualitative narratives about the rise of defensible centres, territorial control, and early political organisation in central Italy. More broadly, this study illustrates how explainable machine learning can serve not as a substitute for archaeological reasoning, but as a complementary lens through which complex spatial histories can be explored, tested, and refined.

## Supporting information

S1 Text Soil classes. (DOCX)

S1 Fig Spearman rank-correlation matrix of the twelve environmental predictors. Pairwise Spearman’s ρ computed on the complete dataset (N = 208 settlements and pseudo-absence points), shown as a lower-triangular matrix; cell colour encodes the coefficient from −1 (blue) to +1 (red), and coefficients exceeding |0.7| are set in bold. Underlying values are reported in S8 Table.
(PNG)

S1 File Permutation test script. Python script reproducing the permutation tests and the training/validation comparison reported in S12 Table. It reads S11 Table directly, one sheet per model, and requires pandas, numpy, scikit-learn and openpyxl. The random seed is fixed, so the reported values are reproduced exactly.
(ZIP)

S2 File KNIME model. (ZIP)

S1 Table Environmental and classification data used in the Random Forest model. The table reports site ID, altitude (m a.s.l.), distances from sea, rivers, water bodies, and springs (in walking seconds/100), surface areas of soil classes C1–C6 (m²), and settlement classification (Yes = real settlement, No = simulated location).
(XLSX)

S2 Table Configuration of the KNIME nodes used in the Random Forest workflow (Fig 5). (XLSX)

S3 Table Sites of uncertain chronological attribution, by phase. One sheet per chronological phase, listing the sites that are plausibly but not securely attested in that phase. These sites appear on the corresponding phase maps (Figs 17–32) but were excluded from the Random Forest models, which admit only securely dated attributions; the cases actually entering each model are listed in S11 Table.
(XLSX)

S4 Table Score matrix of the Random Forest models trained with the optimised hyperparameters and a 1:1 ratio between settlements and pseudo-absence points. One row per model: the pooled model (ALL) and each of the nine chronological phases. The Objective value (Recall) column reports the cross-validated recall on which the hyperparameter selection was based, obtained from the grid search described in the Methods. All remaining metrics (Recall, Precision, Sensitivity, Specificity, F-measure, Accuracy and Cohen’s Kappa) derive from a second, independent model trained with those hyperparameters on 80% of the cases and evaluated on the held-out 20%.
(XLSX)

S5 Table SHAP values for individual cases. All settlements and pseudo-absence points, reported for both output classes and for the model confidence.
(XLSX)

S6 Table Mean SHAP values by chronological phase. Only real settlements and prediction = Yes.
(XLSX)

S7 Table Soil descriptions. The official descriptions are in Italian. The English translation has been provided by the author.
(XLSX)

S8 Table Spearman rank correlation matrix. (XLSX)

S9 Table VIF values. (XLSX)

S10 Table Normalized split frequencies by chronological phase. Split frequencies of the twelve environmental predictors in each of the nine phase-specific Random Forest models, normalised within each phase so that the most frequently used predictor takes the value 1.00. These are the values plotted in Figs 11–13.
(XLSX)

S11 Table Datasets used for the Random Forest models. One sheet per model: ALL for the pooled model and one for each of the nine chronological phases. Each sheet lists the cases included in that model — settlements and matched pseudo-absence points — with the environmental variables used as predictors.
(XLSX)

S12 Table Results of the permutation test. (XLSX)

S13 Table Mean absolute SHAP values for the pooled model. (XLSX)

S14 Table Distribution of the SHAP contributions underlying the variable-importance ranking. Three sheets, all computed over the complete set of 208 cases (settlements and pseudo-absence points) and expressed in units of predicted probability, referring to the positive class. *Quartiles* reports the mean contribution of each hydrological predictor within quartiles of walking distance from the nearest instance of the corresponding feature type. *Strongest contributions* compares, for each of those predictors, the median distance of the fifteen cases returning the highest SHAP values with the median across all cases. *Topographic setting* reports the number of cases, mean, minimum and maximum contribution for each of the six morphological categories.
(XLSX)
