# Supplementary material for: Tracing settlement dynamics in Latium vetus: Explainable machine learning perspectives from the Bronze to the Early iron age
Source: PLoS One. 2026 Sep 16;21(9):e0357054. doi: 10.1371/journal.pone.0357054 (PMC13580991; doi:10.1371/journal.pone.0357054)
Supplement: Supplementary file 1 [file pone.0357054.s001.docx]

## Classes of landscape units (descriptions of landscape units in S7 Table)

A1 (class 1): Holocene alluvial soils, usually deep, sometimes with a water table, relatively easy to work

A2 (class 3): Holocene alluvial soils with drainage problems, humic soils, potential swamps or marshes

B (class 4): Holocene slope-wash deposits, shallow and very stony soils on slopes

C1 (class N): Holocene dunes

C2 (class 5): Holocene dunes with traces of consolidation

D (class 4): Pleistocene and Holocene red soils

E (class 1): Terraced Pleistocene alluvial soils, deep soils

F1 (class 3): Pleistocene breccias and alluvial fans, shallow and very stony soils, slope less than 10%

F2 (class 4): Pleistocene breccias and alluvial fans, shallow and very stony soils, slope greater than 10%

G (class 5): Pleistocene dunes

H1 (class 2): Paleotiber 1 and 2, Pleistocene marls, slope less than 20%

H2 (class 4): Paleotiber 1 and 2, Pleistocene marls, slope greater than 20%

I (class 4): Travertines

L (class N): Lavas

M1 (class 2): Pozzolanas, deep soils, excellent water retention, easy to work, slope less than 10%, pozzolanas and earthy tuffs

M2 (class 3): Pozzolanas, deep soils, excellent water retention, easy to work, slope greater than 10%

N (class 2): Tuffites

O1 (class 3): Lithoid tuffs, shallow soils, slope less than 10%

O2 (class 5): Lithoid tuffs, peperino, shallow soils, slope greater than 10%

P1 (class 3): Miocene sandstones and macco, slope less than 10%

P2 (class 5): Miocene sandstones and macco, slope greater than 10%

Q (class 2): Miocene and Pliocene clays

R (class 5): Miocene and Paleogene benches

S1 (class 3): Marls, slope less than 10%

S2 (class 4): Marls, slope greater than 10%

T1 (class 4): Limestones, slope less than 10%

T2 (class N): Limestones, slope greater than 10%

U (class 2): Medium-depth soils on soft tuffs

All soils with slopes greater than 30% were assigned to class 6.
